# Peptidic Inhibitors and a Fluorescent Probe for the Selective Inhibition and Labelling of Factor XIIIa Transglutaminase

**DOI:** 10.3390/molecules28041634

**Published:** 2023-02-08

**Authors:** Eric W. J. Gates, Kian Mansour, Sahar Ebrahimi Samani, Sammir Shad, Mari T. Kaartinen, Jeffrey W. Keillor

**Affiliations:** 1 Department of Chemistry and Biomolecular Sciences, University of Ottawa, Ottawa, ON K1N 6N5, Canada; 2 Division of Experimental Medicine, Faculty of Medicine, McGill University, Montréal, QC H3A 0C7, Canada; 3 Faculty of Dental Medicine and Oral Health Sciences, McGill University, Montréal, QC H3A 0C7, Canada

**Keywords:** transglutaminase, factor XIII, fluorescent probe, enzyme inhibition, localization, immunofluorescence microscopy, cellular labelling

## Abstract

Factor XIIIa (FXIIIa) is a transglutaminase of major therapeutic interest for the development of anticoagulants due to its essential role in the blood coagulation cascade. While numerous FXIIIa inhibitors have been reported, they failed to reach clinical evaluation due to their lack of metabolic stability and low selectivity over transglutaminase 2 (TG2). Furthermore, the chemical tools available for the study of FXIIIa activity and localization are extremely limited. To combat these shortcomings, we designed, synthesised, and evaluated a library of 21 novel FXIIIa inhibitors. Electrophilic warheads, linker lengths, and hydrophobic units were varied on small molecule and peptidic scaffolds to optimize isozyme selectivity and potency. A previously reported FXIIIa inhibitor was then adapted for the design of a probe bearing a rhodamine B moiety, producing the innovative **KM93** as the first known fluorescent probe designed to selectively label active FXIIIa with high efficiency (*k_inact_*/*K*_I_ = 127,300 M^−1^ min^−1^) and 6.5-fold selectivity over TG2. The probe **KM93** facilitated fluorescent microscopy studies within bone marrow macrophages, labelling FXIIIa with high efficiency and selectivity in cell culture. The structure–activity trends with these novel inhibitors and probes will help in the future study of the activity, inhibition, and localization of FXIIIa.

## 1. Introduction

The transglutaminase (TGase) family of enzymes is comprised of eight calcium-dependent isozymes and the non-catalytically active erythrocyte membrane protein band 4.2 [1,2,3]. These enzymes carry out numerous functions in biological settings with a primary role in crosslinking proteins through the formation of N^ε^(ɣ-glutaminyl)lysine bonds using a Cys-His-Asp catalytic triad [4,5,6]. Within the TGase family are two isozymes of current therapeutic interest, transglutaminase 2 (TG2) and Factor XIII (FXIII). Human TG2, also referred to as tissue transglutaminase, is ubiquitously expressed throughout virtually all tissues [7,8]. TG2 is noteworthy due to its transamidase activity involved in liver fibrosis, its deamidation role in celiac disease, and its intracellular G-protein activity [9,10,11] which has been implicated in numerous cancer models including the epithelial-mesenchymal transition of cancer stem cells [12,13,14,15,16,17]. The major role of FXIII is within the final step of the coagulation cascade, where it mediates the crosslinking of insoluble fibrin monomers to form a rigid 3D blood clot network. This makes it a viable therapeutic target for the development of novel anticoagulant drugs in the treatment of venous thrombosis [18]. Current drugs, including heparins and coumarins, target a multitude of upstream clotting factors, thus preventing soft clot formation and increasing the risk for severe bleeding [19,20,21]. Inhibition of the downstream FXIII is believed to provide a milder alternative that would allow for soft clot formation, potentially achieving a blood thinning effect without inducing elevated bleeding risks [22]. Intracellular FXIII is additionally hypothesised to play roles in the activities of osteoblasts, monocytes, and macrophages, making the intracellular isozyme an appealing therapeutic target as well [23].

FXIII is most abundant in the blood and has roles in promoting clotting [24], wound healing [25], and angiogenesis [26]; however, it is also present in the cytosol of cells [27,28]. Plasma-soluble FXIII exists as FXIII-A_2_B_2_, an inactive hetero-tetramer composed of two catalytic A subunits and two carrier B subunits [29]. The classical activation of FXIII in the blood coagulation cascade begins with thrombin-catalyzed proteolytic cleavage of the N-terminal 37 amino acid activation peptide tethered to each A subunit [30,31]. The interaction of the A and B subunits then weakens and allows for calcium binding to the A subunits. The heterotetramer then dissociates into the A_2_ homodimer [32], which finally separates into the enzymatically active monomer of Factor XIIIa (FXIIIa) in the presence of a suitable substrate [33]. It is of note that cellular localised FXIII (FXIII-A) does not exist as a heterotetramer and the intracellular activity can be achieved by calcium binding [34].

**Figure 1:**
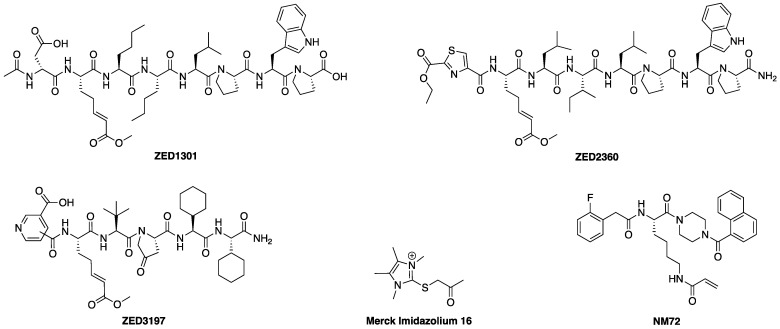
Chemical structures of Zedira’s ZED1301 [33], ZED2360 [41], ZED3197 [22], Merck’s imidazolium inhibitor 16 [39], and Keillor’s TG2 targeted small molecule inhibitor NM72 [42].

Interest in the development of FXIIIa inhibitors has blossomed in recent years due to the potential therapeutic applications highlighted above. Though progress in the FXIII inhibition field has been reviewed elsewhere [35], a general overview of the most relevant details is provided herein. FXIII has historically proven to be incredibly difficult to inhibit in a potent and selective manner. Most reported FXIII inhibitors tend to also inhibit TG2, which is believed to be the most problematic off-target enzyme due to its ubiquitous expression profile [36] and its roles in wound-healing and angiogenesis, which overlap with those of FXIII [37,38]. In the early 1990s, Merck set out to design imidazolium-based small molecule covalent inhibitors of FXIIIa, but these exhibited low selectivity (Figure 1) [39]. A novel peptidic sequence with a high affinity for FXIIIa was discovered in 2006 through phage-display screening [40]. Replacement of this peptide’s glutamine residue with an electrophilic warhead, namely an α,β-unsaturated methyl ester or glutamate-derived Michael acceptor (MA), allowed Zedira to produce irreversible inhibitor ZED1301, which covalently inactivates the catalytic Cys thiolate of FXIIIa. This compound shows relatively high potency (IC_50_ = 110 nM) and moderate selectivity (30-fold) over TG2 [33]. Zedira further optimised ZED1301 into inhibitors such as ZED2360 (IC_50_ = 29 nM) [41] and peptidomimetic ZED3197 (IC_50_ = 16 nM, 19-fold selectivity for FXIIIa over TG2). The latter compound was successfully evaluated in rabbit coagulation models and has shown the most promise of any FXIIIa inhibitor. However, it shows a short half-life of 5–10 min in rabbit models, requiring high doses (6–54 mg/kg ZED3197) administered through intravenous bolus and continuous infusions to maintain adequate plasma levels [22]. There thus remains a need to develop more potent, selective, and drug-like inhibitors of FXIIIa as potential anti-coagulants.

While Factor XIIIa therapeutics remains an active area of research, the chemical tools currently available for studying this crucial clotting enzyme are comparatively limited. To our knowledge, only two probes for monitoring the activity of FXIIIa in biological systems have been reported to date [43,44]. Both probes serve as α_2_-antiplasmin substrate mimics and are tagged with either a near-IR fluorophore or Gd-chelating magnetically resonant contrast moiety. These compounds label blood clots through FXIIIa-mediated incorporation into fibrin, allowing for the indirect deduction of FXIII activity and localization. No known probes have been reported to specifically label the active form of the enzyme itself; the development of such a tool would aid in the study of the localization, migration, and activity of FXIII in cellulo.

In our work on TGases, we developed numerous activity assays, probes, and targeted covalent inhibitors that show high isozyme selectivity, mainly within the context of studying TG2 [42,45,46,47,48,49,50]. In the current work we design, synthesize, and evaluate inhibitors of FXIIIa, and use the optimised inhibitor design in the production of a fluorescent probe. In order to achieve the desired isozyme selectivity for FXIII over TG2, we relied upon previous kinetic data and the binding pocket differences between FXIIIa [33,41,51] and TG2 [42,45,46,52] to design several series of peptidic and small molecule inhibitors. More specifically, we explored structure–activity relationships (SAR) with respect to the linker length, electrophilic warhead functionality, hydrophobic moiety, and acidic group, while keeping the scaffold backbone constant. Evaluation of the peptidic and small molecule inhibitor series exposes inconsistent and scaffold-dependent trends, potentially due to conformational dynamism within the active site of the enzyme [41] akin to that observed recently in the TG2 field [45]. We then further modified the most potent scaffold to incorporate a fluorescent moiety, thereby creating a high-affinity fluorescent probe for the specific labelling of FXIIIa within biological settings. The fluorescent probe was assayed in cell culture to display its effectiveness at labelling FXIIIa in cellulo.

## 2. Results and Discussion

### 2.1. Structure Design

#### 2.1.1. Design of Peptidic Inhibitors of FXIIIa

**Figure 2:**
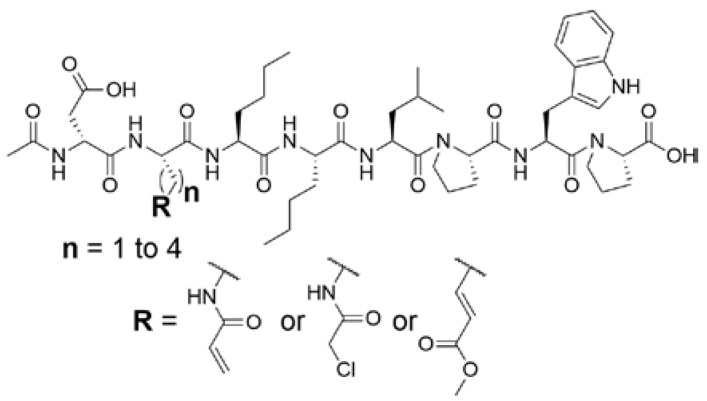
Design of the peptidic FXIIIa inhibitor series with various linker lengths and electrophilic warhead functionalities.

Although extensive SAR studies have been performed on the amino acid sequences of the ZED3197 and ZED1301 scaffolds, the crucial warhead residue remains less explored [51,53,54]. With respect to the electrophilic moiety itself, it is noteworthy that the α,β-unsaturated ester warhead is a common feature among Zedira’s inhibitors, while other amide-based electrophiles, such as acrylamides and α-chloroacetamide, had not apparently been tested on these peptidic scaffolds. This is surprising as both these amide-based warheads have shown great success in achieving potent transglutaminase inhibition while remaining stable to degradation by glutathione in the cell [55]. Furthermore, the distance between the scaffold and electrophile, hereinafter referred to as the linker length, was not varied in the Zedira FXIII inhibitor studies available in the literature [51,53,54]. We found that the linker length has a profound impact on peptidomimetic TG2 inhibitor potency, with longer linkers being more efficient than shorter ones [46]. Combined with crystal structure evidence that the active site tunnel leading to the catalytic Cys thiolate is shallower in FXIII than in TG2 [33,52], we hypothesised that the decreasing linker length may be a viable method of increasing potency of FXIIIa inhibition and increasing selectivity over TG2. Thus, a series of peptidic FXIIIa inhibitors with three different electrophilic warheads (α,β-unsaturated ester, acrylamide, and α-chloroacetamide) and four linker lengths (one through four methylene units) was designed in order to investigate the impact of these structural features on the potency of FXIIIa inhibition and selectivity over TG2 (Figure 2). The ZED1301 peptide scaffold was selected for this study due to its known affinity for FXIIIa and ease of synthesis. ZED1301 itself was also independently synthesised and evaluated in order to supplement its original kinetic characterization, a condition-dependent IC_50_ value [33]. Condition-independent *k_inact_* and *K*_I_ values were acquired for more accurate comparisons with irreversible FXIIIa inhibitors developed herein and in other works.

#### 2.1.2. Design of Small Molecule Inhibitors of FXIIIa

**Figure 3:**
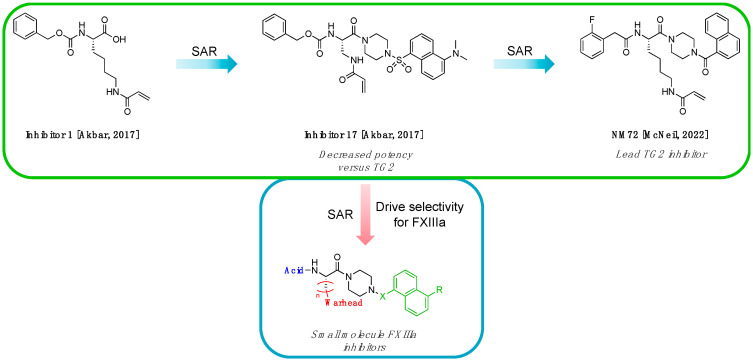
Design approach for small-molecule inhibitors of FXIIIa, based on a known low potency TG2 “inhibitor 17” and driving selectivity for FXIIIa with N-terminal acids and C-terminal hydrophobic groups [42,46].

In the hope of developing a FXIII inhibitor with better drug-like properties than the peptide-based inhibitors investigated herein, we also screened a series of small-molecule compounds. Stemming from our extensive research on small-molecule TG2-selective inhibitors, we commenced our search for small-molecule FXIIIa inhibitors by using a previously published scaffold that exhibits low potency against TG2, again noting that the short linker length produces poor TG2 inhibition [46]. Two key elements from the reported high-affinity peptidic sequences [46] for FXIIIa were adapted into the small molecule inhibitor design and retained across our series of compounds, specifically the negatively charged N-terminal moiety and hydrophobic C-terminus. Thus, our SAR work in this small molecule FXIIIa inhibitor investigation encompassed a variety of N-terminal acids and C-terminal hydrophobic units that have shown promise in previous TGase studies (Figure 3) [46]. Small changes in the linker length were also investigated. The distance between the warhead and scaffold was reduced further through incorporation of a D-Dap warhead-bearing residue, allowing the coupling of the acid to the sidechain amine and the warhead to the α-amine. Given the broad scope of the structural features explored in this work, the known reactivity of the acrylamide towards TGases, and its superior presumed stability and selectivity compared to chloroacetamides and esters, the acrylamide warhead was mostly retained throughout the small-molecule FXIIIa SAR. A few derivatives bearing α,β-unsaturated methyl esters, akin to the Zedira peptides, were also synthesised and evaluated in order to provide a preliminary warhead comparison within this small molecule scaffold and between series with the peptidic compounds developed.

#### 2.1.3. Design of Fluorescent Probe of FXIIIa

**Figure 4:**
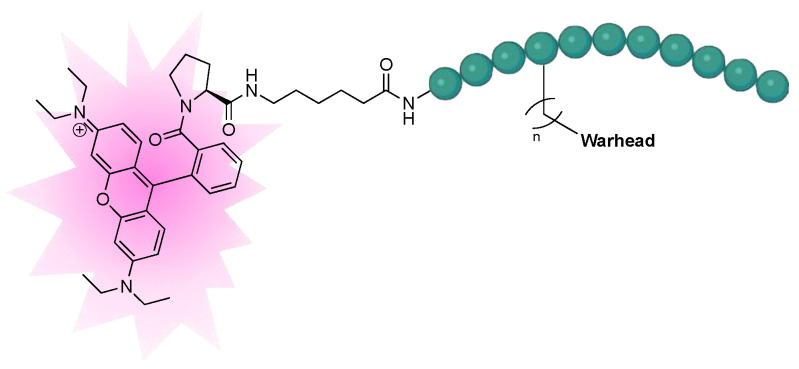
Design of fluorescent probe for FXIIIa tethered to the optimised inhibitor structure.

After completion of the synthesis and evaluation of the peptidic and small-molecule FXIIIa inhibitors, the optimised linker, warhead, and scaffold were used to design a rhodamine B labelled probe for studying FXIIIa. Rhodamine B was attached at the N-terminus through a flexible 6-aminohexanoic acid linker to allow the bulky fluorophore to be held far away from the binding site. The rhodamine B fluorophore was chosen due to its desirable, bright red emission, and minimal overlap with background cellular autofluorescence. It was also linked through a proline residue since the tertiary amide linkage allows the rhodamine to preserve its intrinsic fluorescence (Figure 4).

### 2.2. Synthesis

The general synthesis of the inhibitors disclosed herein was achieved through a combination of solid-phase peptide synthesis (SPPS) and in-solution chemistry. For assembly of the peptidic inhibitors bearing amide-based warheads, amino acid monomers with varying linker lengths leading to the appropriate warheads, or precursors thereof, were synthesised and subsequently incorporated into the linear octapeptides. The syntheses of the unsaturated ester-bearing peptides were carried out in a manner similar to that reported by Zedira [51,53]. The small molecule inhibitors were synthesised through careful in-solution manipulations of protecting groups, allowing for the sequential installation of first the various hydrophobic groups and next the electrophilic warhead. These key intermediates with free N-termini were accumulated, allowing for divergent functionalization to final inhibitors bearing different N-terminal acidic moieties. A detailed discussion of the inhibitor syntheses, as well as full experimental procedures and characterization data, is provided in the Supplementary Materials, along with Schemes S1–S21. Between the peptidic and small molecule scaffold series, a total of 22 inhibitors and 1 probe were synthesised and evaluated for inhibition of FXIIIa.

### 2.3. Kinetic Evaluation

**Figure 5:**
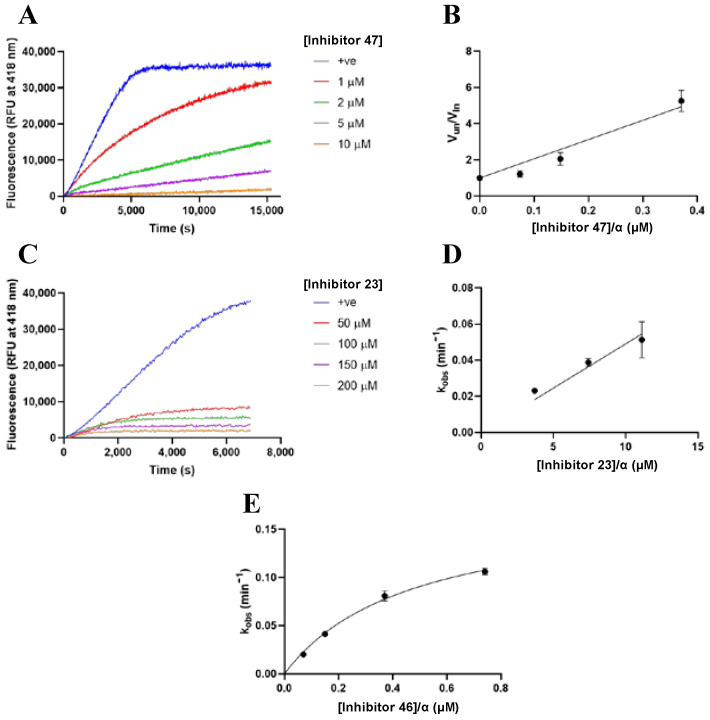
Representative kinetic traces and data fitting for TGase inhibition assays. (**A**) Raw blank-subtracted kinetic traces of FXIIIa activity with substrate A101 and increasing concentrations of reversible inhibitor **47** (0, 1, 2, 5, 10 µM). (**B**) Dixon plot fitting of initial rates of FXIIIa activity versus inhibitor concentration divided by alpha for inhibitor **47** to obtain *K*_i_. (**C**) Raw blank subtracted kinetic traces of FXIIIa activity with substrate A101 and increasing concentrations of irreversible inhibitor **23** (0, 50, 100, 150, 200 µM). (**D**) Linear regression of saturation fitting of *k_obs_* versus inhibitor concentration divided by alpha for irreversible inhibitor **23** to obtain a ratio of *k_inact_*/*K*_I_. (**E**) Saturation fitting of *k_obs_* versus inhibitor concentration for irreversible inhibitor **46** displaying inhibition saturation kinetics to extract both *k_inact_* and *K*_I_ parameters.

All inhibitors synthesised herein were evaluated for inhibition against FXIIIa, using the fluorescence-quenched A101 isopeptidase assay [56,57], and against TG2, using the colorimetric AL5 transamidase assay [46,50]. The assay substrates A101 and AL5 both present labile bonds that are cleaved by the activities of their corresponding transglutaminases, resulting in the release of a fluorophore or chromophore moiety, respectively. The rate of consumption of these assay substrates, indicated by increases in fluorescence or absorbance over time, can be detected for measuring enzyme activity. Assays were run in duplicate at constant reporter substrate concentration with varying concentrations of inhibitor. Representative fluorescence-time plots are shown in Figure 5A,C. The type of inhibition was determined through visual inspection of the fluorescence-time curvature; termination of the enzymatic reaction with the substrate at a plateau lower than the no-inhibitor positive control is indicative of irreversible inhibition. On the other hand, convergence to a common plateau indicates reversible inhibition.

Irreversibility was tested further through substrate spike experiments. As shown in Supplementary Materials Figures S1–S3, an additional substrate was added to inhibited and uninhibited enzyme reactions after the completion of the positive control reaction. Further increases in fluorescence upon substrate spike, as in Figure S1, indicate reversibility of inhibition, while the lack of an additional increase, as in Figure S2, suggests that all enzyme has been irreversibly inhibited. Analysis was carried out through corrected Dixon modelling in the case of reversible kinetics, to obtain *K*_i_ values (Figure 5B), and hyperbolic saturation modelling was used to obtain *k_inact_* and *K*_I_ for inhibitors showing irreversibility (Figure 5E) [58,59]. For cases in which the tested inhibitor concentrations were not high enough to reach saturation, a linear regression provided an estimate of the inhibitor’s *k_inact_*/*K*_I_ ratio (Figure 5D). Further details concerning data collection and analysis are provided in the Materials and Methods section.

#### 2.3.1. Kinetic Evaluation of Peptidic Inhibitors of FXIIIa

The structures of the 11 peptidic inhibitors synthesised in this work are shown in Table 1. All kinetic data from their evaluation are summarised in Table 2. Several interesting trends can be noted when investigating the impact of the warhead functionality and linker length on the potency of FXIIIa inhibition. While the acrylamide-bearing inhibitors **12–14** show reversible competitive FXIIIa inhibition, the analogous chloroacetamides **21–23** operate in an irreversible manner. Since both warheads are known to be capable of covalent bond formation in the presence of a suitable thiolate, the observed differences in inhibition mode with FXIIIa must be due to geometry rather than intrinsic reactivity. Perhaps binding of the warhead carbonyl into the enzyme’s oxyanion pocket places the acrylamide’s electrophilic carbon too distant from the thiolate, preventing S-C bond formation, while that of the chloroacetamide, being one position closer to the carbonyl, is just close enough to allow for covalent bond formation. It is also interesting to note that the acrylamide and chloroacetamide series show similar linker length trends. Potency is relatively insensitive to changes in the linker length between 2 through 4 methylene units, as the *K*_i_ values for acrylamides **12–14** are of the same order of magnitude, and the same can be said for the *k_inact_*/*K*_I_ ratios of chloroacetamides **21–23**. However, in both cases, reducing the linker down to 1 methylene, as in compounds **11** and **20**, results in a complete loss of inhibition. Since the electrophilic carbon placement is different between compounds **11** and **20**, and that of **11** matches **21**, a known inhibitor with an f-position electrophilic carbon, it is clear that the lack of inhibition cannot be explained by improper electrophile placement. Instead, improper carbonyl carbon placement may be responsible for the lack of FXIIIa inhibition in compounds **11** and **20**. Both place the carbonyl carbon at the d-position, which is apparently too close to the scaffold, and may not allow for appropriate binding in the enzyme’s active site.

ZED1301, the lead compound (**46**), was found to be the most potent irreversible inhibitor of the series, with *k_inact_*/*K*_I_ ratio several orders of magnitude higher than any of the chloroacetamides. The superior potency of ZED1301 (**46**) can be attributed to its unique warhead structure, as the unsaturated ester was the only warhead studied in this work that places the electrophilic carbon closer to the scaffold than the more distal carbonyl. It is believed that this geometric arrangement allows this inhibitor to take full advantage of the non-covalent oxyanion hole interactions and covalent S-C bond formation, as shown in the crystal structure published by Zedira [33], while inhibitors with the acrylamide and chloroacetamide warheads may be unable to form these interactions due to excessive induction of strain required in the enzyme or inhibitor. This work also represents the first report of condition-independent kinetic parameters, *k_inact_* and *K*_I_, for ZED1301 (**46**), which will allow for accurate comparisons with inhibitors developed in this work and other studies. Either decreasing or increasing the linker length from ZED1301 results in a loss of inhibitory potency and changes to inhibition mode. The shorter-linker ester-bearing compound **45** shows weak reversible inhibition similar in potency to that seen with acrylamides **12**–**14**, while the longer-linker ester-bearing inhibitor **47** displays highly potent reversible FXIIIa inhibition. These results further support the strict geometric requirements for binding and covalent bond formation in the active site of FXIIIa. As for the impact of linker and warhead on selectivity over TG2, no clear trends were noted. While none of the acrylamides result in any noticeable TG2 inhibition up to 350 μM, most of the chloroacetamides and ester-bearing inhibitors are irreversible TG2 inhibitors. The lack of inhibition seen with compounds **21** and **45**, as well as the superior potency of the ester warhead, are attributed to geometrical factors governing the enzyme-inhibitor interaction. With the peptidic inhibitors fully evaluated and with ZED1301 (**46**) identified as the most potent of the series, we then evaluated our small molecules as potential selective FXIIIa inhibitors.

#### 2.3.2. Kinetic Evaluation of Small Molecule Inhibitors of FXIIIa

Kinetic evaluation of the small molecule inhibitor library began with the dansyl hydrophobic series inhibitors **71**, **73**–**75**, and **79** (Table 3). The dansylated inhibitor scaffold was desirable as it could act in a multi-faceted role as a potent inhibitor scaffold as well as a fluorescent probe which would allow for FXIIIa localization studies. An investigation into the preferences of various alkyl acids revealed that sulfonyl **79** provided the greatest affinity with an apparent *K*_i_ of 26.7 ± 1.3 µM (See Supplementary Materials). However, upon analysis of the similarity in the kinetic results (of both the acid variants and the methyl ester **73)**, some doubts were raised regarding the assay conditions. A substrate spike of the inhibition assays revealed that the FXIIIa was still active, and the inhibitors were not acting in an irreversible manner; potentially implying a reversible mechanism (Supplementary Materials Figure S3). Spiking the **79**-inhibition assay with another dose of inhibitor **79** caused a noticeable decrease in apparent RFU (Supplementary Materials Figure S4). Due to the potential fluorescent quenching of both acrylamide [60] and the fluorescent dansyl moiety of the inhibitor scaffold, they were both evaluated in the inhibition assay. Dansyl amide resulted in nearly identical traces as those obtained using the dansylated inhibitor library (Supplementary Materials Figure S5). A further spectrophotometric analysis of both dansyl and anthranilic acid in the literature revealed that the dansyl absorption peak and anthranilic acid excitation at 313 nm overlap perfectly. Since A101, as well as the similar substrate A138, are the standard continuous assays for FXIIIa activity [56,61] and were found to be incompatible with our fluorescent dansyl inhibitors, the inhibition data for these compounds were set aside, and the scaffold was optimised to eliminate the fluorescence interference effect.

Sulfonyl-naphthalene derivative **76** provided a *K*_i_ of 64.3 ± 5.1 µM (Supplementary Materials); however, this scaffold also produced an absorption band that overlaps with the excitation of A101. Further tailoring gave rise to the naphthoyl derivatives **72** and **77** that do not have photochemical properties that interfere with the fluorescent assay. Succinyl derivative **77** was the lead inhibitor from the L-Dap naphthoyl series, with a *K*_i_ of 69.0 ± 4.1 µM. An attempt to decrease the linker length to the warhead by one methylene unit, with the incorporation of the D-Dap residue **84**, resulted in the *K*_i_ rising to a value of 107.6 ± 19.1 µM. This contradicted our initial intuition that shorter linkers would improve potency against FXIIIa. Since none of the acrylamide-bearing inhibitors in this series led to irreversible inhibition but were clearly binding to the enzyme’s active site, two compounds bearing an unsaturated ester warhead (**87** and **88**) were evaluated. We hoped that the replacement of the acrylamide warhead with the Michael acceptor would result in an irreversible small molecule inhibitor of the enzyme. To our surprise, both the succinic derivative **87** and phthalic acid derivative **88**, with *K*_i_ values of 131.1 ± 7.9 µM and 265.0 ± 46.8 µM respectively, had reduced potency versus FXIIIa compared to the acrylamide-bearing compounds. A drastic drop in selectivity was also observed for these scaffolds, such that they were more selective for TG2, with irreversible *K*_I_ values of 14.3 ± 7.0 µM and 21.2 ± 9.0 µM, respectively. This unexpected finding hints at some scaffold dependence for the ideal warhead and linker combination for achieving potent, irreversible FXIIIa inhibition. While it was found that a linker length of 1 methylene on the peptidic scaffold and an acrylamide warhead (compound **11**) leads to no inhibition, the analogous small-molecule compound **77** shows reversible inhibition with *K*_i_ = 69 μM. Similarly, while **46** (ZED1301) shows the most potent irreversible FXIIIa inhibition in this work, the small molecule with the same two-methylene linker and unsaturated ester warhead in compound **87** shows only weak reversible inhibition with *K*_i_ = 131 μM.

This finding is mirrored perfectly by TG2 investigations in our group, in which we found that longer linkers bearing acrylamides on a peptidomimetic TG2 scaffold led to more potent inhibition than shorter ones [46]. However, more recently we showed that on an abbreviated small-molecule TG2 scaffold, the shortest linker achieves the most potent inhibition [45]. We hypothesize that a significant degree of conformational dynamism exists in the TG2 binding pocket, creating a scaffold dependence for the ideal linker length, based on the enzymatic conformation induced by the binding of different-sized substrates or inhibitors. We believe that a similar phenomenon is at play here with FXIIIa and our inhibitors disclosed herein. It is clear that the two-methylene linker and ester warhead, which are ideal on the peptide, are not as effective on the small molecule scaffold. This notion is further supported by Zedira’s recent study revealing a transient hydrophobic pocket in FXIIIa’s active site [41]. It appears that trial-and-error may be required to perfectly tailor the linker and warhead for each new scaffold size tested for FXIIIa and that further screening of linkers and warheads on this known binding small molecule scaffold may potentially produce an irreversible, drug-like, small molecule FXIIIa inhibitor.

Since no novel sub-10 µM *K*_I_ small molecule inhibitors were developed and **46** (ZED1301) remains the most potent irreversible FXIIIa inhibitor evaluated in this work, we opted to adapt this peptidic scaffold for a potential role as a novel fluorescent probe research tool.

#### 2.3.3. Kinetic Evaluation of Fluorescent Rhodamine B FXIIIa Probe

**Figure 6:**
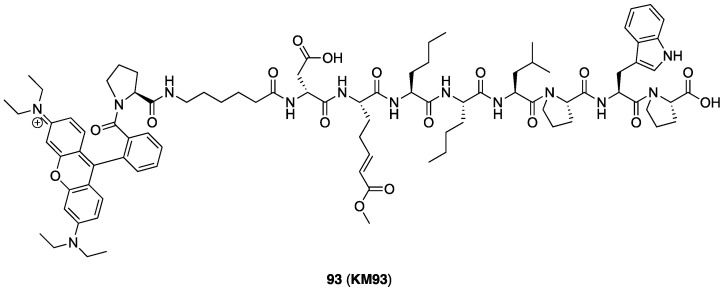
Structure of the rhodamine B incorporated FXIIIa probe **93** (aka **KM93**).

Fluorescent probe **93** (aka **KM93**, Figure 6), whose design incorporates a two-methylene linker, ester warhead, and peptide scaffold from ZED1301, retains irreversible FXIIIa inhibition with acceptable potency for biological applications. The *k_inact_*/*K*_I_ ratio against FXIIIa (127,300 ± 2890 M^−1^ min^−1^) is approximately three-fold lower for the probe than for the parent ZED1301 (**46**), showing that some potency was lost with the incorporation of the rhodamine B moiety and flexible linker. Fortunately, selectivity was maintained, as the probe remains around 6.5-fold selective for FXIIIa over TG2 (*k_inact_*/*K*_I_ = 19520 ± 2180 M^−1^ min^−1^).

### 2.4. Irreversible Labelling of Purified FXIIIa by SDS-PAGE

Labelling of commercially available FXIIIa with the rhodamine B fluorescent probe **KM93** was then evaluated by SDS-PAGE analysis. FXIIIa was incubated in the presence of 30 µM **KM93** and calcium; SDS-PAGE analysis of the resulting sample revealed a protein band that was red fluorescent (Figure S6). Coomassie staining then showed that this band corresponds to a molecular weight of ~80 kDa—coinciding with that of the commercially available FXIIIa [30,62]. The appearance of this red fluorescent band, on a gel run under denaturing conditions, allowed us to conclude that **KM93** successfully labelled FXIIIa in a robust, covalent, and irreversible manner.

### 2.5. Labelling of FXIIIa in Bone Marrow Macrophages

Covalent labelling of FXIIIa by **KM93** was tested in cell culture in murine bone marrow macrophages (BMM). BMMs are ‘gold standard’ expressors of FXIIIa and are responsible for the production of circulating FXIIIa [63]. Labelling of FXIIIa was first performed in cell lysate using 0–20 µM **KM93** and evaluated via SDS-PAGE, using fluoroimaging of the rhodamine B moiety. Supplementary Materials Figure S8A shows detectable, concentration-dependent covalent incorporation of **KM93** to a band at 80 kDa, with strongest labelling at 8-µM and 20-µM concentrations of the probe over both 6-h and 48-h incubations. A background band was detected at ~100 kDa, in the control as well as the test group, but the identity of this protein is unknown. Coomassie staining of the gel confirms equal loading (Figure S8B).

**Figure 7:**
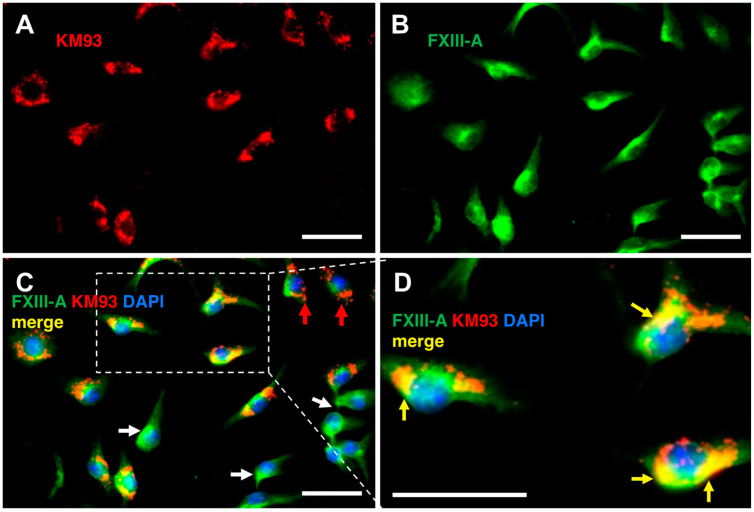
Immunofluorescence visualization of the labelling of FXIIIa by **KM93** in murine bone marrow macrophages (BMM). Cells were incubated with 20 µM **KM93** for 48 h in microscopy chamber slides. At the endpoint, cells were fixed and stained or treated with sheep anti-human FXIII-A antibody, followed by a secondary antibody, donkey anti-sheep AlexaFluor^®^-488 detection (green, Panel (**B**)). Nuclei were visualised with DAPI staining (blue). **KM93** was visualised at 568 nm (red, Panel (**A**)). All cells were positive for FXIIIa, incorporation of **KM93** into cells was clear and the probe colocalized with FXIII-A (Panel **C** and yellow arrows, inset Panel (**D**)). A subset of the macrophages did not incorporate the probe, despite the presence of FXIII-A (white arrows, Panel (**C**)), indicating that the enzyme may not be active in these specific macrophages. Some cells also showed strong red fluorescence but weak green fluorescence, giving the appearance of little colocalization, despite both probe and enzyme being present at these cellular compartments (red arrows, Panel (**C**)). White magnification bar represents 20 µm.

Fluorescent microscopy of the labelling showed rapid clear incorporation of **KM93** into BMM cells at 20 µM concentration after 48-h incubations (Supplementary Materials Figure S9 and Figure 7A). Labelling of Factor XIIIa was deemed to be intracellular (Figure 7A) as confirmed by co-staining with actin (Figure S9) and showed colocalization with an FXIII-A antibody (yellow arrows, Figure 7D). Not all FXIII-A antibody labelling was colocalized with the labelling by **KM93** (white arrows, Figure 7C). Since labelling by **KM93** is based on the enzyme activity of FXIIIa, this result suggests that Factor XIII may be present but not active in these specific macrophages.

An additional experiment was performed in order to confirm the specificity of labelling by **KM93**. Cultured BMM cells were first treated for 2 h with ZED1301 (**46**), a known inhibitor of Factor XIIIa. The cells were then treated with fluorescent probe **KM93** for an additional 4 h, which was followed by washing and imaging. Negligible fluorescent labelling was observed, relative to cells that were not blocked with ZED1301 prior to being treated similarly with **KM93** (see Supplementary Materials, Figure S10). This strongly suggests that **KM93** only reacts with the same cellular target as ZED1301 and increases our confidence in the specificity of **KM93** for labelling FXIIIa.

## 3. Conclusions

In this work, we set out to explore structure–activity relationships for the linker length and warhead functionality on a peptidic scaffold, as well as for the linker length, hydrophobic group, and acidic group on a small-molecule scaffold. While none of our compounds display improved potency over the lead inhibitor **46** (ZED1301), we showed that the mode and potency of FXIIIa inhibition are highly dependent on both the linker length and warhead functionality and that the optimal combination of these features may be scaffold-dependent due to the conformational dynamism in the enzyme. This work also represents the first report of the condition-independent kinetic parameters for ZED1301 (**46**). These findings set the stage for further developments in FXIIIa inhibitors as potential anticoagulants, as it is believed that the optimization of linkers and warheads on a small-molecule scaffold may be able to provide a drug-like inhibitor. The structure of ZED1301 was also adapted for the design of the rhodamine B-labelled fluorescent probe **93** (aka **KM93**), which was shown to retain irreversible inhibitory activity against the enzyme, to be effective at labelling FXIIIa in cellulo, demonstrating its applicability for localization studies in the further study of the biological roles of FXIIIa.

## 4. Materials and Methods

### 4.1. Kinetic Evaluation of FXIIIa Inhibition

A previously reported fluorescence-quenched isopeptidase assay using the commercially available substrate A101 (Zedira GmbH, Darmstadt, Germany) was employed for the determination of FXIIIa inhibition kinetics [56,57]. All assays were performed at 37 °C on 96-well plates, with fluorescence (excitation at 313 nm, emission at 418 nm) measured continuously using a BioTek Synergy 4 plate reader. For each inhibitor, duplicate trials were run in parallel at 4 inhibitor concentrations, along with duplicate positive controls (no inhibitor) and negative controls (no enzyme and no inhibitor). First, 125 μL of aqueous buffer 1 (111 mM Tris, 16.7 mM CaCl_2_, 333 mM NaCl, pH 7.5) and 18 μL of aqueous buffer 2 (55.6 mM TCEP, 149 mM H-Gly-OMe, pH 7.5) were added to each well, along with 5 μL of A101 stock solution (4000 μM in DMSO). Next, the appropriate amount of the desired inhibitor, dissolved in DMSO, was added, in addition to the amount of distilled water necessary to reach a total volume of 180 μL in each well. Negative controls were diluted to a final volume of 200 μL. Thrombin-activated human FXIIIa (T070, Zedira GmbH, Darmstadt, Germany), stored at −80 °C in 20-μL aliquots (1 mg/mL enzyme) of storage buffer (50.0 mM Tris, 1.00 mM TCEP, 150 mM NaCl, pH 7.5) was warmed to room temperature and diluted to 0.0741 mg/mL through the addition of 250 μL of storage buffer. The diluted enzyme solution was then aliquoted into 10 separate PCR tubes. The assay plate and enzyme tubes were subsequently incubated at 37 °C for 10 min. After the completion of the incubation period, 20 μL of diluted FXIIIa solution was added from each PCR tube to all wells in the plate excluding the negative controls using a multi-channel pipette. This produced a final volume of 200 μL per well, with final reaction conditions of pH 7.5, 74.4 mM Tris, 10.4 mM CaCl_2_, 223 mM NaCl, 5.10 mM TCEP, 13.4 mM H-Gly-OMe, 100 μM A101, and 7.41 μg/mL (94 nM) FXIIIa. The range of inhibitor concentrations tested spanned from 1 to 200 μM, and the final DMSO concentration in the reaction mixture was kept below 10% *v*/*v*. Monitoring of fluorescence emission was commenced after quick stirring by aspiration. Assays were terminated once the fluorescence had plateaued.

### 4.2. Kinetic Evaluation of TG2 Inhibition

A colorimetric transamidase assay using chromogenic substrate AL5 (Cbz-Glu(ɣ-*p*-nitrophenyl ester)Gly-OH) was used to determine TG2 inhibition kinetics as described previously [42,45,46,50]. In brief, assays were conducted at 25 °C in 96-well plates, and absorbance at 405 nm was monitored continuously using a BioTek Synergy 4 plate reader. For each compound, 6 inhibitor concentrations were tested, along with positive (no inhibitor) and negative (no enzyme, no inhibitor) controls. All assays were run in duplicate as separate independent experiments. Human TG2 expressed and purified as described previously [64] and stored in aqueous buffer at −80 °C, was first thawed and diluted to a working concentration of 50 mU/mL in the buffer. The diluted enzyme solution was stored on ice. The appropriate amount of distilled water, 125 μL of aqueous buffer (111 mM MOPS, 15.6 mM CaCl_2_, pH 6.9), the desired amount of inhibitor (from a DMSO stock solution), and 5.0 μL of AL5 (from a 5.56 mM DMSO solution) were added in that order to 7 Eppendorf tubes to reach final volumes of 250 μL per tube. After mixing thoroughly, 180 μL of the mixture from each tube was transferred to plate wells. To initiate the assay, 20 μL of the diluted TG2 solution was added using a multichannel pipette to each well except the negative control, which was treated with water. The final reaction conditions were pH 6.9, 50.0 mM MOPS, 7.0 mM CaCl_2_, 100 μM AL5, and 5 mU/mL TG2. The range of inhibitor concentrations tested spanned from 1 to 350 μM, and levels of DMSO were kept below 10% *v*/*v*. Data collection was then initiated after mixing by quick aspiration, and the assay was allowed to run for 20 min.

### 4.3. Determination of Type of Inhibition

The type of inhibition for both TG2 and FXIIIa was primarily determined by visual inspection of kinetic traces. Inhibitors producing fluorescence-time or absorbance-time kinetic curves that either reached plateaus at the same level as that of the positive control or did not plateau at all throughout the time course of the experiment, even at high inhibitor concentrations, were deemed to be operating through a reversible mechanism. This assumption was tested and validated with inhibitor **47** through an A101 substrate spike experiment. Additional A101 (5 μL of 4000 μM stock in DMSO) was added to enzymatic reactions lacking in inhibitor (positive control) and pre-treated with inhibitor (50, 100, 150, 200 μM) after the initial reactions were complete and fluorescence plateaus had been reached. Data collection at 37 °C was re-commenced immediately after the addition, and fluorescence was monitored over time as previously described. On the other hand, inhibitors producing kinetic curves in which earlier and lower plateaus in fluorescence or absorbance were observed as inhibitor concentration was increased were assumed to be operating through irreversible time-dependent covalent inactivation. This assumption was tested and validated with inhibitor **23** through an A101 substrate spike experiment in which an additional 5 μL of A101 substrate stock solution (4000 μM in DMSO) was added to the positive control and highest inhibitor concentration (200 μM) reaction. In the case of the small molecule inhibitors, an A101 spike experiment was conducted as outlined above, or an inhibitor spike with **79** the substrate spike was replaced with the highest concentration of inhibitor being assayed. Analysis was carried out as described above.

### 4.4. Analysis of In Vitro Kinetic Data

Data analysis for the determination of kinetic inhibition parameters for both FXIIIa and TG2 was performed using Microsoft Excel and GraphPad Prism. Absorbance-time and fluorescence-time plots from all positive controls and inhibitor treatments were corrected for background substrate hydrolysis through subtraction of the negative control and were then set to an initial fluorescence or absorbance of zero through subtraction of y-intercepts at time zero from all points.

For inhibitors displaying reversible competitive kinetics, the first 10% conversion, taken as the time point at which the relative fluorescence or absorbance reached 10% of the maximum at the plateau, was used to obtain the initial rates from linear regressions of fluorescence or absorbance over time. The means and standard deviations from duplicate trials of the ratios of uninhibited (positive control) to inhibited initial rates (*ν_un_*/*ν_in_*) were then plotted against the corrected inhibitor concentrations ([*I*]/*α*), producing a normalised Dixon plot defined by the relationship

(1) $$\frac{\nu_{un}}{\nu_{in}}=\frac{1}{K_{i}}\left(\frac{\left[I\right]}{\alpha }\right)+1$$

Inhibitor concentrations were corrected by division with the parameter α, defined as the constant correcting for competition between the substrate and inhibitor for the enzyme’s active site. Based on the known *K*_M_ of 8 μM for A101 [42,46] and its final concentration of 100 μM under these reaction conditions, α for FXIIIa kinetics was determined to be 13.5 through the equation

(2) $$\alpha =1+\frac{\left[S\right]}{K_{M}}$$

The corresponding α value for TG2 inhibition kinetics was determined to be 11 based on the AL5 *K*_M_ of 10 μM [42,46] and 100 μM concentration. Apparent *K*_i_ values for inhibitor potency against FXIIIa or TG2 were taken from the reciprocal slopes of linear regressions forced through (0,1) from each normalised Dixon plot and are reported with their graphically determined standard errors.

Inhibitors deemed to be operating irreversibly were evaluated under Kitz & Wilson conditions [58,59]. Observed pseudo-first-order rate constants (*k_obs_*) for enzyme inactivation were extracted from the fluorescence-time or absorbance-time data at each inhibitor concentration through non-linear fitting using a mono-exponential decay model as in

(3) $$FA_{t}=FA_{0}+\left(FA_{plateau}-FA_{0}\right)\left(1-e^{-k_{obs}t}\right)$$

The *k_obs_* values from duplicate trials were then averaged and used to determine the first-order half-lives. All fluorescence-time and absorbance-time data sets were treated over 3 half-lives, and the observed rate constants were calculated as described. The mean and standard deviations for the observed rate constants were then plotted against the inhibitor concentrations after correction by division of the appropriate α parameter, producing hyperbolic saturation plots. For inhibitors where a clear plateau in the observed rate constant was observed at high inhibitor concentrations in the saturation plot, non-linear hyperbolic fitting was performed according to Equation (4).

(4) $$k_{obs}=\frac{k_{inact}\frac{\left[I\right]}{\alpha }}{K_{I}+\frac{\left[I\right]}{\alpha }}$$

This fitting was used to determine *k_inact_* and *K*_I_ values, along with their standard errors. These extracted values were then used in the calculation of *k_inact_*/*K*_I_, with errors carried forward appropriately. For inhibitors that did not reach an obvious saturation in the observed rate constant, a linear regression (forced through the origin) was performed on the plots of *k_obs_ vs* [I]/α using the lowest inhibitor concentrations. The ratio of *k_inact_*/*K*_I_ in these cases was taken from the slope of the linear fit and its corresponding standard error.

### 4.5. Fluorescent Labelling of FXIIIa by SDS-PAGE

To a 1.5-mL Eppendorf was added 10 µg of thrombin-activated FXIIIa (T070 Zedira GmbH, Darmstadt, Germany) as a solution in 10 µL storage buffer (50 mM TRIS, 1 mM TCEP, 150 mM NaCl pH 7.5). A 5-µL aliquot of FXIII kinetic assay buffer (111 mM TRIS, 16 mM CaCl_2_, 333 mM NaCl pH 7.5) was added to the tube and gently vortexed. The rhodamine B derivatized probe **93** (aka **KM93**), 15 µL of a 60 µM aqueous solution, was subsequently added to the tube, the tube was gently vortexed, and enzyme labelling was allowed to occur for 20 min at room temperature. A 30-µL aliquot of Bio-Rad 2X Laemmli Sample Buffer (5% β-mercaptoethanol) was added to the sample and the solution was boiled for 5 min at 100 °C to ensure denaturation. Once the sample had cooled, 30 µL was loaded into a well of a Bio-Rad Mini-PROTEAN TGX precast 4–20% acrylamide gel. A 10-µL aliquot of Bio-Rad Precision Plus Unstained Protein Standards was loaded and electrophoresis was performed at 120 V for 1 h. The gel was first visualised using a Bio-Rad ChemiDoc MP Imager for fluorescent bands (green epifluorescence illumination 605/50 nm filter). The gel was then stained with Coomassie and visualised again.

### 4.6. Synthesis of Peptidic Inhibitors, Small Molecule Inhibitors, and Fluorescent Probe

Synthetic schemes, experimental procedures, and characterization data for all intermediates and final compounds are provided in the Supplementary Materials.

### 4.7. Cellular Labelling and Microscopy of FXIIIa

#### 4.7.1. Reagents and Antibodies

MEM Alpha (αMEM) (12561-056), penicillin-streptomycin, L-glutamine, and sodium pyruvate were from Gibco (Burlington, ON, Canada). Fetal bovine serum was from Hyclone (Waltham, MA, USA). Human M-CSF (macrophage-colony stimulating factor) was from PeproTech (Rocky Hill, NJ, USA). Thiazolyl Blue Tetrazolium Bromide (MTT) was purchased from Sigma. Sheep anti-human Factor XIII-A (SAF13A-AP) was from Affinity Biologicals (Ancaster, ON, Canada). Donkey anti-sheep IgG cross-adsorbed secondary antibody AlexaFluor^®^-488 (A-11015), AlexaFluor^®^-488-phalloidin and DAPI (4′, 6-diamidino-2-phenylindole) were from Thermo Fisher Scientific (Rockford, IL, USA).

#### 4.7.2. Animals

C57BL/6 mice were purchased from Jackson Laboratory (Bar Harbor, ME, USA). Mice were housed in a pathogen-free environment and maintained under standardised conditions. All experimental protocols were approved by the Animal Care Committee of McGill University.

#### 4.7.3. Bone Marrow Macrophage Isolation Culture

Bone marrow cells were extracted from 6- to 10-week-old C57BL/6 mice and cultured for 24 h with 25 ng/mL M-CSF. Non-adherent cells were collected and plated at 5 × 10^4^ cells/cm^2^ for another 48 h in the presence of M-CSF. Medium composed of αMEM, 1% penicillin-streptomycin, 1% L-glutamine solution, 1% sodium pyruvate, and 10% FBS was used for the culture. The cells that had adhered to plates were M-CSF-dependent bone marrow-derived macrophages (BMMs). Probe **93** (aka **KM93**) was diluted in DMSO at stock concentration and added to the cell culture at the indicated concentrations.

#### 4.7.4. Protein Extraction and SDS-PAGE

Bone marrow macrophages were cultured in the presence and absence of probe **93** (aka **KM93**) and cells were collected at their experimental endpoints of 6 h and 48 h as follows: cells were washed with PBS and extracted with ice-cold cell lysis buffer containing 50 mM Tris (pH 7.5), 0.5 M NaCl, and 2% Igepal, 1% protease inhibitor cocktail, and 1% phosphatase inhibitor cocktail. Extraction was done with 30 min incubation followed by scraping, 30 s of sonication, and centrifugation at 14,000× *g* for 15 min at 4 °C. A bicinchoninic acid (BCA) Protein Assay Kit (Thermo Fisher Scientific) was used to measure protein concentration. Twenty (20) µg of proteins were loaded onto 10% SDS-polyacrylamide gels and run in a Bio-Rad electrophoresis system (Bio-Rad, Mississauga, ON, Canada). Protein bands labelled by probe **93** (aka **KM93**) were imaged with a Typhoon 8600 fluoroimager (GE Healthcare) using Ex/Em 532/610 nm and then stained with Coomassie.

#### 4.7.5. Immunofluorescence Microscopy

Bone marrow macrophages were plated onto NUNC 8-well cell culture chamber slides (Thermo Scientific) as described above. At the endpoints, cells were fixed with 3.7% formaldehyde for 10 min and then blocked with 2% bovine serum albumin (BSA) for 30 min. Cells were then incubated with probe **93** (**KM93**) for the indicated time and then washed and incubated with primary antibody (Anti-FXIII-A) for 2 h, followed by AlexaFluor^®^ conjugated secondary antibody for 1 h. F-actin was labelled with AlexaFluor^®^ 488-phalloidin and nuclei were stained with DAPI. Images were taken under 40× objective by using the Zeiss Axioscope 5 microscope (Carl Zeiss, Hamburg, Germany).

## Figures and Tables

**Table 1:** Structural classification of peptidic FXIIIa inhibitors. The linker length (n methylene units) and warhead are presented along with an alphabetical nomenclature system for noting the distances of the electrophilic carbon (E^+^ C) and carbonyl carbon (CO C) from the peptide backbone. For example, a designation of d implies that the feature is present at the δ carbon, the 3rd carbon atom away from the backbone’s a/α-carbon.

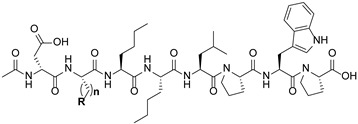				
Inh.	R	n	E^+^ C Position	CO C Position
**11**	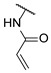	1	f	d
**12**		2	g	e
**13**		3	h	f
**14**		4	i	g
**20**	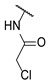	1	e	d
**21**		2	f	e
**22**		3	g	f
**23**		4	h	g
**45**	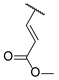	1	c	e
**46**		2	d	f
**47**		3	e	g

**Table 2:** Kinetic parameters, inhibition modes, and isozyme selectivity of acrylamide-, α-chloroacetamide, and α,β-unsaturated methyl ester-bearing peptidic inhibitors of FXIIIa inhibitors with varying linker lengths. Data were determined using distinct continuous activity assays for FXIIIa and TG2 detailed in the Materials and Methods section.

	FXIIIa				TG2		
Inh.	Inh. Mode	*k_inact_* (min^−1^)	*K*_i_ or *K*_I_ (μM)	*k_inact_*/*K*_I_ (M^−1^ min^−1^)	Inh. Mode	*k_inact_*/*K*_I_ (M^−1^ min^−1^)	Selectivity
11	None *	NA	NA	NA	None **	NA	NA
12	Rev	NA	193 ± 20	NA	None **	NA	FXIIIa
13	Rev	NA	135 ± 16	NA	None **	NA	FXIIIa
14	Rev	NA	125 ± 20	NA	None **	NA	FXIIIa
20	None *	NA	NA	NA	Irrev	1962 ± 82	TG2
21	Irrev	-	-	7422 ± 525	None **	NA	FXIIIa
22	Irrev	0.0988 ± 0.0185	7.1 ± 3.1	13,922 ± 6664	Irrev	2922 ± 260	2.6 FXIIIa
23	Irrev	-	-	4907 ± 325	Irrev	3565 ± 229	1.4 FXIIIa
45	Rev	NA	53 ± 12	NA	None **	NA	FXIIIa
46	Irrev	0.1765 ± 0.0160	0.4732 ± 0.0847	372,992 ± 74,839	Irrev	56,520 ± 2044	6.6 FXIIIa
47	Rev	NA	0.0941 ± 0.0105	NA	Irrev	31,780 ± 2218	Irrev TG2

* = no inhibition detected up to 200 Μm; ** = no inhibition detected up to 350 μM.

**Table 3:** Kinetic parameters and isozyme selectivity of small molecule FXIIIa inhibitors with various N-terminal acidic groups, C-terminal hydrophobic units, and central warhead amino acid residues. Data were determined using distinct continuous activity assays for FXIIIa and TG2 detailed in the Materials and Methods section.

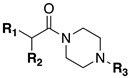					
Inh.	R_1_	R_2_	R_3_	FXIIIa *K*_i_ (µM)	TG2 *K*_I_ (µM)
**71**	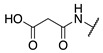	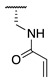	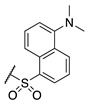	Interference *	>360
**79**	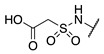	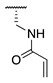	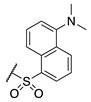	Interference *	>360
**75**	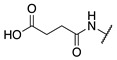	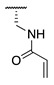	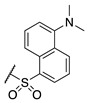	Interference *	>360
**74**	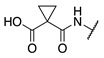	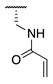	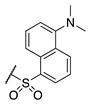	Interference *	>360
**73**	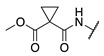	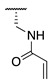	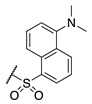	Interference *	>360
**76**	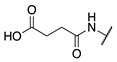	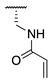	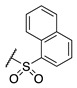	Interference *	>360
**72**	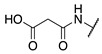	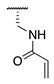	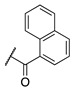	249.1 ± 69.3	>360
**77**	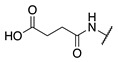	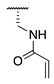	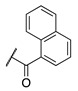	69.0 ± 4.1	>360
**84**	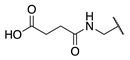	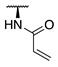	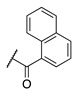	107.6 ± 19.1	>360
**87**	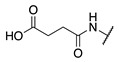	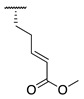	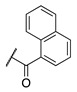	131.1 ± 7.9	14.3 ± 7.0
**88**	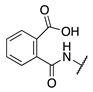	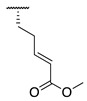	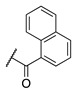	265.0 ± 46.8	21.2 ± 9.0

* Data afflicted by fluorescence interference.

## Data Availability

Data will be provided upon reasonable request.

## Supplementary Materials

The supporting information containing detailed description of the synthesis [42,51,53,65,66,67,68,69,70,71,72,73,74,75,76,77], characterization, and supplementary figures can be downloaded at: https://www.mdpi.com/article/10.3390/molecules28041634/s1, Scheme S1: Synthesis of Fmoc-AA(Acrylamide)-OH monomers 5-8 required for the production of acrylamide-bearing peptidic inhibitors; Scheme S2: Synthesis of Fmoc-D-Asp(OtBu)-OH 9 required for the production of acrylamideand α-chloroacetamide-bearing peptidic inhibitors; Scheme S3: Synthesis of acrylamide-bearing peptidic inhibitors 11–14; Scheme S4: Synthesis of Fmoc-AA(Alloc)-OH monomers 17–19 required for the production of αchloroacetamide-bearing peptidic inhibitors; Scheme S5: Synthesis of α-chloroacetamide-bearing peptidic inhibitors 20–23; Scheme S6: Synthesis of Boc-Glu(MA)-OH 28 required for the production of ZED1301; Scheme S7: Synthesis of Boc-AA(MA)-OH monomers 33 and 37 required for the production of α,β-unsaturated ester-bearing peptidic inhibitors; Scheme S8: Synthesis of Ac-D-Asp(OtBu)-OH 38 required for the production of α,β-unsaturated ester-bearing peptidic inhibitors; Scheme S9: Synthesis of α,β-unsaturated ester-bearing peptidic inhibitors 45–47; Scheme S10: General synthetic scheme to arrive at L-Dap key intermediate for small molecule inhibitors 66–68; Scheme S11: Synthetic scheme to arrive at malonyl inhibitors 71 and 72; Scheme S12: Synthetic scheme to arrive at cyclopropyl inhibitor 74 through the methyl ester 73; Scheme S13: Synthetic scheme to generate succinyl inhibitors 75–77; Scheme S14: Synthetic scheme to synthesize sulfonyl inhibitor 79; Scheme S15: General synthetic scheme to arrive at D-Dap scaffold key intermediate 83; Scheme S16: Synthetic scheme to generate D-Dap succinyl inhibitor 84; Scheme S17: General synthetic scheme to generate α,β-unsaturated warhead key intermediate 86; Scheme S18: Synthetic scheme to produce α,β-unsaturated succinyl inhibitor 87; Scheme S19: Synthetic scheme to produce α,β-unsaturated phthalyl inhibitor 88; Scheme S20: Synthesis of Rhodamine-B-ZED1301 fluorescent probe 93 (KM93); Scheme S21: Synthesis of Fmoc-6AH-OH 89 required for the production of fluorescent probe 93 (KM93); Figure S1: Fluorescence-time curve of the spike experiment performed with inhibitor 47 to confirm the reversibility of FXIIIa inhibition. Additional A101 substrate was added to the reactions corresponding to the positive control with no inhibitor (+ve), the negative control with no enzyme (−ve), and all 4 inhibitor concentrations (1, 2, 5, and 10 μM) after the initial plateaus in fluorescence had been reached. Fluorescence emission (RFU, relative fluorescence unit emission at 418 nm after excitation at 313 nm) was then monitored over time; Figure S2: Fluorescence-time curve of the spike experiment performed with inhibitor 23 to confirm the irreversibility of FXIIIa inhibition. Additional A101 substrate was added to the reactions corresponding to the positive control with no inhibitor (+ve) and the highest inhibitor concentration (200 μM) after the initial plateaus in fluorescence had been reached. Fluorescence emission (RFU, relative fluorescence unit emission at 418 nm after excitation at 313 nm) was then monitored over time; Figure S3: Substrate spike experiment with small molecule inhibitor 79 and FXIIIa. Upon completion of the activity assay, another 100 µM of A101 was added and the RFU increased, implying FXIIIa was still active (top and bottom left panels); Figure S4: Inhibitor spike experiment with small molecule inhibitor 79. Upon completion of the activity assay another dose of 320 µM 79 was added and the RFU plateau at a lower value (top right panel); Figure S5: Inhibition experiment of FXIIIa with small molecule inhibitor scaffold components dansyl amide and acrylamide assayed at four different concentrations (0, 100, 200, 320 µM); Figure S6: SDS-PAGE of commercially available FXIIIa incubated with 30 µM fluorescent probe 93 (KM93) visualized first for fluorescence and then using Coomassie Blue staining; Figure S7: Full SDS-PAGE gel of fluorescent labelling of commercially available FXIIIa with 30 µM 93 (KM93). Note the excess labelling agent at the very bottom edge of the gel does not correspond to protein; Figure S8: (A) Fluorescent labelling of FXIIIa in murine bone marrow macrophages (BMM). BMMs were incubated with 0–20 µM KM93 for 6 h (top gel) or for 48 h (bottom gel), lysed, and protein extracts were prepared and resolved with denaturing, 10% SDS-PAGE. The gels were visualized using a fluoroimager (Ex/Em 562/610). The gels show a dose dependent labelling of a ~80 kDa band, which corresponds to a molecular weight of FXIIIa, consistent with the red fluorescence of the rhodamine moiety of KM93. The identity of the band at ~100 kDa is unknown but it is also present in the negative control and thus represents background. (B) Coomassie Blue stained gels confirm equal loading in each lane; Figure S9: Fluorescence visualization of KM93 in murine bone marrow macrophages (BMM). Cells were incubated with 20 µM KM93 for 48 h in microscopy chamber slides. At end point, cells were fixed, cytoskeletal actin was stained with AlexaFluor^®^-488-phalloidin (green) and nuclei were stained with DAPI (blue). KM93 was visualized in the 568-nm channel (red). (A): Cells that were incubated in the absence of the probe showed no signal in the 568-nm channel (negative control). (B): Strong red fluorescence was seen observed after 48 h incubation with 20 µM KM93. The probe was confirmed to be intracellular by actin staining (see inset C). White magnification bar represents 20 µm; Figure S10: FXIIIa inhibitor ZED1301 is able to block labelling by KM93 probe in BMM cells. BMM cells were preincubated in the absence or presence of 20 µM ZED1301 for 2 hours to inhibit FXIIIa, prior to addition of 20 µM KM93 and further incubation for 4 h. Cells were fixed and washed, nuclei were stained with DAPI (blue) and incorporation of the red probe KM93 was observed by fluorescence microscopy. Negligible red fluorescence was observed in cells blocked by pre-incubation with ZED1301. Magnification bar equals 40 µm.
