# Supplementary material for: Peptidic Inhibitors and a Fluorescent Probe for the Selective Inhibition and Labelling of Factor XIIIa Transglutaminase
Source: Molecules. 2023 Feb 8;28(4):1634. doi: 10.3390/molecules28041634 (PMC9960274; doi:10.3390/molecules28041634)
Supplement: Supplementary file 1 [file molecules-28-01634-s001.zip › molecules-2147293-supplementary.pdf]

Supplementary Material for:

Peptidic Inhibitors and Fluorescent Probe for the Selective Inhibition and Labelling of Factor XIIIa Transglutaminase

Eric W. J. Gates[†,1], *Kian Mansour*[†,1], *Sahar Ebrahimi Samani*[2], *Sammir Shad*[1], *Mari T. Kaartinen*[2,3], *and Jeffrey W. Keillor*[1,*]

[1]Department of Chemistry and Biomolecular Sciences, University of Ottawa, Ottawa, Ontario K1N 6N5, Canada

[2]Faculty of Medicine (Division of Experimental Medicine), McGill University, Montréal, Québec, H3A 0C7, Canada

[3]Faculty of Dental Medicine and Oral Health Sciences, McGill University, Montréal, Québec, H3A 0C7, Canada

*****Correspondence: jkeillor@uottawa.ca

[†]These authors contributed equally to this work.

Table of Contents:

Synthesis of Intermediates and Final Inhibitors.. 3
Experimental Data for Intermediates and Final Inhibitors 14
Supplementary Kinetic Traces and Inhibition Experiments 37
SDS-PAGE of Fluorescent Labelling Experiment 40
Supplementary Cellular Labelling Figures 41
Kinetic Fitting for Inhibitors 44
HPLC Traces of Peptidic Inhibitors 51
References 57

Synthesis of Intermediates and Final Inhibitors

For the synthesis of the acrylamide-bearing inhibitors **11-14**, a direct approach involving semi-automated Fmoc-based solid-phase peptide synthesis (SPPS) was envisioned as shown in Scheme S3. Loading 2-chlorotrityl chloride (CTC) resin with Fmoc-Proline to form **10** could be followed by cycles of amide couplings and Fmoc-deprotections to assemble the desired linear octapeptides. The final inhibitors **11-14** would then be isolable after N-terminal acetylation, cleavage off resin, and global deprotection of the sidechain protecting groups. In order to carry out this synthesis, unnatural amino acid (AA) monomers bearing acrylamide functionalities on their sidechain amines with different linker lengths were required, and were synthesized accordingly. For ease of introduction into SPPS, the acrylamide-bearing monomers were assembled in the form of Fmoc-AA(Acrylamide)-OH compounds **5-8** as shown in Scheme S1. Commercially available Boc-AA-OH compounds were first treated with acryloyl chloride to form Boc-AA(Acrylamide)-OH compounds **1-4**. Deprotection of the Boc group with TFA followed by reprotection with Fmoc-chloride prepared the desired monomers **5-8** for convenient incorporation into the peptidic inhibitors. The final unnatural amino acid required for SPPS of the acrylamide-bearing inhibitors, namely Fmoc-D-Asp(OtBu)-OH **9**, was prepared from commercially-available H-D-Asp(OtBu)-OH using Fmoc-chloride as shown in Scheme S2. The acrylamide-bearing monomers **5-8**, along with the D-Asp derivative **9**, were then carried into the SPPS outlined in Scheme S3, allowing for the production of the desired acrylamide-bearing peptidic inhibitors **11-14** in sufficient quantities for characterization and subsequent kinetic evaluation with FXIIIa.

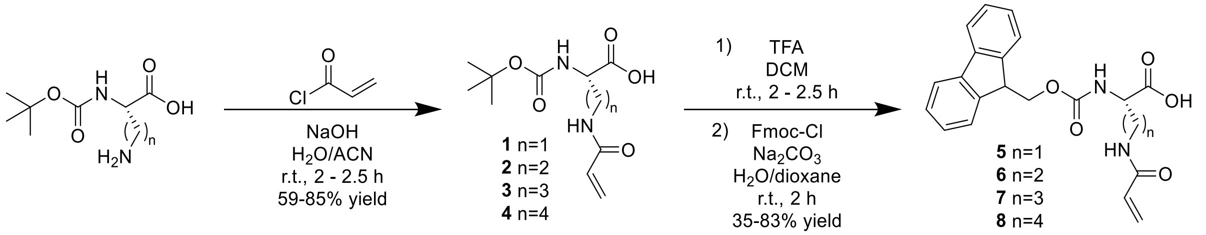

Scheme S1. *Synthesis of Fmoc-AA(Acrylamide)-OH monomers **5-8** required for the production of acrylamide-bearing peptidic inhibitors.*

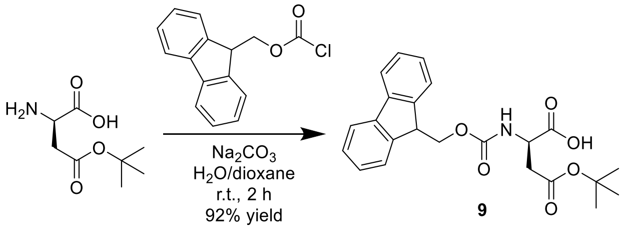

Scheme S2. *Synthesis of Fmoc-D-Asp(OtBu)-OH **9** required for the production of acrylamide- and α-chloroacetamide-bearing peptidic inhibitors.*

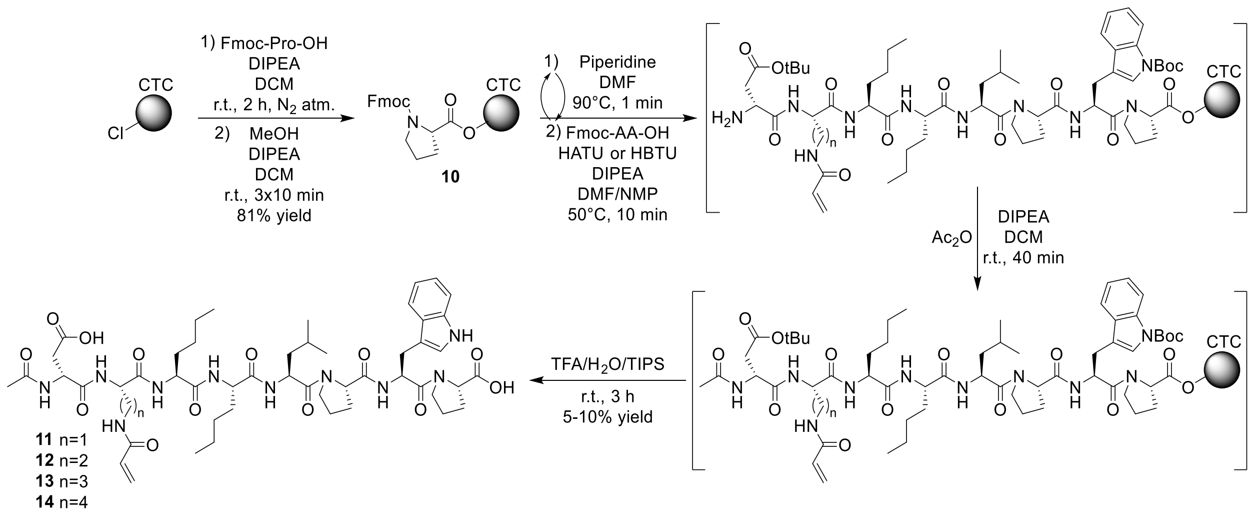

Scheme S3. *Synthesis of acrylamide-bearing peptidic inhibitors **11-14**.*

Next, the synthesis of the α-chloroacetamide-bearing inhibitors **20**-**23** was addressed. Due to its high electrophilicity, the α-chloroacetamide functionality was anticipated to be unstable in the presence of high concentrations of piperidine, used in standard SPPS Fmoc deprotections. To avoid this potential issue and prevent exposing the α-chloroacetamide to any piperidine, the warhead would have to be installed onto the peptide after the linear chain had been assembled fully. Rather than introducing the warhead-bearing amino acid as the unnatural monomer already bearing the electrophile, as was performed with the synthesis of the acrylamides in Scheme S1, this residue could be added to the chain while carrying a sidechain amine masked with a protecting group which could later be removed and replaced with the desired α-chloroacetamide moiety. The Alloc protecting group was selected for this purpose due to its orthogonality to the Fmoc, Boc, and *tert*-butyl ester removal conditions. To prepare the required amino acid monomers of the form Fmoc-AA(Alloc)-OH with varying linker lengths, compounds **17**-**19**, standard protecting group manipulations were performed from commercially available starting materials, as shown in Scheme S4. The required amino acid with the longest linker length, namely Fmoc-Lys(Alloc)-OH, was commercially-available. The Alloc-bearing monomers and the D-Asp derivative **9** were carried into the SPPS as described in Scheme S5. Linear chain completion followed by N-terminal acetylation, palladium-catalyzed Alloc-deprotection, warhead attachment with chloroacetic anhydride, and cleavage and global deprotection furnished the α-chloroacetamide-bearing inhibitors **20**-**23** in adequate yield and purity.

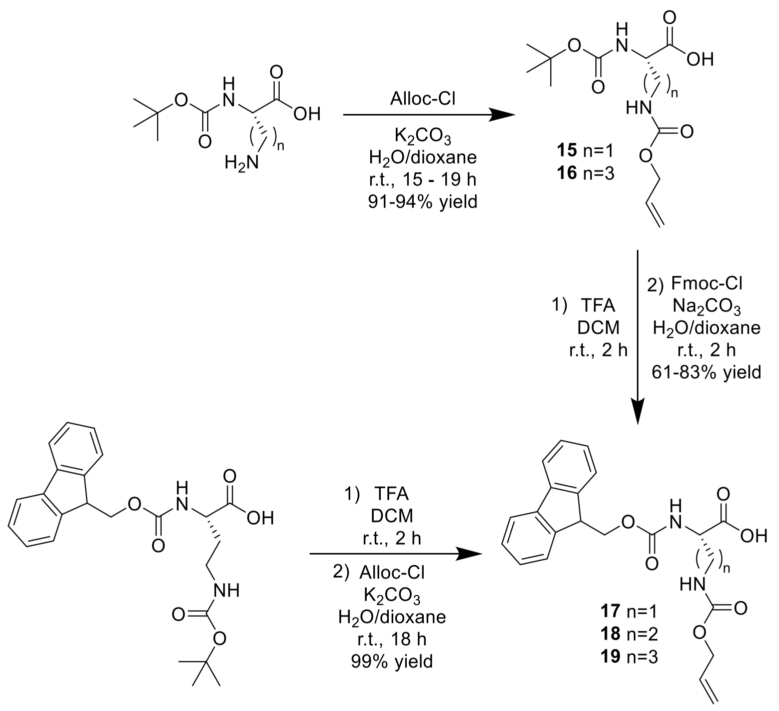

***Scheme S4.** Synthesis of Fmoc-AA(Alloc)-OH monomers **17-19** required for the production of α-chloroacetamide-bearing peptidic inhibitors.*

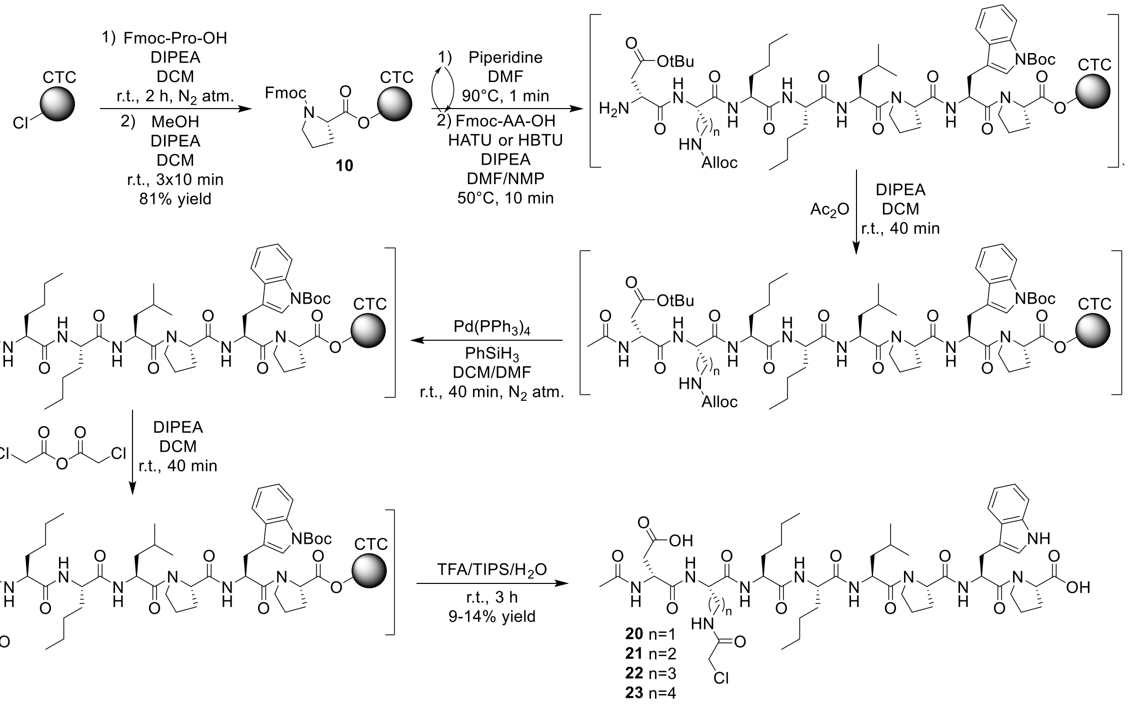

***Scheme S5.** Synthesis of α-chloroacetamide-bearing peptidic inhibitors **20-23**.*

The peptidic inhibitors **45-47**, bearing the unsaturated ester warhead, were synthesized through a route similar to that reported by Zedira in the original ZED1301 synthesis [51,53]. The required amino acids of the form Boc-AA(MA)-OH **28**, **33**, and **37** were prepared as shown in

Schemes S6 & S7. For the two-methylene-linker Boc-Glu(MA)-OH **28**, leading to ZED1301, a commercially-available glutamate derivative was first methylated on its sidechain to produce **24** (Scheme S6), and was subsequently di-Boc protected at the α-amine to yield **25**. This di-Boc protection masks the acidic proton which is believed to interfere with the subsequent DIBAL-mediated reduction to aldehyde **26** [65–67], which was subjected to a Wittig reaction to produce the desired unsaturated ester warhead in **27**. Protecting group manipulation then led to the formation of mono-Boc derivative **28**. For longer and shorter linker derivatives of **28**, outlined in Scheme S7, an aspartate derivative was chosen as the starting point. A Steglich esterification of the C-terminus produced compound **29**, which was in perfect shape to proceed through the analogous sequence of protection, DIBAL reduction, Wittig installation of the unsaturated ester, and protecting group manipulation, as described previously, to produce the desired Boc-Asp(MA)-OH compound **33**. To obtain the amino acid with one methylene longer than the glutamate derivative, namely the homoglutamate (Hmg) position unsaturation, the aspartate-derived compound **32** was carried forward, as this already possesses the necessary protecting groups and a carbonyl placed at the ε carbon. Hydrogenation of the unsaturation led to **34**, which after the same sequence of transformations cleanly produced the Boc-Hmg(MA)-OH compound **37**. The final amino acid required for these peptides, Ac-D-Asp(OtBu)-OH **38**, was produced from H-D-Asp(OtBu)-OH through a reported protocol, as shown in Scheme S8 [68]. With all the required amino acids in hand, the peptide synthesis was carried out as shown in Scheme S9, again in a manner similar to that reported for the ZED1301 synthesis by Zedira [51]. Linear chain extension was terminated after the attachment of the warhead-bearing residue. Deprotection of the N-terminal Boc group with TFA resulted in simultaneous deprotection of the Trp residue's Boc group and cleavage from the resin. The resultant peptides **39-41** were subsequently coupled in solution with the final residue **38**, furnishing penultimate intermediates **42-44**, which could easily be turned into the desired inhibitors **45-47** through *t*-butyl ester deprotection in acid. This synthetic route, developed by Zedira, avoids exposing the electrophilic unsaturated ester moiety to any nucleophilic piperidine.

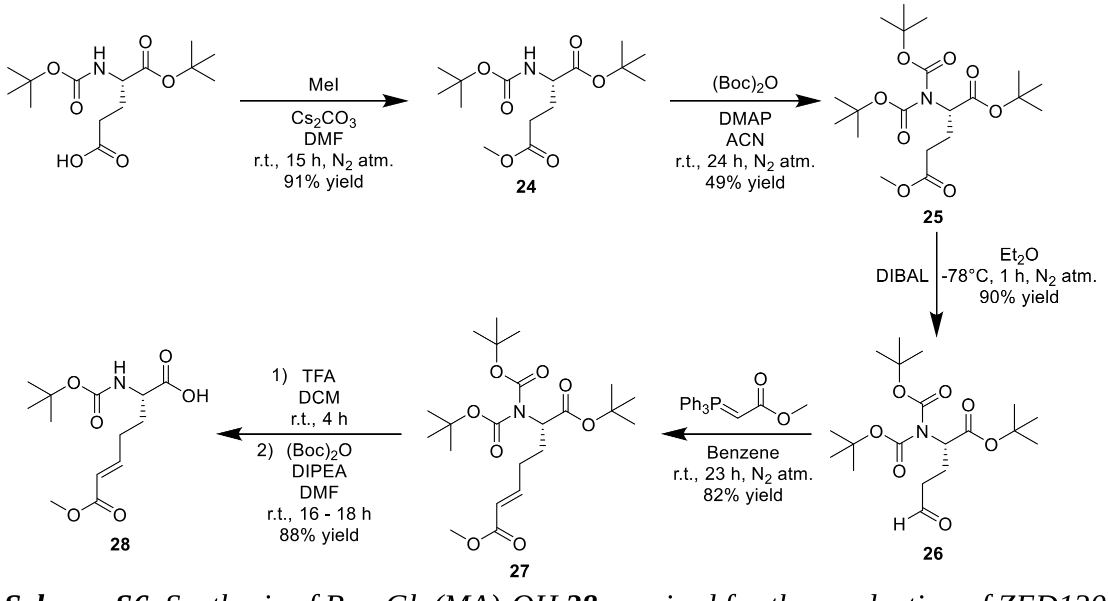

Scheme S6. *Synthesis of Boc-Glu(MA)-OH **28** required for the production of ZED1301* [53].

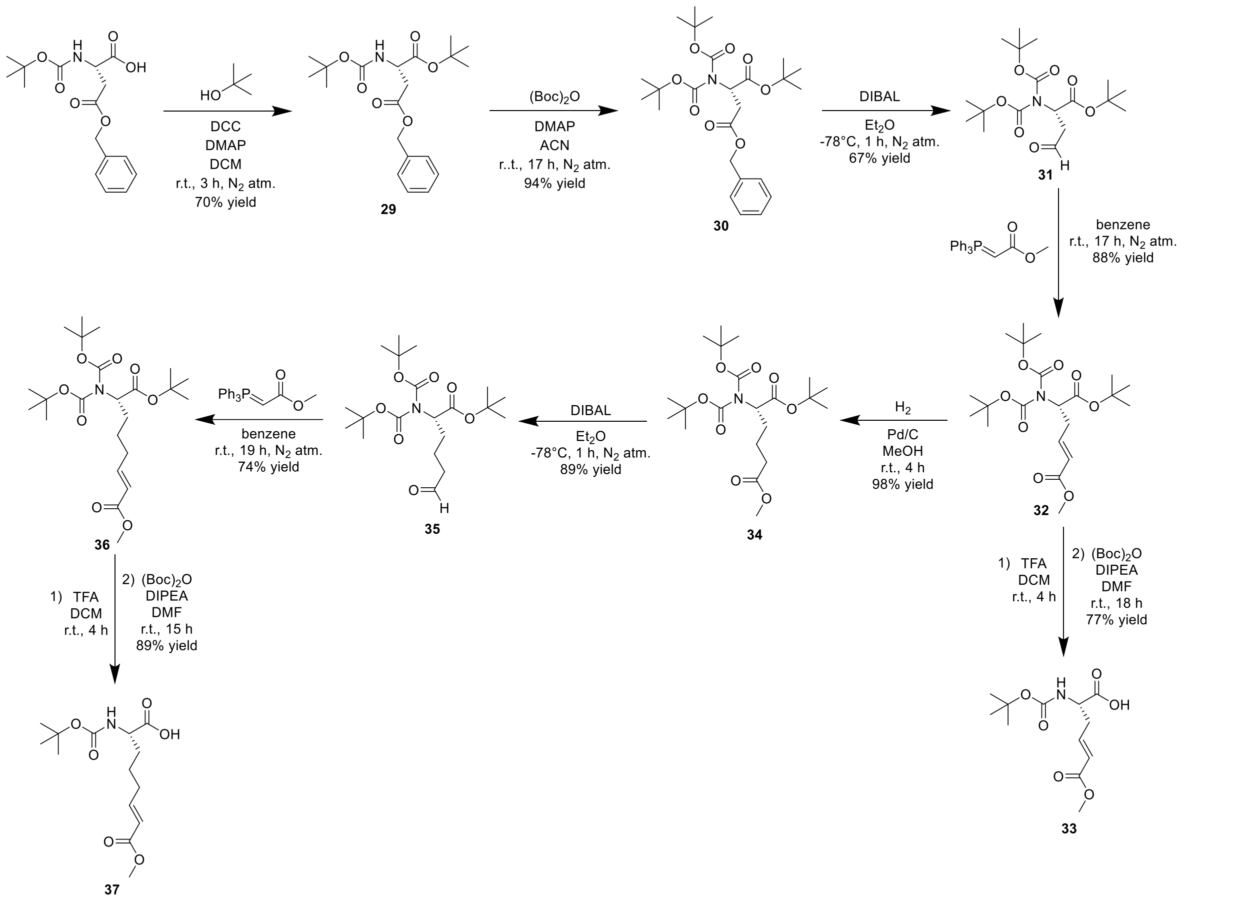

Scheme S7. *Synthesis of Boc-AA(MA)-OH monomers* ***33*** *and* ***37*** *required for the production of α,β-unsaturated ester-bearing peptidic inhibitors.*

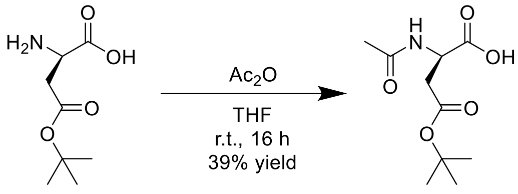

38

Scheme S8. *Synthesis of Ac-*D*-Asp(OtBu)-OH* ***38*** *required for the production of α,β-unsaturated ester-bearing peptidic inhibitors* [68].

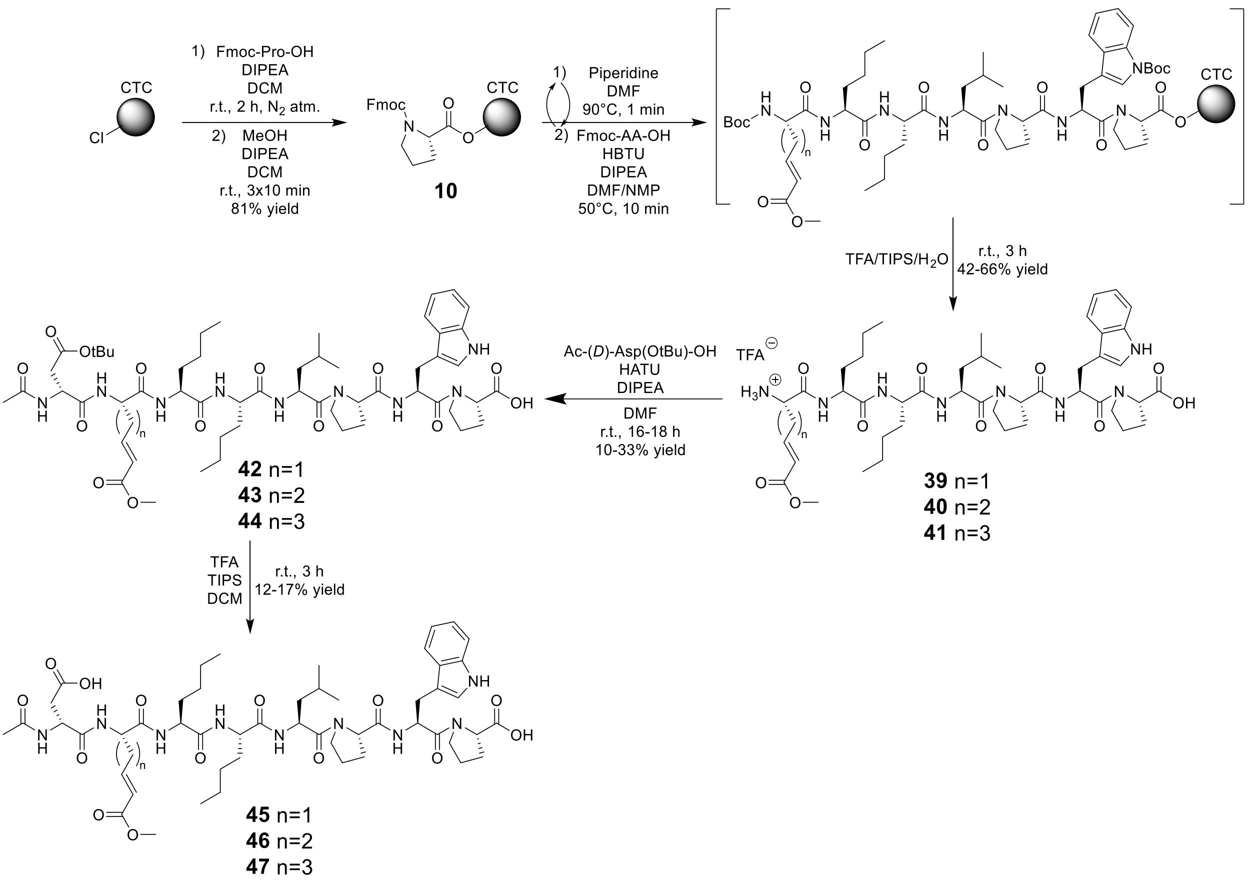

Scheme S9. *Synthesis of α,β-unsaturated ester-bearing peptidic inhibitors* ***45-47*** [51].

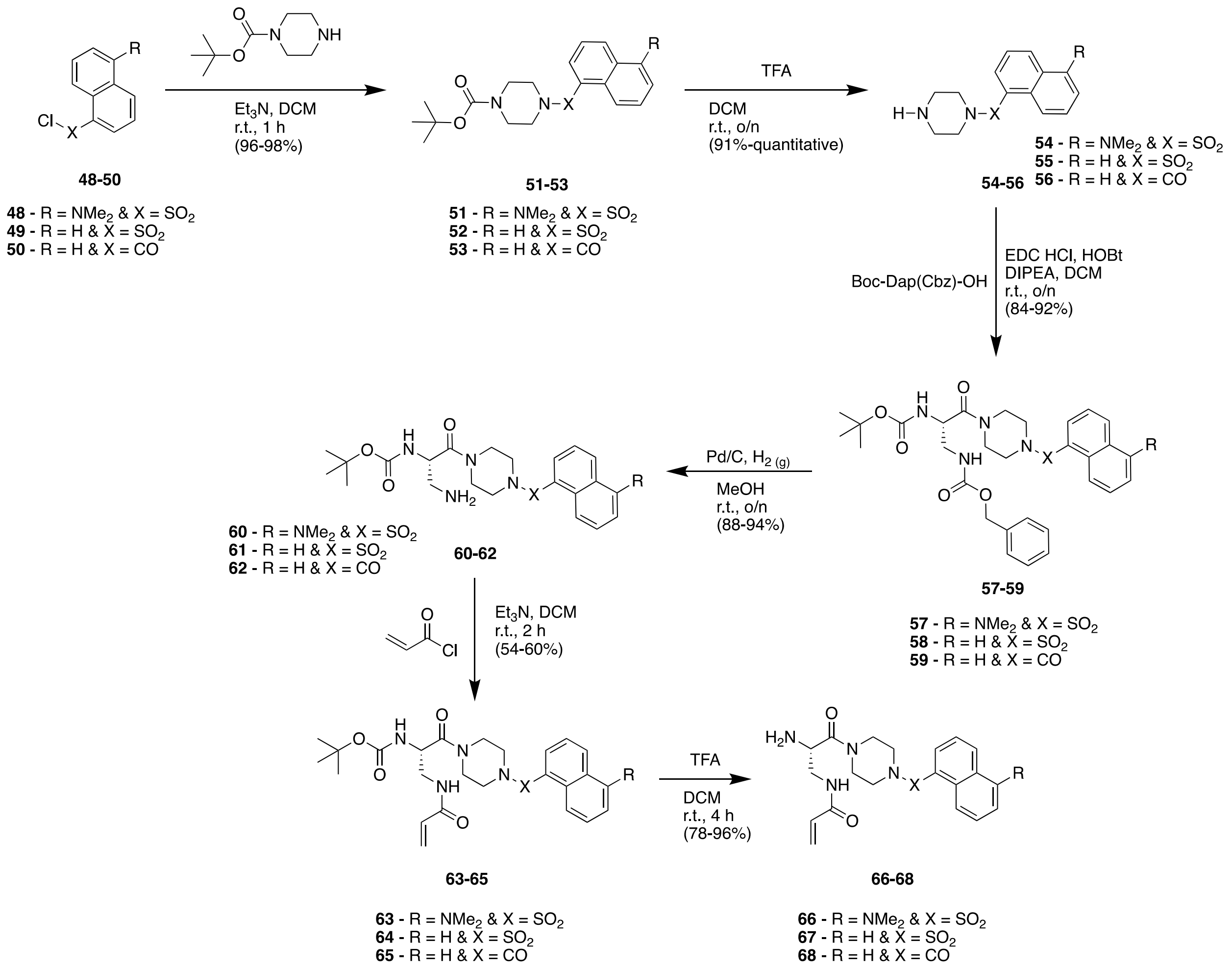

Scheme S10. *General synthetic scheme to arrive at* L*-Dap key intermediate for small molecule inhibitors* ***66-68****.*

The small molecule inhibitors of FXIIIa were synthesized by first generating a key intermediate which would allow for functionalization at the N-terminus with various carboxylic acids (Scheme S10). Starting from commercially available acyl chloride or sulfonyl chlorides **48-50**, an acylation was performed with N-Boc-Piperazine. The Boc group was then cleaved under acidic conditions with TFA to generate the free amines **54-56**. An amide coupling was then performed with EDC, HOBt, and the corresponding Boc-L-Dap(Z)-OH to gain access to the fully protected **57-59**. Hydrogenolysis with catalytic palladium on carbon liberated the β-amine **60-62**. An acrylation with acryloyl chloride successfully installed the acrylamide warhead to generate the protected key intermediates **63-65**. A final Boc deprotection with TFA yielded the key intermediates **66-68** to allow for functionalization at the N-terminus.

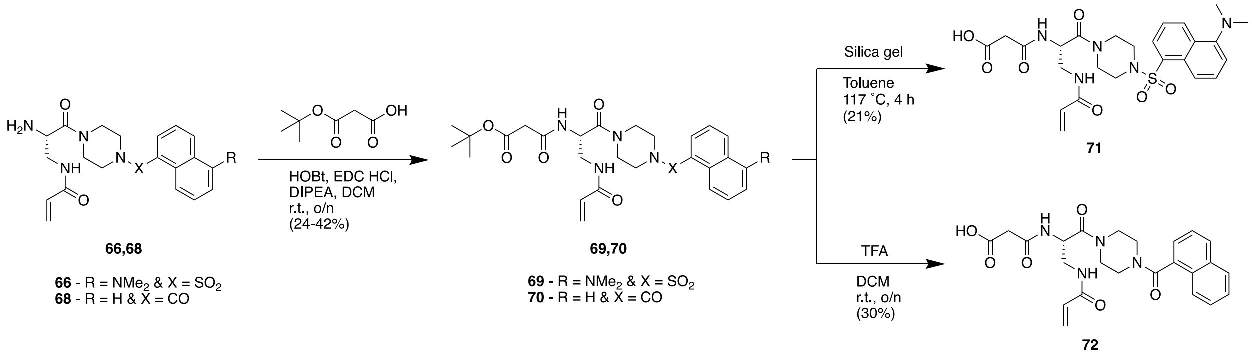

Scheme S11. *Synthetic scheme to arrive at malonyl inhibitors **71** and **72**.*

Malonyl inhibitors **71** and **72** were synthesized by an amide bond coupling with mono-*t*-butyl malonate and subsequent acidic cleavage of the *t*-butyl ester to yield the final inhibitors (Scheme S11).

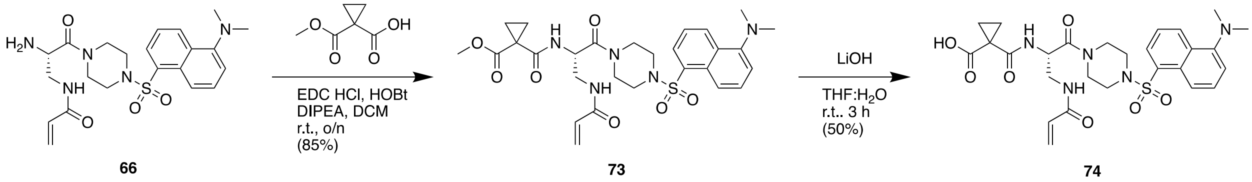

Scheme S12. *Synthetic scheme to arrive at cyclopropyl inhibitor **74** through the methyl ester **73**.*

To generate the cyclopropyl inhibitor **74**, a coupling with the mono-methyl ester protected diacid was accomplished with EDC and HOBt. The ester was then hydrolyzed in aqueous conditions with lithium hydroxide to produce the inhibitor **74** (Scheme S12).

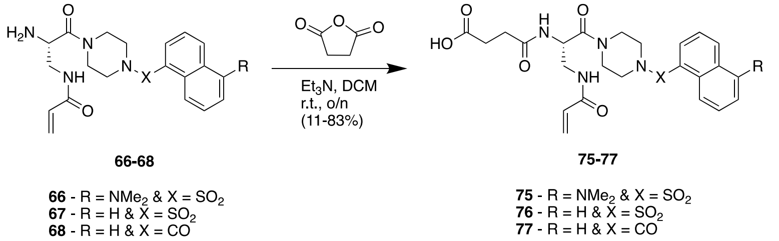

Scheme S13. *Synthetic scheme to generate succinyl inhibitors **75-77**.*

The three succinyl inhibitors were synthesized by a simple anhydride opening with succinic anhydride. Under basic conditions the key intermediates were exposed to succinic anhydride and yielded inhibitors **75-77** (Scheme S13).

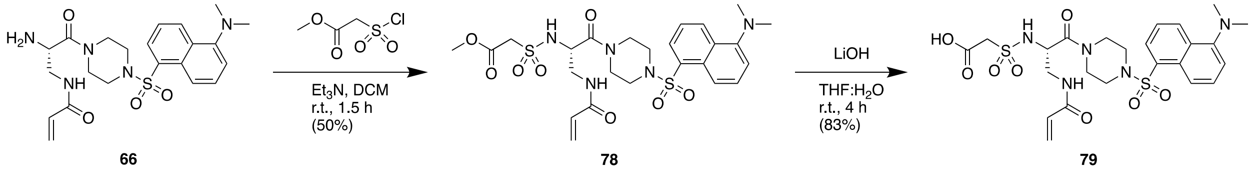

Scheme S14. *Synthetic scheme to synthesize sulfonyl inhibitor **79**.*

The dansyl sulfonamide inhibitor was generated from a sulfonamide coupling with the key intermediate **66** and chlorosulfonyl-acetic acid methyl ester. A methyl ester hydrolysis then deprotected the carboxylate to produce **79** (Scheme S14).

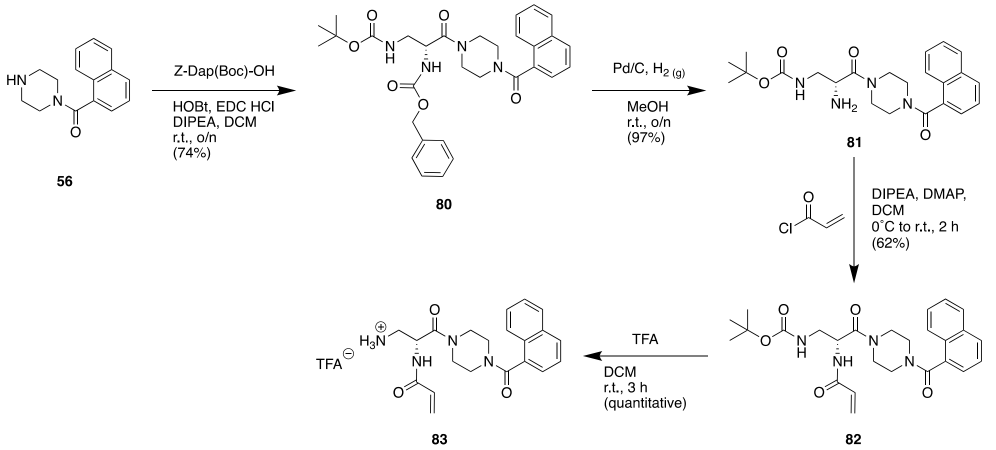

Scheme S15. *General synthetic scheme to arrive at* D*-Dap scaffold key intermediate* ***83****.*

To probe the effect of the decreased linker length to the warhead, a D-Dap scaffold was synthesized through generation of a key intermediate **83**, analogous to the L-Dap scaffold. Starting from the earlier synthesized piperazine-naphthoyl **56**, an amide coupling was performed with Z-D-Dap(Boc)-OH to produce the orthogonally protected **80**. Subsequent hydrogenolysis liberated the free warhead α-amine **81**. An acrylation with acryloyl chloride provided the acrylamide intermediate **82**. A final Boc deprotection with TFA yielded the D-Dap scaffold key intermediate **83** as a TFA salt (Scheme S15).

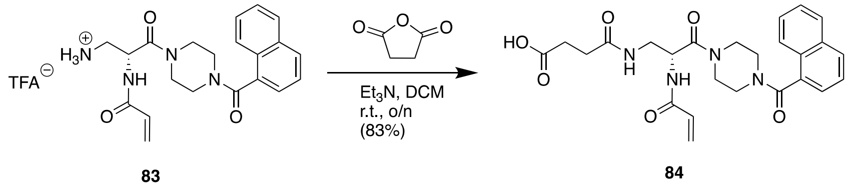

Scheme S16. *Synthetic scheme to generate* D*-Dap succinyl inhibitor* ***84****.*

The D-Dap succinyl inhibitor was synthesized from the TFA salt of key intermediate **83** and succinic anhydride under basic conditions. The carboxylic acid inhibitor **84** was obtained in 83% yield (Scheme S16).

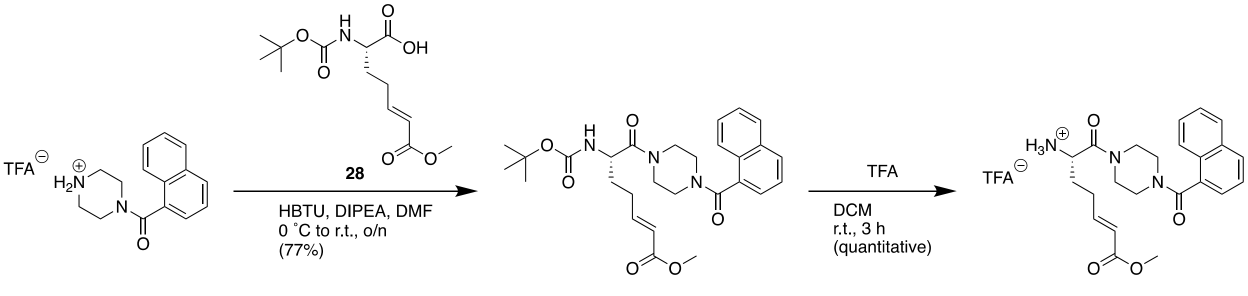

56* 85 86

***Scheme S17.** General synthetic scheme to generate α,β-unsaturated warhead key intermediate **86**.*

Using the α,β-unsaturated ester residue **28** an amide coupling tethered it to the TFA salt of piperazine-naphthoyl **56***. A Boc deprotection of the N-terminal amine formed the α,β-unsaturated warhead key intermediate **86** (Scheme S17).

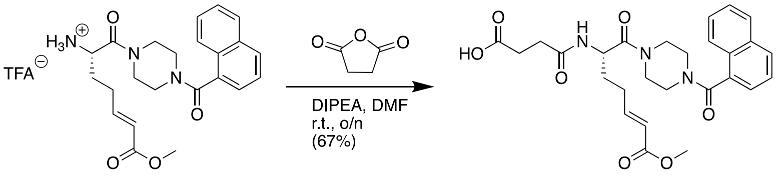

86 87

***Scheme S18.** Synthetic scheme to produce α,β-unsaturated succinyl inhibitor **87**.*

The succinyl derivative of the α,β-unsaturated small molecule library was generated from succinic anhydride under basic conditions. The α,β-unsaturated succinyl inhibitor **87** was obtained in 67% yield (Scheme S18).

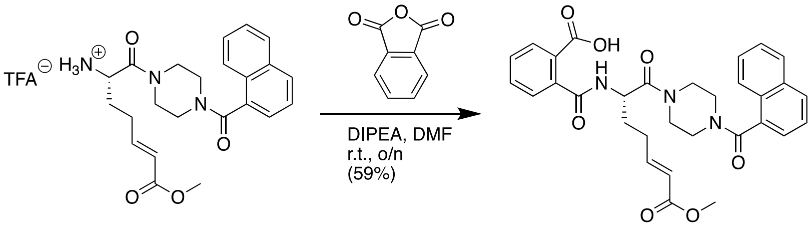

86 88

***Scheme S19.** Synthetic scheme to produce α,β-unsaturated phthalyl inhibitor **88**.*

The α,β-unsaturated phthalyl inhibitor featuring the naphthoyl hydrophobic unit was synthesized using the phthalic anhydride. Under basic conditions, the α,β-unsaturated key intermediate opened the anhydride to yield inhibitor **88** (Scheme S19).

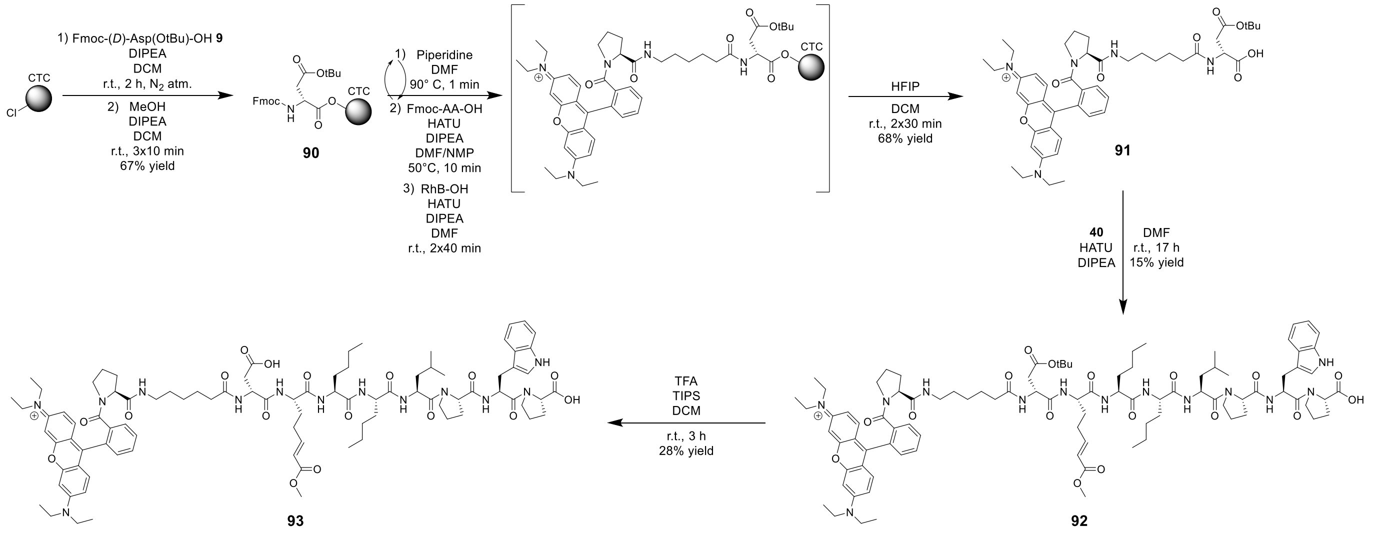

Scheme S20. *Synthesis of Rhodamine-B-ZED1301 fluorescent probe* ***93 (KM93)****.*

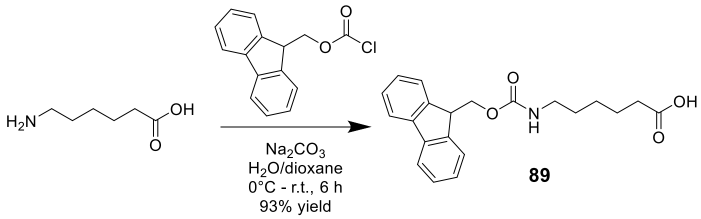

Scheme S21. *Synthesis of Fmoc-6AH-OH* ***89*** *required for the production of fluorescent probe* ***93 (KM93)****.*

The Rhodamine-B-tethered ZED1301-derived probe **93** (aka **KM93**) (Scheme S20) was synthesized in a manner similar to ZED1301. The key retrosynthetic bond disconnection made was between the warhead-bearing residue's α-amine and the adjacent D-Asp residue's carbonyl, a bond that is formed through the in-solution coupling in ZED1301 synthesis (Scheme S9). This disconnection leads to two fragments, the first of which is simply compound **40**, prepared previously (Scheme S9) as part of ZED1301. The other fragment is compound **91**, which was synthesized through solid-phase peptide synthesis as shown in Scheme S20. Fmoc-D-Asp(OtBu)-OH **9**, prepared previously in Scheme S2, was first loaded onto chlorotrityl resin, and was subsequently coupled to Fmoc-6-aminohexanoic acid (**89**, prepared in Scheme S21), followed by proline and lastly rhodamine B. Soft cleavage off resin with HFIP produced the peptide **92** in solution while retaining the *tert*-butyl ester protecting group on the aspartate sidechain which was necessary to retain selectivity in the subsequent in-solution coupling with **40** to produce **92**. This was then treated with TFA, yielding the fluorescent probe **93**.

Experimental Data for Intermediates and Final Inhibitors

General Comments

All reagents were obtained from chemical suppliers and used without further purification. All NMR spectra (^{1}H and ^{13}C) were recorded on Bruker 300, 400, or 600 MHz instruments, and chemical shifts are reported in ppm after referencing to the appropriate deuterated solvent peak [69]. High-resolution mass spectra (HRMS) were recorded at the John L. Holmes Mass Spectrometry Facility (University of Ottawa) on a quadrupole time-of-flight (QTOF) analyzer equipped with electrospray ionization (ESI). Automated peptide syntheses were performed on a Liberty Blue™ Automated Microwave Peptide Synthesizer. All solvents, reagents, and starting materials which do not have their synthesis described herein were purchased from commercial suppliers and used without further purification or characterization. Crude peptides were characterized by liquid chromatography mass spectrometry (LC-MS: Shimadzu LC-MS-2020 system; Agilent Eclipse XOB-C18 5.0-µm, 4.6 × 150-mm column or Bischoff Chromatography ProntoSIL 5.0-µm, 4.0 × 125-mm column; ACN/H_2O with 0.05% v/v formic acid, 5-95% gradient, 15 min runs, 1 mL/min; UV detection at 220 and 254 nm; ESI in positive mode, quadrupole mass analysis) for confirmation of their identities. Peptides were purified through semi-preparative high-performance liquid chromatography (HPLC: Gilson HPLC system; Kinetex XB-C18 5.0-µm, 100 Å, 10 × 150-mm column; ACN/H_2O with 0.1% v/v TFA, 10-95% gradient, 45 min runs, 3 mL/min; UV detection at 220 and 254 nm). All final peptidic inhibitors were judged to be at least 95% pure through analytical HPLC (Gilson HPLC system, Kinetex XB-C18 5.0-µm, 100-Å, 4.6 × 150-mm column; ACN/H_2O with 0.1% v/v TFA, 5-95% gradient, 20 min runs, 1 mL/min; UV detection at 220 and 254 nm). Small molecule inhibitors were purified via flash chromatography using either 230-400 mesh silica gel and organic solvent or SiliCycle C_{18} 40-63 µm silica and water/methanol eluent. Thin layer chromatography (TLC) was performed using SiliCycle aluminum backed TLC plates of 200 µm thickness and F-254 indicator.

General Procedure A: Acryloyl Attachment to Amino Acid Sidechain Amines

This protocol was adapted from that reported by Cai & Guengerich in 1999 [70]. A 1:1 mixture of acetonitrile and 1 M $NaOH_{(aq)}$ was prepared and stirred at room temperature. The appropriate commercially available Boc-AA-OH compound (1.0 eq, [Boc-AA-OH] = 0.2 M) was then added in 1 portion, followed by the dropwise addition of acryloyl chloride (1.2 eq). The reaction was allowed to stir continuously at room temperature until completion by TLC analysis (2 - 2.5 h) before rotary evaporation to remove acetonitrile. The remaining solution was then acidified to pH 1-2 through the addition of 1 M $HCl_{(aq)}$, and was subsequently extracted thrice into EtOAc. The combined organics were dried over $MgSO_4$, filtered, and concentrated by rotary evaporation. Flash column chromatography using 1.5% MeOH, 0.1% AcOH, 98.5% EtOAc as the eluent produced pure products. Residual acetic acid was removed through azeotropic rotary evaporation with toluene and concentration *in vacuo*.

General Procedure B: Deprotection of Boc Group from Sidechain or α-Amines of Amino Acids

The Boc-protected amino acid (1 eq) was dissolved in an appropriate volume of DCM to achieve a concentration of 0.1 M. TFA was then added to reach 20% acid by volume. The resulting mixture was stirred at room temperature until the Boc deprotection reaction was deemed complete by TLC (2 - 2.5 h). DCM was removed by rotary evaporation, and excess TFA was removed through 3 successive co-evaporation with more DCM. The resulting yellow-orange oils or beige solids

containing the TFA salts of the deprotected amino acids were carried forward without further characterization or purification.

General Procedure C: Fmoc-Protection of α-Amines of Amino Acids
This protocol was adapted from that reported by Narita *et al*. in 2016 [71]. The desired amino acid (1 eq), with a free α-amine to be Fmoc-protected, was dissolved in distilled water to produce a concentration of 0.25 M. For cases in which the starting material was carried forward directly from a Boc deprotection in *General Procedure B*, the excess TFA in the acidic aqueous solutions was first neutralized to pH 7 through dropwise addition of concentrated $NaOH_{(aq)}$ at room temperature with vigorous stirring. Next, $Na_2CO_{3(s)}$ (3.8 eq, or 10% w/v) was added to adjust the pH of the solution to roughly 9. Separately, fluorenylmethyloxycarbonyl chloride (1 eq) was dissolved in dioxane (0.25 M), and this was added to the reaction mixture dropwise while stirring at room temperature. After reaction completion (2 - 2.5 h), the crude product was concentrated through rotary evaporation and diluted into distilled water. This was then washed twice with ether before acidification to pH 1-2 by adding 1 M $HCl_{(aq)}$. The acidified aqueous phase was extracted thrice into ethyl acetate, and the combined organics were then washed twice with water, twice with brine, dried over $MgSO_4$, filtered, and concentrated in vacuo to yield pure products.

General Procedure D: Loading of 2-Chlorotrityl Chloride Resin with Fmoc-Protected Amino Acid
Fresh 2-chlorotrityl chloride resin (1 eq) was placed in an oven-dried hand-shaker reaction vessel equipped with a magnetic stir bar. The system was sealed with a septum and a nitrogen atmosphere was prepared with a balloon. Dry DCM (10 mL) was then added, and the resin was stirred to swell it at room temperature for 30 min. After draining the swelling solution, the loading mixture (1.2 eq Fmoc-AA-OH, 4.8 eq DIPEA, 10 mL dry DCM) was added and the reaction was stirred at room temperature under nitrogen for 2 h. After completion, the resin was washed thrice with 5 mL of DCM. The capping solution (30 mL of 17:2:1 DCM/MeOH/DIPEA) was added to the loaded resin in 3 portions of 10 mL, and the resin was stirred at room temperature for 10 min with each portion prior to draining. After capping, the resin was washed thrice with 5 mL DCM, thrice with 5 mL DMF, and thrice more with 5 mL DCM. The resulting capped and loaded resin was air-dried. To test the loading, 1-2 mg of resin was suspended in 3.0 mL of deprotection solution (20% v/v piperidine in DMF). After 1 h of vigorous shaking, 1.0 mL of the solution was diluted to a final volume of 3.0 mL with more deprotection solution and placed in a 1-cm quartz cuvette. Absorbance at 301 nm for this 1/3 dilution was then measured and corrected with a blank. The molar extinction coefficient of 8021 M^{-1} cm^{-1} for the Fmoc-piperidine adduct was used to estimate resin loading [72], while overall loading yields were determined based on the final mass of loaded resin recovered.

General Procedure E: Solid-Phase Peptide Synthesis of Acrylamide-Bearing Inhibitors
Manually-loaded Fmoc-Pro-CTC resin **10** (1 eq) was placed into the automated peptide synthesizer and swelled in 10 mL DMF for 5 min. Deprotections were performed using 3 mL of 20% v/v piperidine in DMF at 90 °C for 1 min. Couplings were done at 50 °C over 10 min with 5 eq of the appropriate Fmoc-AA-OH, 5 eq of HATU or HBTU, and 10 eq of DIPEA in 4.0 mL of a 7:1 DMF/NMP solution. The acrylamide warhead-bearing residues were introduced as the corresponding Fmoc-AA(acrylamide)-OH compounds. After linear chain completion, the resin was removed from the synthesizer and the peptides were manually acetylated at the N-terminus using 5 eq Ac_2O and 10 eq DIPEA in 5 mL DCM for 40 min at room temperature. The resin was

washed 3-9 times with DMF and/or DCM between each step. Cleavage was done with 5 mL of the standard 95:2.5:2.5 TFA/H_2O/TIPS cocktail while stirring at room temperature for 3 h. Elution with DCM, removal of excess liquids through rotary evaporation, precipitation in cold ether, centrifugation, and discarding of the supernatant produced the crude peptides. Peptides were purified through semi-preparative HPLC and were obtained as solids after lyophilization. Purity was assessed through analytical HPLC, while identity was confirmed by HRMS.

General Procedure F: Alloc-Protection of Sidechain Amines of Amino Acids
The appropriate amino acid (1 eq) bearing a free sidechain amine was first dissolved in distilled water to produce a concentration of 0.4 M, then 2.5 eq of $K_2CO_{3(s)}$ was added. For cases in which the starting material was carried forward directly from a Boc deprotection in *General Procedure B*, the excess TFA in the acidic aqueous solutions was neutralized to pH 7 prior to the addition of the carbonate base through dropwise addition of concentrated $NaOH_{(aq)}$ at room temperature with vigorous stirring. Allyl chloroformate (1.2 eq), dissolved in a volume of dioxane equivalent to that of the distilled water, was then added to the reaction dropwise. The reaction was stirred at room temperature overnight. Upon completion, the crude mixture was concentrated by rotary evaporation, then diluted into an appropriate amount of distilled water. This solution was first washed twice with ether, then acidified to pH 1-2 through the addition of 1 M $HCl_{(aq)}$. The acidified aqueous phase was subsequently extracted thrice into ethyl acetate. The combined organics were washed twice with brine, dried over $MgSO_4$, filtered, and concentrated *in vacuo* to produce pure products.

General Procedure G: Solid-Phase Peptide Synthesis of α-Chloroacetamide-Bearing Inhibitors
These syntheses were commenced in a manner identical to that described in *General Procedure E* up until the completion of acetylation. The warhead-bearing amino acid was introduced as Fmoc-AA(Alloc)-OH. For Alloc deprotection, the resin was first suspended in 5 mL DCM and 5 mL DMF, then 15 eq of $PhSiH_3$ was added. The resulting solution was sparged with a nitrogen balloon and septum to prepare an inert atmosphere before the addition of 0.3 eq of $Pd(PPh_3)_4$. The reaction was stirred under nitrogen at room temperature for 40 min. After completion, the resin was washed thrice with 10 mL of 0.02 M sodium diethyldithiocarbamate in DMF while stirring for 5-10 min during each wash. To add on the warhead, 5 mL DCM, 10 eq DIPEA, and 5 eq chloroacetic anhydride were added and the reaction was stirred at room temperature for 40 min. Washes with DCM and DMF were performed between each step. Peptides were then cleaved, precipitated, purified, and characterized as in reported in *General Procedure E*.

General Procedure H: Di-Boc Protection of α-Amines of Amino Acids
Di-Boc protections were performed using a protocol adapted from that reported by Buchold *et al*. in 2016 [53]. The amino acid bearing a singular Boc group on its α-amine (1 eq) was first dissolved in ACN (1.3 M). DMAP (0.2 eq) was added in one portion, and an inert nitrogen atmosphere was prepared using a balloon and septum. Separately, $(Boc)_2O$ (2 eq) was dissolved in ACN (0.8 M), and this solution was then added to the reaction mixture over 5 mins. After allowing the reaction to stir overnight at room temperature under nitrogen, the crude was concentrated through rotary evaporation and purified by flash column chromatography (10% EtOAc in Pet. Ether). Pure products were obtained after concentration in vacuo.

General Procedure I: Reduction of Sidechain Esters to Aldehydes

This protocol was adapted from that reported by Buchold *et al*. in 2016 [53]. The appropriate amino acid bearing a sidechain ester (1 eq) was transferred to a flame-dried or oven-dried round-bottom flask under inert nitrogen atmosphere, and was subsequently dissolved in dry Et_2O (0.16 M). The resulting solution was then cooled to -78 °C in an acetone-dry ice bath, and stirring was commenced. A 1 M solution of DIBAL in hexane (1.1 eq) was then added dropwise (0.1 to 0.2 mL/min) using a syringe pump while continuing to stir and cool at -78 °C under inert atmosphere. After the addition was complete, the reaction was stirred under N_2 for another 1 h, at which point the process was quenched through the dropwise addition of 0.5 to 3.0 mL of MeOH followed by 10 to 50 mL of a saturated aqueous solution of sodium potassium tartrate tetrahydrate. The mixture was allowed to gradually warm to room temperature while stirring under inert atmosphere for 1 to 2 h. The organic phase was then separated, washed 1-3 times with 15 mL of saturated aqueous sodium potassium tartrate tetrahydrate, dried over $MgSO_4$, filtered, and concentrated in-vacuo. Pure aldehydes were obtained at this point in the case of methyl ester reduction, so these products were carried forward without further purification. For the reduction of benzyl esters, the crude aldehydes were purified by flash column chromatography (12% EtOAc in Pet. Ether) to obtain pure products.

General Procedure J: Installation of α,β-Unsaturated Methyl Ester on Sidechain Aldehydes

This protocol was adapted from that reported by Buchold *et al*. in 2016 [53].The corresponding aldehyde (1 eq) was dissolved in benzene (1.6 M) under a nitrogen balloon atmosphere, and the solution was stirred at room temperature. Next, methyl (triphenylphosphoranylidene)acetate (1 eq) was added in 1 portion, along with the minimal amount of benzene needed to dissolve the reagent. The reaction was stirred overnight at room temperature under N_2. Upon completion, excess solvent was removed through rotary evaporation, and the crude was purified by flash column chromatography (10% EtOAc in Pet. Ether) before concentration *in vacuo* to produce pure product.

General Procedure K: Di-Boc and Tert-Butyl Ester Deprotection Followed by Mono-Boc Reprotection

This protocol was adapted from that reported by Buchold *et al*. in 2016 [53]. The appropriate amino acid bearing a di-Boc-protected amine and *tert*-butyl ester (1 eq) was dissolved in a 1:1.75 DCM/TFA mixture (0.14 M) and stirred at room temperature for 4 h. Upon completion, the resulting mixture was concentrated through rotary evaporation. Excess TFA was removed through 3 successive co-evaporations with more DCM. The deprotected intermediate was used in the subsequent reaction without further purification or characterization. An excess of DIPEA, around 6 mL, was then added to the crude intermediate to neutralize excess TFA and produce a pH 9 environment. The resulting oil was dissolved in DMF (0.32 M) and another 2 eq of DIPEA was added. Next, 1.2 eq of $(Boc)_2O$ was added in one portion, and the reaction was stirred at room temperature overnight. Upon completion, solvents were removed through rotary evaporation. The crude residue was dissolved in a 5% aqueous $KHSO_4$ solution, and this was extracted thrice into equal volumes of EtOAc. The combined organics were washed twice with equal volumes of brine before drying over $MgSO_4$, filtration, and concentration in-vacuo. The crude product was then purified by flash column chromatography (65:35 toluene/EtOAc + 0.5% AcOH), producing the desired compounds in high purity.

General Procedure L: Solid-Phase Peptide Synthesis of α,β-Unsaturated Ester-Bearing Inhibitors
Manually-loaded Fmoc-Pro CTC resin **10** (1 eq) was placed into the automated peptide synthesizer and swelled in 20 mL DMF for 5 min. Deprotections were performed using 10 mL of 20% v/v piperidine in DMF at 90 °C for 1 min. Couplings were done at 50 °C over 10 min with 4 eq of the appropriate Fmoc-AA-OH, 4 eq of HBTU, and 8 eq of DIPEA in 16 mL of a 7:1 DMF/NMP solution. The warhead-bearing amino acid was introduced as Boc-AA(MA)-OH (2 eq). The resin was washed 3 times with 7-10 mL of DMF between each step. After linear chain completion, the resin was removed from the synthesizer and the peptide was manually cleaved by stirring for 3 h at room temperature in a 95:2.5:2.5 TFA/TIPS/H_2O solution. Elution with DCM, removal of excess liquids through rotary evaporation, precipitation in cold ether, centrifugation, and discarding of the supernatant produced the crude peptide. This was subsequently dissolved in DMF and concentrated further through rotary evaporation. The TFA salts of the desired peptides were obtained after extensive drying in vacuo, and their identities were confirmed through LC-MS analysis. Crude peptides were carried forward without further purification.

General Procedure M: In-Solution Coupling of N-Terminal Residue of α,β-Unsaturated Ester-Bearing Inhibitors
The TFA salts of the peptide fragments, assembled in *General Procedure L*, leading to the desired ester-bearing inhibitors, were coupled in-solution to the final N-terminal residue through a reported protocol [51]. The peptide of the form TFA.H-AA(MA)-Nle-Nle-Leu-Pro-Trp-Pro-OH (1 eq) was dissolved in DMF (0.12 M) and stirred at room temperature. Separately, the appropriate N-terminal fragment bearing a free acid, namely either Ac-D-Asp(OtBu)-OH **38** or RhB-Pro-6AH-D-Asp(OtBu)-OH **91** (1 eq), was dissolved in DMF (0.12 M) and stirred in an ice-water bath. HATU (1.0 eq) and DIPEA (3.0 eq) were then added to the cooled amino acid solution, and this was stirred at 0 °C for 30 min. Next, the activated amino acid solution was added dropwise to the peptide solution, and the resulting mixture was set to pH 8 through the addition of more DIPEA. The reaction was left to stir overnight, and solvents were removed through rotary evaporation upon completion. Approximately one-third of the crude residue was then purified by semi-preparative HPLC, while the remaining two-thirds were stored for later use. After concentration in vacuo, peptide identity was confirmed through LC-MS, and products were carried forward without further characterization.

General Procedure N: In-Solution Final Deprotection of t-Bu Ester Group of α,β-Unsaturated Ester-Bearing Inhibitors
The final ester-bearing peptidic inhibitors were obtained following *t*-Bu ester deprotection of peptides produced in *General Procedure M* through a reported method [51]. Peptides of the form Ac-D-Asp(OtBu)-AA(MA)-Nle-Nle-Leu-Pro-Trp-Pro-OH or RhB-Pro-6AH-D-Asp(OtBu)-Glu(MA)-Nle-Nle-Leu-Pro-Trp-Pro-OH (1 eq) were dissolved in DCM (0.0064 M), then TIPS was added (7.3 eq). Next, TFA (volume half of DCM) was added, and the reaction was stirred at room temperature for 3 h. Upon completion, excess liquids were removed through rotary evaporation, and the product was purified by semi-preparative HPLC. Solid products were obtained after lyophilization. Purity was assessed through analytical HPLC, while identity was confirmed by HRMS.

General Procedure O: Acylation or Sulfonylation of N-Boc-Piperazine

To a round bottom flask was added N-Boc-Piperazine (1.1 eq) solubilized in a mixture of dichloromethane and triethylamine (1.1 eq). The corresponding acid chloride or sulfonyl chloride **48-50** (1 eq) was subsequently added portion wise, and the solution was stirred at room temperature overnight. Upon completion, the solution was washed three times with 1 M HCl, water, a saturated solution of sodium bicarbonate, and brine. In the synthesis of compound **1** the acid and water washes were excluded. The organic phase was dried with $MgSO_4$, filtered, and concentrated under reduced pressure. The crude oil was purified via flash chromatography to yield the product.

General Procedure P: Boc Deprotection of Small Molecule Intermediates

To a round bottom flask, the Boc-protected amine (1 eq) was dissolved in dichloromethane. Trifluoroacetic acid (10 % v/v) was added to the solution. The reaction mixture was stirred at room temperature overnight. Upon completion, a saturated solution of $NaHCO_3$ was added to the reaction flask and allowed to stir for half an hour. The solution was then washed with $NaHCO_3$ five more times and extracted with dichloromethane. The organic phase was dried with $MgSO_4$, filtered, and concentrated under reduced pressure. The resulting amine was carried forward without further purification.

General Procedure Q: Amide Coupling

To a round bottom flask was added the acid (1 eq), EDC HCl (1.2 eq), HOBt (1.2 eq), and DIPEA (3.0 eq) solubilized in dichloromethane and stirred for half an hour. The amine (1.2 eq) was then added, and the solution was stirred at room temperature overnight. Upon completion, the reaction mixture was washed three times with 1 M HCl, water, a saturated solution of $NaHCO_3$, and brine. In the synthesis of compound **57** the acid and water washes were excluded. The organic phase was dried with $MgSO_4$, filtered, and concentrated under reduced pressure. The crude oil was purified via flash chromatography to yield the product.

General Procedure R: Hydrogenolysis of Small Molecule Intermediates

The Cbz-protected starting material (1 eq) was dissolved in dry methanol under nitrogen gas in a round bottom flask. Palladium on carbon (20 mol%) was added and the flask was evacuated under vacuum and backfilled three times with nitrogen gas. The flask was then evacuated under vacuum and backfilled three times with hydrogen gas and equipped with a balloon of hydrogen gas. The solution was stirred overnight at room temperature. Upon completion, the hydrogen gas was removed, the reaction mixture was filtered over celite, and concentrated under reduced pressure. The resulting product was carried forward without further purification.

General Procedure S: Acrylation of Small Molecule Intermediates

To a round bottom flask was added the free amine starting material (1 eq) solubilized in dichloromethane. To the flask was subsequently added triethylamine (2.4 eq) and acryloyl chloride (2.4 eq) dropwise. The reaction was stirred for 2 h. Upon completion the reaction mixture was washed three times with water and a saturated solution of $NaHCO_3$. The aqueous phase was re extracted with DCM and the organic layers were combined, dried with $MgSO_4$, filtered, and concentrated under reduced pressure. The resulting crude oil was purified via flash chromatography to yield the product.

General Procedure T: Methyl Ester Hydrolysis
To a round bottom flask was added the methyl ester starting material (1 eq) solubilized in a 1:1 mixture of THF:H_2O. To the flask was subsequently added LiOH H_2O (2 eq) and the reaction mixture was stirred for 4 h. Upon completion, the volatiles were removed under reduced pressure and the crude oil was purified via flash chromatography to yield the product.

General Procedure U: Anhydride Coupling
To a round bottom flask was added the starting free amine (1 eq) along with triethylamine (1 eq) solubilized in DCM. To the flask was subsequently added the corresponding anhydride (1.2 eq) and triethylamine (1.5 eq). The reaction mixture was then stirred overnight at room temperature. Upon completion, the reaction mixture was concentrated under reduced pressure and the resulting crude oil was purified via flash chromatography to yield the product.

*Boc-Dap(Acrylamide)-OH (**1**).* Compound **1** was prepared from commercially available Boc-Dap-OH (500 mg, 2.45 mmol) through *General Procedure A*, and was obtained as a clear, colourless oil (375 mg, 1.45 mmol, 59% yield). ^{1}H NMR (400 MHz, CD_3OD) δ 6.28 – 6.15 (m, 2H), 5.65 (dd, J = 7.9, 4.1 Hz, 1H), 4.32 (dd, J = 7.7, 4.6 Hz, 1H), 3.71 (dd, J = 13.8, 4.7 Hz, 1H), 3.59 (dd, J = 13.9, 7.6 Hz, 1H), 1.42 (s, 9H); ^{13}C NMR (101 MHz, CD_3OD) δ 173.66, 168.61, 157.68, 131.64, 127.16, 80.65, 54.97, 41.67, 28.65; HRMS (ESI-QTOF) *m/z* $[M + Na]^+$ calcd for $C_{11}H_{18}N_2O_5Na$ 281.1113; found 281.1102.

*Boc-Dab(Acrylamide)-OH (**2**).* Compound **2** was prepared from commercially available Boc-Dab-OH (481 mg, 2.20 mmol) through *General Procedure A*, and was obtained as a clear, colourless oil (487 mg, 1.79 mmol, 81% yield). ^{1}H NMR (400 MHz, CD_3OD) δ 6.26 – 6.17 (m, 2H), 5.65 (dd, J = 7.6, 4.4 Hz, 1H), 4.14 (dd, J = 9.4, 4.7 Hz, 1H), 3.48 – 3.35 (m, 1H), 3.33 – 3.21 (m, 1H), 2.06 (1, 1H), 1.91 – 1.79 (m, 1H), 1.49 – 1.38 (s, 9H); ^{13}C NMR (101 MHz, CD_3OD) δ 175.66, 168.25, 158.15, 131.96, 126.70, 80.60, 52.72, 41.75, 32.27, 28.70; HRMS (ESI-QTOF) *m/z* $[M + Na]^+$ calcd for $C_{12}H_{20}N_2O_5Na$ 295.1270; found 295.1275.

*Boc-Orn(Acrylamide)-OH (**3**).* Compound **3** was prepared from commercially available Boc-Orn-OH (500 mg, 2.15 mmol) through *General Procedure A*, and was obtained as a white solid (447 mg, 1.56 mmol, 72% yield). ^{1}H NMR (400 MHz, CD_3OD) δ 6.23 – 6.13 (m, 2H), 5.61 (dd, J = 8.4, 3.6 Hz, 1H), 4.13 – 4.02 (m, 1H), 3.25 (t, J = 6.5 Hz, 2H), 1.89 – 1.75 (m, 1H), 1.72 – 1.52 (m, 3H), 1.40 (s, 9H); ^{13}C NMR (101 MHz, CD_3OD) δ 175.79, 167.98, 157.86, 131.92, 126.63, 80.38, 54.51, 39.88, 30.16, 28.72, 26.76; HRMS (ESI-QTOF) *m/z* $[M + Na]^+$ calcd for $C_{13}H_{22}N_2O_5Na$ 309.1426; found 309.1411.

*Boc-Lys(Acrylamide)-OH (**4**).* Compound **4** was prepared from commercially available Boc-Lys-OH (500 mg, 2.03 mmol) through *General Procedure A*, and was obtained as a clear, colourless oil (516 mg, 1.72 mmol, 85% yield). ^{1}H NMR (400 MHz, CD_3OD) δ 6.25 – 6.19 (m, 2H), 5.63 (dd, J = 7.9, 4.2 Hz, 1H), 4.14 – 4.04 (m, 1H), 3.26 (t, J = 6.9 Hz, 2H), 1.89 – 1.76 (m, 1H), 1.73 – 1.61 (m, 1H), 1.61 – 1.51 (m, 2H), 1.48 – 1.41 (m, 11H); ^{13}C NMR (101 MHz, CD_3OD) δ 176.17, 168.08, 158.10, 132.05, 126.51, 80.43, 54.76, 40.09, 32.41, 29.88, 28.72, 24.27; HRMS (ESI-QTOF) *m/z* $[M + Na]^+$ calcd for $C_{14}H_{24}N_2O_5Na$ 323.1583; found 323.1589.

*Fmoc-Dap(Acrylamide)-OH (**5**).* Compound **5** was prepared from Boc-Dap(Acrylamide)-OH **1** (375 mg, 1.45 mmol) through *General Procedure B* followed by *General Procedure C,* and was obtained as a white solid (342 mg, 0.90 mmol, 62% yield). ^{1}H NMR (400 MHz, CD_3OD) δ 7.68 (d, J = 7.5 Hz, 2H), 7.55 (dd, J = 7.1, 4.6 Hz, 2H), 7.30 (t, J = 7.5 Hz, 2H), 7.23 (td, J = 7.5, 1.2 Hz, 2H), 6.26 – 6.20 (m, 2H), 5.66 – 5.59 (m, 1H), 4.43 (dd, J = 7.8, 4.7 Hz, 1H), 4.32 – 4.20 (m, 2H), 4.10 (t, J = 7.0 Hz, 1H), 3.78 (dd, J = 13.9, 4.7 Hz, 1H), 3.64 (dd, J = 13.9, 7.8 Hz, 1H); ^{13}C NMR (101 MHz, CD_3OD) δ 173.41, 168.68, 158.34, 145.03, 144.97, 142.34, 131.55, 128.65, 128.03, 127.30, 126.11, 120.81, 68.08, 55.33, 48.10, 41.53; HRMS (ESI-QTOF) *m/z* $[M + Na]^+$ calcd for $C_{21}H_{20}N_2O_5Na$ 403.1270; found 403.1273.

*Fmoc-Dab(Acrylamide)-OH (**6**).* Compound **6** was prepared from Boc-Dab(Acrylamide)-OH **2** (487 mg, 1.79 mmol) through *General Procedure B* followed by *General Procedure C,* and was obtained as a white solid (502 mg, 1.27 mmol, 71% yield). ^{1}H NMR (400 MHz, CD_3OD) δ 7.70 (d, J = 7.6 Hz, 2H), 7.65 – 7.56 (m, 2H), 7.36 – 7.29 (m, 2H), 7.29 – 7.22 (m, 2H), 6.28 – 6.21 (m, 2H), 5.67 – 5.59 (m, 1H), 4.40 – 4.23 (m, 3H), 4.18 – 4.09 (m, 1H), 3.54 – 3.40 (m, 1H), 3.38 – 3.24 (m, 1H), 2.22 – 2.11 (m, 1H), 1.96 – 1.88 (m, 1H); ^{13}C NMR (101 MHz, CD_3OD) δ 175.35, 168.24, 158.54, 145.10, 144.95, 142.37, 142.33, 131.76, 128.65, 128.03, 126.89, 126.10, 120.82, 67.92, 53.07, 48.18, 37.19, 32.06; HRMS (ESI-QTOF) *m/z* $[M + Na]^+$ calcd for $C_{22}H_{22}N_2O_5Na$ 417.1426; found 417.1412.

*Fmoc-Orn(Acrylamide)-OH (**7**).* Compound **7** was prepared from Boc-Orn(Acrylamide)-OH **3** (447 mg, 1.56 mmol) through *General Procedure B* followed by *General Procedure C,* and was obtained as a white solid (225 mg, 0.55 mmol, 35% yield). ^{1}H NMR (400 MHz, CD_3OD) δ 7.72 (d, J = 7.4 Hz, 2H), 7.61 (t, J = 8.2 Hz, 2H), 7.33 (t, J = 7.4 Hz, 2H), 7.25 (td, J = 7.5, 0.9 Hz, 2H), 6.29 – 6.14 (m, 2H), 5.62 (dd, J = 6.8, 5.2 Hz, 1H), 4.30 (dd, J = 7.0, 4.9 Hz, 2H), 4.23 – 4.11 (m, 2H), 3.27 (t, J = 6.8 Hz, 2H), 1.96 – 1.82 (m, 1H), 1.78 – 1.52 (m, 3H); ^{13}C NMR (101 MHz, CD_3OD) δ 175.64, 168.11, 158.55, 145.21, 145.01, 142.44, 131.93, 128.69, 128.08, 126.65, 126.17, 126.14, 120.85, 67.86, 55.04, 48.27, 39.90, 30.07, 26.87; HRMS (ESI-QTOF) *m/z* $[M + Na]^+$ calcd for $C_{23}H_{24}N_2O_5Na$ 431.1583; found 431.1569.

*Fmoc-Lys(Acrylamide)-OH (**8**).* Compound **8** was prepared from Boc-Lys(Acrylamide)-OH **4** (516 mg, 1.72 mmol) through *General Procedure B* followed by *General Procedure C,* and was obtained as a white solid (599 mg, 1.42 mmol, 83% yield). ^{1}H NMR (400 MHz, CD_3OD) δ 7.68 (d, J = 7.6 Hz, 2H), 7.58 (t, J = 8.4 Hz, 2H), 7.30 (t, J = 7.5 Hz, 2H), 7.23 (td, J = 7.4, 1.1 Hz, 2H), 6.27 – 6.13 (m, 2H), 5.58 (t, J = 6.0 Hz, 1H), 4.29 (dd, J = 7.1, 3.2 Hz, 1H), 4.17 (dd, J = 9.1, 4.7 Hz, 1H), 4.11 (t, J = 7.0 Hz, 2H), 3.22 (t, J = 6.9 Hz, 2H), 1.91 – 1.78 (m, 1H), 1.76 – 1.62 (m, 1H), 1.60 – 1.47 (m, 2H), 1.47 – 1.35 (m, 2H); ^{13}C NMR (101 MHz, CD_3OD) δ 175.84, 168.07, 158.50, 145.14, 144.94, 142.38, 131.86, 128.67, 128.05, 128.03, 126.68, 126.13, 126.09, 120.84, 67.83, 55.11, 48.21, 40.09, 32.21, 29.71, 24.17; HRMS (ESI-QTOF) *m/z* $[M + Na]^+$ calcd for $C_{24}H_{26}N_2O_5Na$ 445.1739; found 445.1728.

*Fmoc-*D-*Asp(OtBu)-OH (**9**).* Compound **9** was prepared from commercially-available H-D-Asp(OtBu)-OH (2.00 g, 10.6 mmol) through *General Procedure C,* and was obtained as a white solid (3.99 g, 9.70 mmol, 92% yield). ^{1}H NMR (400 MHz, CD_3OD) δ 7.79 (d, *J* = 7.5 Hz, 2H), 7.70 – 7.62 (m, 2H), 7.41 – 7.36 (m, 2H), 7.34 – 7.26 (m, 2H), 4.60 – 4.50 (m, 1H), 4.34 (d, *J* = 7.5 Hz, 2H), 4.23 (t, *J* = 7.1 Hz, 1H), 2.81 (dd, *J* = 16.0, 5.3 Hz, 1H), 2.68 (dd, *J* = 16.1, 7.9 Hz,

1H), 1.44 (s, 9H); ^{13}C NMR (101 MHz, CD_3OD) δ 174.30, 171.38, 158.35, 145.27, 145.17, 142.57, 128.78, 128.16, 126.27, 120.91, 82.44, 68.14, 52.08, 48.32, 38.74, 28.28; HRMS (ESI-QTOF) *m/z* $[M + Na]^+$ calcd for $C_{23}H_{25}NO_6Na$ 434.1580; found 434.1584.

*Fmoc-Pro-CTC (**10**)*. Compound **10** was prepared from fresh 2-chlorotrityl chloride resin (100-200 mesh, 1.00 g, 1.06 mmol, 1 eq) and commercially available Fmoc-Pro-OH (1.2 eq) through *General Procedure D*. The resin loading was estimated as 0.664 mmol/g, and 1.29 g of loaded resin was recovered (0.853 mmol, 81% yield).

*Ac-*D*-Asp-Dap(Acrylamide)-Nle-Nle-Leu-Pro-Trp-Pro-OH (**11**)*. Compound **11** was prepared through *General Procedure E* using commercially available amino acids in addition to Fmoc-Pro-CTC resin **10** (0.1 mmol, 1 eq), Fmoc-D-Asp(OtBu)-OH **9** (0.5 mmol, 5 eq), and Fmoc-Dap(Acrylamide)-OH **5** (0.5 mmol, 5 eq) prepared herein. The desired peptide was obtained as a beige-white solid (10.0 mg, 0.0097 mmol, 10% yield). HRMS (ESI-QTOF) *m/z* $[M + Na]^+$ calcd for $C_{51}H_{74}N_{10}O_{13}Na$ 1057.5335; found 1057.5308.

*Ac-*D*-Asp-Dab(Acrylamide)-Nle-Nle-Leu-Pro-Trp-Pro-OH (**12**)*. Compound **12** was prepared through *General Procedure E* using commercially available amino acids in addition to Fmoc-Pro-CTC resin **10** (0.1 mmol, 1 eq), Fmoc-D-Asp(OtBu)-OH **9** (0.5 mmol, 5 eq), and Fmoc-Dab(Acrylamide)-OH **6** (0.5 mmol, 5 eq) prepared herein. The desired peptide was obtained as a white solid (8.6 mg, 0.0082 mmol, 8% yield). HRMS (ESI-QTOF) *m/z* $[M + Na]^+$ calcd for $C_{52}H_{76}N_{10}O_{13}Na$ 1071.5491; found 1071.5516.

*Ac-*D*-Asp-Orn(Acrylamide)-Nle-Nle-Leu-Pro-Trp-Pro-OH (**13**)*. Compound **13** was prepared through *General Procedure E* using commercially available amino acids in addition to Fmoc-Pro-CTC resin **10** (0.1 mmol, 1 eq), Fmoc-D-Asp(OtBu)-OH **9** (0.5 mmol, 5 eq), and Fmoc-Orn(Acrylamide)-OH **7** (0.5 mmol, 5 eq) prepared herein. The desired peptide was obtained as a white solid (5.0 mg, 0.0047 mmol, 5% yield). HRMS (ESI-QTOF) *m/z* $[M + Na]^+$ calcd for $C_{53}H_{78}N_{10}O_{13}Na$ 1085.5648; found 1085.5608.

*Ac-*D*-Asp-Lys(Acrylamide)-Nle-Nle-Leu-Pro-Trp-Pro-OH (**14**)*. Compound **14** was prepared through *General Procedure E* using commercially available amino acids in addition to Fmoc-Pro-CTC resin **10** (0.1 mmol, 1 eq), Fmoc-D-Asp(OtBu)-OH **9** (0.5 mmol, 5 eq), and Fmoc-Lys(Acrylamide)-OH **8** (0.5 mmol, 5 eq) prepared herein. The desired peptide was obtained as a white solid (6.7 mg, 0.0062 mmol, 6% yield). HRMS (ESI-QTOF) *m/z* $[M + Na]^+$ calcd for $C_{54}H_{80}N_{10}O_{13}Na$ 1099.5804; found 1099.5751.

*Boc-Dap(Alloc)-OH (**15**)*. Compound **15** was prepared from commercially available Boc-Dap-OH (500 mg, 2.45 mmol) through *General Procedure F*, and was obtained as a white solid (643 mg, 2.23 mmol, 91% yield). ^{1}H NMR (400 MHz, CD_3OD) δ 5.91 (ddt, J = 17.3, 10.6, 5.4 Hz, 1H), 5.28 (dd, J = 17.3, 1.7 Hz, 1H), 5.16 (dd, J = 10.5, 1.6 Hz, 1H), 4.52 (d, J = 5.4 Hz, 2H), 4.28 (dd, J = 7.4, 4.6 Hz, 1H), 3.62 – 3.51 (m, 1H), 3.49 – 3.38 (m, 1H), 1.43 (s, 9H); ^{13}C NMR (101 MHz, CD_3OD) δ 173.73, 158.66, 157.56, 134.10, 117.54, 80.56, 66.41, 55.08, 43.00, 28.67; HRMS (ESI-QTOF) *m/z* $[M + Na]^+$ calcd for $C_{12}H_{20}N_2O_6Na$ 311.1219; found 311.1214.

Boc-Orn(Alloc)-OH (16). Compound **16** was prepared from commercially available Boc-Orn-OH (500 mg, 2.15 mmol) through *General Procedure F,* and was obtained as a clear, colourless oil (642 mg, 2.03 mmol, 94% yield). ^{1}H NMR (300 MHz, CD_3OD) δ 5.92 (ddt, *J* = 17.2, 10.6, 5.4 Hz, 1H), 5.28 (dd, *J* = 17.4, 1.7 Hz, 1H), 5.16 (dd, *J* = 10.5, 1.5 Hz, 1H), 4.51 (dt, *J* = 5.4, 1.6 Hz, 2H), 4.16 – 4.03 (m, 1H), 3.13 (t, *J* = 6.5 Hz, 2H), 1.94 – 1.74 (m, 1H), 1.74 – 1.51 (m, 3H), 1.44 (s, 9H); ^{13}C NMR (75 MHz, CD_3OD) δ 175.88, 158.58, 157.87, 134.39, 117.41, 80.37, 66.17, 54.51, 41.20, 30.01, 28.72, 27.27; HRMS (ESI-QTOF) *m/z* $[M + Na]^+$ calcd for $C_{14}H_{24}N_2O_6Na$ 339.1532; found 339.1534.

Fmoc-Dap(Alloc)-OH (17). Compound **17** was prepared from Boc-Dap(Alloc)-OH **15** (643 mg, 2.23 mmol), through *General Procedure B* followed by *General Procedure C,* and was obtained as a white solid (756 mg, 1.84 mmol, 83% yield). ^{1}H NMR (400 MHz, CD_3OD) δ 7.83 – 7.73 (m, 2H), 7.71 – 7.61 (m, 2H), 7.43 – 7.35 (m, 2H), 7.34 – 7.27 (m, 2H), 5.91 (ddt, *J* = 17.2, 10.7, 5.4 Hz, 1H), 5.28 (dd, *J* = 17.1, 1.7 Hz, 1H), 5.15 (dd, *J* = 10.6, 1.7 Hz, 1H), 4.59 – 4.49 (m, 2H), 4.38 – 4.30 (m, 3H), 4.23 (t, *J* = 7.0 Hz, 1H), 3.61 (dd, *J* = 14.1, 4.7 Hz, 1H), 3.45 (dd, *J* = 14.0, 7.7 Hz, 1H); ^{13}C NMR (101 MHz, CD_3OD) δ 173.60, 158.97, 158.54, 145.26, 145.24, 142.56, 134.32, 128.78, 128.17, 126.30, 120.91, 117.51, 68.19, 66.56, 55.70, 48.33, 42.98; HRMS (ESI-QTOF) *m/z* $[M + Na]^+$ calcd for $C_{22}H_{22}N_2O_6Na$ 433.1376; found 433.1378.

Fmoc-Dab(Alloc)-OH (18). Compound **18** was prepared from commercially-available Fmoc-Dab(Boc)-OH (500 mg, 1.14 mmol) through *General Procedure B* followed by *General Procedure F*, and was obtained as a white solid (477 mg, 1.12 mmol, 99% yield). ^{1}H NMR (400 MHz, CD_3OD) δ 7.70 (d, *J* = 7.5 Hz, 2H), 7.62 (t, *J* = 7.4 Hz, 2H), 7.33 (t, *J* = 7.5 Hz, 2H), 7.26 (tt, *J* = 7.5, 1.5 Hz, 2H), 5.89 (ddt, *J* = 17.2, 10.6, 5.4 Hz, 1H), 5.27 (dq, *J* = 17.2, 1.7 Hz, 1H), 5.15 (d, *J* = 10.0 Hz, 1H), 4.58 – 4.44 (m, 2H), 4.38 – 4.25 (m, 3H), 4.14 (t, *J* = 7.0 Hz, 1H), 3.37 – 3.26 (m, 1H), 3.26 – 3.13 (m, 1H), 2.20 – 2.08 (m, 1H), 1.95 – 1.79 (m, 1H); ^{13}C NMR (101 MHz, CD_3OD) δ 175.47, 158.48, 145.07, 144.93, 142.32, 134.19, 128.62, 128.01, 126.11, 120.80, 117.54, 67.88, 66.33, 52.84, 48.13, 38.40, 32.62; HRMS (ESI-QTOF) *m/z* $[M + Na]^+$ calcd for $C_{23}H_{24}N_2O_6Na$ 447.1532; found 447.1545.

Fmoc-Orn(Alloc)-OH (19). Compound **19** was prepared from Boc-Orn(Alloc)-OH **16** (642 mg, 2.03 mmol) through *General Procedure B* followed by *General Procedure C,* and was obtained as a white solid (540 mg, 1.23 mmol, 61% yield). ^{1}H NMR (400 MHz, CD_3OD) δ 7.79 (d, *J* = 7.6 Hz, 2H), 7.70 – 7.63 (m, 2H), 7.38 (d, *J* = 7.6 Hz, 2H), 7.30 (td, *J* = 7.5, 1.2 Hz, 2H), 5.92 (ddt, *J* = 17.1, 10.7, 5.4 Hz, 1H), 5.29 (dq, *J* = 17.2, 1.7 Hz, 1H), 5.17 (dq, *J* = 10.5, 1.5 Hz, 1H), 4.57 – 4.46 (m, 2H), 4.43 – 4.29 (m, 2H), 4.22 (t, *J* = 7.0 Hz, 1H), 4.15 (dd, *J* = 9.0, 4.7 Hz, 1H), 3.13 (t, *J* = 6.8 Hz, 2H), 1.95 – 1.82 (m, 1H), 1.76 – 1.50 (m, 3H); ^{13}C NMR (101 MHz, CD_3OD) δ 175.77, 158.82, 158.69, 145.35, 145.17, 142.58, 134.54, 128.77, 128.17, 128.15, 126.29, 126.27, 120.90, 117.38, 67.94, 66.28, 55.12, 48.41, 41.25, 29.93, 27.49; HRMS (ESI-QTOF) *m/z* $[M + Na]^+$ calcd for $C_{24}H_{26}N_2O_6Na$ 461.1689; found 461.1671.

*Ac-*D*-Asp-Dap(α-Chloroacetamide)-Nle-Nle-Leu-Pro-Trp-Pro-OH (20).* Compound **20** was prepared through *General Procedure G* using commercially available amino acids in addition to Fmoc-Pro-CTC resin **10** (0.1 mmol, 1 eq), Fmoc-D-Asp(OtBu)-OH **9** (0.5 mmol, 5 eq), and Fmoc-Dap(Alloc)-OH **17** (0.5 mmol, 5 eq) prepared herein. The desired peptide was obtained as a white

solid (9.6 mg, 0.0091 mmol, 9% yield). HRMS (ESI-QTOF) *m/z* $[M + Na]^+$ calcd for $C_{50}H_{73}ClN_{10}O_{13}Na$ 1079.4945; found 1079.4952.

*Ac-*D*-Asp-Dab(α-Chloroacetamide)-Nle-Nle-Leu-Pro-Trp-Pro-OH (21)*. Compound **21** was prepared through *General Procedure G* using commercially available amino acids in addition to Fmoc-Pro-CTC resin **10** (0.1 mmol, 1 eq), Fmoc-D-Asp(OtBu)-OH **9** (0.5 mmol, 5 eq), and Fmoc-Dab(Alloc)-OH **18** (0.5 mmol, 5 eq) prepared herein. The desired peptide was obtained as a white solid (11.4 mg, 0.0106 mmol, 11% yield). HRMS (ESI-QTOF) *m/z* $[M + Na]^+$ calcd for $C_{51}H_{75}ClN_{10}O_{13}Na$ 1093.5101; found 1093.5128.

*Ac-*D*-Asp-Orn(α-Chloroacetamide)-Nle-Nle-Leu-Pro-Trp-Pro-OH (22)*. Compound **22** was prepared through *General Procedure G* using commercially available amino acids in addition to Fmoc-Pro-CTC resin **10** (0.1 mmol, 1 eq), Fmoc-D-Asp(OtBu)-OH **9** (0.5 mmol, 5 eq), and Fmoc-Orn(Alloc)-OH **19** (0.5 mmol, 5 eq) prepared herein. The desired peptide was obtained as a white solid (11.6 mg, 0.0107 mmol, 11% yield). HRMS (ESI-QTOF) *m/z* $[M + Na]^+$ calcd for $C_{52}H_{77}ClN_{10}O_{13}Na$ 1107.5258; found 1107.5211.

*Ac-*D*-Asp-Lys(α-Chloroacetamide)-Nle-Nle-Leu-Pro-Trp-Pro-OH (23)*. Compound **23** was prepared through *General Procedure G* using commercially available amino acids, including Fmoc-Lys(Alloc)-OH, in addition to Fmoc-Pro-CTC resin **10** (0.1 mmol, 1 eq) and Fmoc-D-Asp(OtBu)-OH **9** (0.5 mmol, 5 eq) prepared herein. The desired peptide was obtained as a beige solid (15.5 mg, 0.014 mmol, 14% yield). HRMS (ESI-QTOF) *m/z* $[M + Na]^+$ calcd for $C_{53}H_{79}ClN_{10}O_{13}Na$ 1121.5414; found 1121.5399.

Boc-Glu(OMe)-OtBu (24). Compound **24** was prepared from commercially available Boc-Glu-OtBu as previously reported by Buchold *et al*. in 2016. Boc-Glu-OtBu (4.55 g, 15.0 mmol, 1 eq) was dissolved in 75 mL of DMF to produce a concentration of 0.2 M. An inert nitrogen atmosphere was prepared by flushing the reaction flask with a balloon. Cs_2CO_3 (0.55 eq) was then added in 1 portion, and the mixture was stirred under inert atmosphere at room temperature for 1 h. Next, MeI (1 eq) was added dropwise over 5 min, and the reaction was stirred under inert atmosphere at room temperature overnight. Upon reaction completion, the crude mixture was concentrated by rotary evaporation to remove DMF. The resulting crude residue was dissolved in 150 mL of EtOAc, and excess undissolved Cs_2CO_3 was removed by filtration. The filtrate was washed thrice with 30 mL of 10% citric acid, thrice with 30 mL of 10% $NaHCO_3$, and thrice with 30 mL of brine. The resulting organic phase was dried over $MgSO_4$, filtered, and concentrated in vacuo. Pure product was obtained as 4.33 g (13.6 mmol, 91% yield) of yellow-white solid. ^1H NMR (400 MHz, $CDCl_3$) δ 5.14 – 4.99 (m, 1H), 4.27 – 4.14 (m, 1H), 3.67 (s, 3H), 2.49 – 2.27 (m, 2H), 2.22 – 2.06 (m, 1H), 1.99 – 1.82 (m, 1H), 1.45 (s, 9H), 1.43 (s, 9H). Characterization matches previous reports [53].

Boc_2-Glu(OMe)-OtBu (25). Compound **25** was prepared from Boc-Glu(OMe)-OtBu **24** (4.33 g, 13.6 mmol, 1 eq) through *General Procedure H*. The title compound was obtained as a colourless oil (2.79 g, 6.68 mmol, 49% yield). ^1H NMR (400 MHz, $CDCl_3$) δ 4.82 – 4.72 (m, 1H), 3.65 (s, 3H), 2.48 – 2.31 (m, 3H), 2.20 – 2.09 (m, 1H), 1.48 (s, 18H), 1.42 (s, 9H). Characterization matches previous reports [53].

*Boc$_2$-Glu(H)-OtBu (**26**)*. Compound **26** was prepared from Boc$_2$-Glu(OMe)-OtBu **25** (2.79 g, 6.68 mmol, 1 eq) through *General Procedure I*, furnishing pure product as a colourless oil (2.33 g, 6.01 mmol, 90% yield). ^{1}H NMR (400 MHz, $CDCl_3$) δ 9.74 (t, J = 1.2 Hz, 1H), 4.71 (dd, J = 9.4, 5.3 Hz, 1H), 2.63 – 2.31 (m, 3H), 2.20 – 2.03 (m, 1H), 1.48 (s, 18H), 1.42 (s, 9H). Characterization matches previous reports [53].

*Boc$_2$-Glu(MA)-OtBu (**27**)*. Compound **27** was prepared from Boc$_2$-Glu(H)-OtBu **26** (2.33 g, 6.01 mmol, 1 eq) through *General Procedure J*. The title compound was obtained as a colourless oil (2.18 g, 4.92 mmol, 82% yield). ^{1}H NMR (400 MHz, $CDCl_3$) δ 6.93 (dt, J = 15.7, 6.7 Hz, 1H), 5.83 (dt, J = 15.6, 1.4 Hz, 1H), 4.69 (dd, J = 9.6, 4.7 Hz, 1H), 3.70 (s, 3H), 2.32 – 2.13 (m, 3H), 2.07 – 1.95 (m, 1H), 1.48 (s, 18H), 1.42 (s, 9H). Characterization matches previous reports [53].

*Boc-Glu(MA)-OH (**28**)*. Compound **28** was prepared from Boc$_2$-Glu(MA)-OtBu **27** (1.90 g, 4.28 mmol, 1 eq) through *General Procedure K*. The title compound was obtained as a yellow oil (1.08 g, 3.75 mmol, 88% yield). ^{1}H NMR (400 MHz, CD_3OD) δ 6.97 (dt, J = 15.6, 7.0 Hz, 1H), 5.88 (dt, J = 15.6, 1.5 Hz, 1H), 4.15 – 4.05 (m, 1H), 3.71 (s, 3H), 2.39 – 2.25 (m, 2H), 2.06 – 1.90 (m, 1H), 1.86 – 1.72 (m, 1H), 1.44 (s, 9H). Characterization matches previous reports [53].

*Boc-Asp(OBzl)-OtBu (**29**)*. Compound **29** was prepared through a Steglich esterification reported previously [73,74]. Around 4.85 g of commercially available Boc-Asp(OBzl)-OH (15 mmol, 1 eq) was added to an oven-dried round bottom flask equipped with a stir bar. This was then dissolved in 9.7 mL of dry DCM (1.6 M) under inert atmosphere (N_2 balloon). Stirring was then commenced at room temperature. Next, DMAP was added in 1 portion (0.1 eq), followed by *t*-BuOH (1.2 eq), and DCC (1.2 eq). The reaction was then stirred for 3 h at room temperature under inert atmosphere. Upon completion, the crude mixture was filtered to remove the precipitated urea of DCC, and the filtrate was concentrated through rotary evaporation. The crude residue was subsequently purified by column chromatography (10% EtOAc in hexanes), yielding 3.96 g of pure product as a colourless oil (10.4 mmol, 70% yield). ^{1}H NMR (400 MHz, $CDCl_3$) δ 7.41 – 7.27 (m, 5H), 5.49 – 5.41 (m, 1H), 5.15 (d, J = 12.3 Hz, 1H), 5.10 (d, J = 12.3 Hz, 1H), 4.50 – 4.41 (m, 1H), 2.99 (dd, J = 16.8, 4.6 Hz, 1H), 2.82 (dd, J = 16.8, 4.8 Hz, 1H), 1.44 (s, 9H), 1.41 (s, 9H); ^{13}C NMR (101 MHz, $CDCl_3$) δ 171.01, 170.07, 155.56, 135.68, 128.72, 128.49, 128.44, 82.47, 79.99, 66.78, 50.67, 37.21, 28.45, 27.96; HRMS (ESI-QTOF) *m/z* $[M + Na]^+$ calcd for $C_{20}H_{29}NO_6Na$ 402.1893; found 402.1902.

*Boc$_2$-Asp(OBzl)-OtBu (**30**)*. Compound **30** was prepared from Boc-Asp(OBzl)-OtBu **29** (4.36 g, 11.5 mmol) through *General Procedure H*, and was obtained as a colourless oil (5.20 g, 10.8 mmol, 94% yield). ^{1}H NMR (400 MHz, $CDCl_3$) δ 7.37 – 7.29 (m, 5H), 5.36 (dd, J = 7.2, 6.5 Hz, 1H), 5.18 (d, J = 12.4 Hz, 1H), 5.09 (d, J = 12.3 Hz, 1H), 3.27 (dd, J = 16.4, 7.2 Hz, 1H), 2.73 (dd, J = 16.4, 6.5 Hz, 1H), 1.49 (s, 18H), 1.43 (s, 9H); ^{13}C NMR (101 MHz, $CDCl_3$) δ 170.89, 168.75, 152.10, 135.92, 128.63, 128.34, 128.30, 83.28, 82.05, 66.67, 55.69, 35.76, 28.13, 27.99; HRMS (ESI-QTOF) *m/z* $[M + Na]^+$ calcd for $C_{25}H_{37}NO_8Na$ 502.2417; found 502.2425.

*Boc$_2$-Asp(H)-OtBu (**31**)*. Compound **31** was prepared from Boc$_2$-Asp(OBzl)-OtBu **30** (4.23 g, 8.82 mmol) through *General Procedure I*, and was obtained as a colourless oil (2.22 g, 5.94 mmol, 67% yield). ^{1}H NMR (400 MHz, $CDCl_3$) δ 9.77 (t, J = 1.4 Hz, 1H), 5.40 (dd, J = 6.9, 6.0 Hz, 1H), 3.35 (ddd, J = 17.7, 7.0, 1.6 Hz, 1H), 2.75 (ddd, J = 17.7, 6.0, 1.3 Hz, 1H), 1.50 (s, 18H), 1.43 (s, 9H);

^{13}C NMR (101 MHz, $CDCl_3$) δ 199.01, 168.79, 152.24, 83.49, 82.32, 53.88, 44.74, 28.15, 28.00; HRMS (ESI-QTOF) *m/z* $[M + Na]^+$ calcd for $C_{18}H_{31}NO_7Na$ 396.1998; found 396.2008.

*Boc_2-Asp(MA)-OtBu (**32**)*. Compound **32** was prepared from Boc_2-Asp(H)-OtBu **31** (2.73 g, 7.32 mmol) through *General Procedure J*, and was obtained as a colourless oil (2.78 g, 6.47 mmol, 88% yield). ^1H NMR (400 MHz, $CDCl_3$) δ 6.90 (ddd, J = 15.3, 8.6, 6.4 Hz, 1H), 5.85 (dt, J = 15.6, 1.5 Hz, 1H), 4.89 (dd, J = 9.8, 5.2 Hz, 1H), 3.70 (s, 3H), 3.01 – 2.89 (m, 1H), 2.86 – 2.73 (m, 1H), 1.49 (s, 18H), 1.44 (s, 9H); ^{13}C NMR (101 MHz, $CDCl_3$) δ 168.86, 166.66, 152.31, 145.12, 123.57, 83.26, 81.98, 57.77, 51.54, 32.76, 28.12, 28.04; HRMS (ESI-QTOF) *m/z* $[M + Na]^+$ calcd for $C_{21}H_{35}NO_8Na$ 452.2260; found 452.2267.

*Boc-Asp(MA)-OH (**33**)*. Compound **33** was prepared from Boc_2-Asp(MA)-OtBu **32** (1.25 g, 2.90 mmol) through *General Procedure K*, and was obtained as a faint yellow oil (608 mg, 2.22 mmol, 77% yield). ^1H NMR (400 MHz, CD_3OD) δ 6.91 (dt, J = 15.0, 7.3 Hz, 1H), 5.94 (dt, J = 15.5, 1.4 Hz, 1H), 4.25 (dd, J = 8.9, 4.9 Hz, 1H), 3.71 (s, 3H), 2.81 – 2.69 (m, 1H), 2.63 – 2.50 (m, 1H), 1.44 (s, 9H); ^{13}C NMR (101 MHz, CD_3OD) δ 174.65, 168.16, 157.89, 145.63, 124.64, 80.65, 53.89, 52.01, 35.48, 28.66; HRMS (ESI-QTOF) *m/z* $[M + Na]^+$ calcd for $C_{12}H_{19}NO_6Na$ 296.1110; found 296.1114.

*Boc_2-Hmg(OMe)-OtBu (**34**)*. Approximately 1.21 g of Boc_2-Asp(MA)-OtBu **32** (1.21 g, 2.82 mmol, 1 eq) was dissolved in MeOH (0.1 M) under inert nitrogen balloon atmosphere. Next, 10 wt. % loaded Pd/C was added in one portion (0.1 eq), and the nitrogen atmosphere was re-prepared prior to replacement with hydrogen gas. The reaction was stirred for 4 h at room temperature under hydrogen, at which point nitrogen was pumped back in and the transformation was deemed complete by TLC analysis. The mixture was then filtered over Celite and washed through with excess methanol prior to concentration in-vacuo. Pure product was obtained as 1.19 g of colourless oil (2.76 mmol, 98% yield). ^1H NMR (400 MHz, $CDCl_3$) δ 4.71 (dd, J = 9.7, 5.1 Hz, 1H), 3.65 (s, 3H), 2.43 – 2.24 (m, 2H), 2.12 – 1.99 (m, 1H), 1.96 – 1.82 (m, 1H), 1.74 – 1.61 (m, 2H), 1.50 (s, 18H), 1.43 (s, 9H); ^{13}C NMR (101 MHz, $CDCl_3$) δ 173.84, 169.81, 152.58, 82.95, 81.38, 58.64, 51.64, 33.77, 28.78, 28.15, 28.07, 21.96; HRMS (ESI-QTOF) *m/z* $[M + Na]^+$ calcd for $C_{21}H_{37}NO_8Na$ 454.2417; found 454.2428.

*Boc_2-Hmg(H)-OtBu (**35**)*. Compound **35** was prepared from Boc_2-Hmg(OMe)-OtBu **34** (1.50 g, 3.47 mmol) through *General Procedure I*, and was obtained as a colourless oil (1.24 g, 3.09 mmol, 89% yield). ^1H NMR (400 MHz, $CDCl_3$) δ 9.75 (t, J = 1.6 Hz, 1H), 4.71 (dd, J = 9.5, 5.2 Hz, 1H), 2.57 – 2.36 (m, 2H), 2.13 – 1.99 (m, 1H), 1.97 – 1.83 (m, 1H), 1.73 – 1.61 (m, 2H), 1.50 (s, 18H), 1.43 (s, 9H); ^{13}C NMR (101 MHz, $CDCl_3$) δ 202.16, 169.74, 152.64, 83.03, 81.46, 58.62, 43.53, 28.80, 28.16, 28.06, 19.14; HRMS (ESI-QTOF) *m/z* $[M + Na]^+$ calcd for $C_{20}H_{35}NO_7Na$ 424.2311; found 424.2306.

*Boc_2-Hmg(MA)-OtBu (**36**)*. Compound **36** was prepared from Boc_2-Hmg(H)-OtBu **35** (1.24 g, 3.09 mmol) through *General Procedure J*, and was obtained as a colourless oil (1.04 g, 2.28 mmol, 74% yield). ^1H NMR (400 MHz, $CDCl_3$) δ 6.94 (dt, J = 15.6, 6.9 Hz, 1H), 5.82 (dt, J = 15.6, 1.6 Hz, 1H), 4.70 (dd, J = 9.6, 5.2 Hz, 1H), 3.71 (s, 3H), 2.34 – 2.12 (m, 2H), 2.12 – 1.99 (m, 1H), 1.94 – 1.80 (m, 1H), 1.55 – 1.46 (m, 20H), 1.44 (s, 9H); ^{13}C NMR (101 MHz, $CDCl_3$) δ 169.87,

167.16, 152.64, 149.03, 121.43, 82.97, 81.39, 58.65, 51.52, 31.88, 28.82, 28.17, 28.08, 25.04; HRMS (ESI-QTOF) *m/z* $[M + Na]^+$ calcd for $C_{23}H_{39}NO_8Na$ 480.2573; found 480.2566.

Boc-Hmg(MA)-OH (37). Compound **37** was prepared from Boc_2-Hmg(MA)-OtBu **36** (1.04 g, 2.28 mmol) through *General Procedure K*, and was obtained as a faint yellow oil (614 mg, 2.04 mmol, 89% yield). ^1H NMR (400 MHz, CD_3OD) δ 6.96 (dt, J = 15.6, 7.0 Hz, 1H), 5.87 (dt, J = 15.6, 1.6 Hz, 1H), 4.17 – 4.02 (m, 1H), 3.71 (s, 3H), 2.30 – 2.20 (m, 2H), 1.89 – 1.76 (m, 1H), 1.72 – 1.53 (m, 3H), 1.44 (s, 9H); ^{13}C NMR (101 MHz, CD_3OD) δ 176.03, 168.66, 158.15, 150.39, 122.23, 80.48, 54.53, 51.93, 32.50, 32.25, 28.71, 25.42; HRMS (ESI-QTOF) *m/z* $[M + Na]^+$ calcd for $C_{14}H_{23}NO_6Na$ 324.1423; found 324.1437.

*Ac-*D*-Asp(OtBu)-OH (38)*. Compound **38** was produced from commercially available H-D-Asp(OtBu)-OH through a previously-reported method. The amino acid starting material (1.135 g, 6.00 mmol, 1.0 eq) was dissolved in dry THF (0.5 M) under inert (N_2 balloon) atmosphere in an oven-dried round-bottom flask equipped with a stir bar. While stirring at room temperature, excess Ac_2O (3.0 eq) was then added, and the reaction was allowed to stir at room temperature overnight. The reaction was then acidified to pH 2 through the addition of a minimal amount of 1 M $HCl_{(aq)}$ (approximately 2 mL), and excess solvents were subsequently removed through rotary evaporation. The water bath temperature was kept at or below 35 °C to prevent the deprotection of the *tert*-butyl ester. Excess water was removed through azeotropic evaporation with toluene, producing a white solid. The solid obtained was then cleaned through vacuum filtration while rinsing with EtOAc, yielding the desired compound as a white solid in 39% yield (540 mg, 2.33 mmol). ^1H NMR (400 MHz, CD_3OD) δ 4.73 (dd, J = 7.4, 5.4 Hz, 1H), 2.78 (dd, J = 16.1, 5.4 Hz, 1H), 2.67 (dd, J = 16.2, 7.4 Hz, 1H), 1.98 (s, 3H), 1.45 (s, 9H). Characterization matches previous reports [68].

TFA.H-Asp(MA)-Nle-Nle-Leu-Pro-Trp-Pro-OH (39). Compound **39** was prepared through *General Procedure L* using commercially available amino acids in addition to Fmoc-Pro-CTC resin **10** (0.5 mmol, 1 eq) and Boc-Asp(MA)-OH **33** (1.0 mmol, 2 eq) prepared herein. The desired peptide was obtained as a brown oil (336 mg, 0.33 mmol, 66% yield). LC-MS *m/z* 894 for $[M + H]^+$.

TFA.H-Glu(MA)-Nle-Nle-Leu-Pro-Trp-Pro-OH (40). Compound **40** was prepared through *General Procedure L* using commercially available amino acids in addition to Fmoc-Pro-CTC resin **10** (0.5 mmol, 1 eq) and Boc-Glu(MA)-OH **28** (1.0 mmol, 2 eq) prepared herein. The desired peptide was obtained as a beige solid (326 mg, 0.320 mmol, 64% yield). LC-MS *m/z* 908 for $[M + H]^+$.

TFA.H-Hmg(MA)-Nle-Nle-Leu-Pro-Trp-Pro-OH (41). Compound **41** was prepared through *General Procedure L* using commercially available amino acids in addition to Fmoc-Pro-CTC resin **10** (0.5 mmol, 1 eq) and Boc-Hmg(MA)-OH **37** (1.0 mmol, 2 eq) prepared herein. The desired peptide was obtained as a beige solid (220 mg, 0.21 mmol, 42% yield). LC-MS *m/z* 922 for $[M + H]^+$.

*Ac-*D*-Asp(OtBu)-Asp(MA)-Nle-Nle-Leu-Pro-Trp-Pro-OH* (**42**). Compound **42** was prepared through *General Procedure M* using TFA.H-Asp(MA)-Nle-Nle-Leu-Pro-Trp-Pro-OH **39** (336 mg,

0.33 mmol, 1 eq) and Ac-D-Asp(OtBu)-OH **38** (0.33 mmol, 1 eq), both prepared herein. The desired peptide was obtained as a beige solid (57.3 mg, 0.052 mmol, 16% yield). LC-MS *m/z* 1107 for $[M + H]^+$.

Ac-D-Asp(OtBu)-Glu(MA)-Nle-Nle-Leu-Pro-Trp-Pro-OH (**43**). Compound **43** was prepared through *General Procedure M* using TFA.H-Glu(MA)-Nle-Nle-Leu-Pro-Trp-Pro-OH **40** (326 mg, 0.32 mmol, 1 eq) and Ac-D-Asp(OtBu)-OH **38** (0.32 mmol, 1 eq), both prepared herein. The desired peptide was obtained as a yellow solid (36.0 mg, 0.032 mmol, 10% yield). LC-MS *m/z* 1121 for $[M + H]^+$.

Ac-D-Asp(OtBu)-Hmg(MA)-Nle-Nle-Leu-Pro-Trp-Pro-OH (**44**). Compound **44** was prepared through *General Procedure M* using TFA.H-Hmg(MA)-Nle-Nle-Leu-Pro-Trp-Pro-OH **41** (220 mg, 0.21 mmol, 1 eq) and Ac-D-Asp(OtBu)-OH **38** (0.21 mmol, 1 eq), both prepared herein. The desired peptide was obtained as a white solid (79.8 mg, 0.070 mmol, 33% yield). LC-MS *m/z* 1135 for $[M + H]^+$.

Ac-D-Asp-Asp(MA)-Nle-Nle-Leu-Pro-Trp-Pro-OH (**45**). Compound **45** was prepared through *General Procedure N* from Ac-D-Asp(OtBu)-Asp(MA)-Nle-Nle-Leu-Pro-Trp-Pro-OH **42** (57.3 mg, 0.052 mmol, 1.0 eq). The desired peptide was obtained as a white solid (7.8 mg, 0.0074 mmol, 14% yield). HRMS (ESI-QTOF) *m/z* $[M + Na]^+$ calcd for $C_{52}H_{75}N_9O_{14}Na$ 1072.5331; found 1072.5323.

Ac-D-Asp-Glu(MA)-Nle-Nle-Leu-Pro-Trp-Pro-OH (**46,** ZED1301). Compound **46** was prepared through *General Procedure N* from Ac-D-Asp(OtBu)-Glu(MA)-Nle-Nle-Leu-Pro-Trp-Pro-OH **43** (36.0 mg, 0.032 mmol, 1.0 eq). The desired peptide was obtained as a white solid (5.7 mg, 0.0054 mmol, 17% yield). HRMS (ESI-QTOF) *m/z* $[M + Na]^+$ calcd for $C_{53}H_{77}N_9O_{14}Na$ 1086.5488; found 1086.5507.

Ac-D-Asp-Hmg(MA)-Nle-Nle-Leu-Pro-Trp-Pro-OH (**47**). Compound **47** was prepared through *General Procedure N* from Ac-D-Asp(OtBu)-Hmg(MA)-Nle-Nle-Leu-Pro-Trp-Pro-OH **44** (79.8 mg, 0.070 mmol, 1.0 eq). The desired peptide was obtained as a white solid (9.3 mg, 0.0086 mmol, 12% yield). HRMS (ESI-QTOF) *m/z* $[M + Na]^+$ calcd for $C_{54}H_{79}N_9O_{14}Na$ 1100.5644; found 1100.5662.

N-Boc-Piperazine-Dansyl (**51**). Compound **51** was synthesized using a sulfonylation reaction detailed in *General Procedure O* (yield 96%). Characterization consistent with data from literature [75].

N-Boc-Piperazine-Sulfonyl-Naphthalene (**52**). Compound **52** was synthesized via an a sulfonylation reaction detailed in *General Procedure O* (yield 96%). Characterization consistent with data from literature [76].

N-Boc-Piperazine-Naphthoyl (**53**). Compound **53** was synthesized using the acylation reaction outlined in *General Procedure O* (yield 98%). Characterization consistent with data from literature [42].

*H-Piperazine-Dansyl (***54**). Compound **54** was synthesized according to the acid catalyzed Boc deprotection detailed in *General Procedure P*. The product was carried forward without further purification or characterization.

H-Piperazine-Sulfonyl-Naphthalene (**55**). Compound **55** was synthesized according to the acid catalyzed Boc deprotection detailed in *General Procedure P*. The product was carried forward without further purification or characterization.

H-Piperazine-Naphthoyl (**56**). Compound **56** was synthesized according to the acid catalyzed Boc deprotection detailed in *General Procedure P*. The product was carried forward without further purification or characterization.

*Boc-*L*-Dap(Cbz)-Piperazine-Dansyl* (**57**). Compound **57** was synthesized using an amide bond coupling outlined in *General Procedure Q* (yield 92%). ^{1}H NMR (400 MHz, $CDCl_3$) δ 8.57 (d, *J* = 8.5 Hz, 1H), 8.34 (d, *J* = 8.7 Hz, 1H), 8.19 (dd, *J* = 7.3, 1.3 Hz, 1H), 7.54 (ddt, *J* = 9.4, 7.3, 4.2 Hz, 2H), 7.35 – 7.22 (m, 5H), 7.17 (d, *J* = 7.5 Hz, 1H), 5.50 (d, *J* = 8.3 Hz, 1H), 5.37 (s, 1H), 5.01 (s, 2H), 4.66 (s, 1H), 3.73 – 3.02 (m, 10H), 2.87 (s, 6H), 1.35 (s, 9H). ^{13}C NMR (101 MHz, $CDCl_3$) δ 168.60, 156.64, 155.41, 151.93, 136.39, 132.40, 131.12, 130.82, 130.33, 130.14, 128.54, 128.37, 128.20, 128.08, 125.10, 123.23, 120.23, 119.36, 115.44, 80.20, 66.85, 50.18, 45.62, 45.47, 45.18, 43.74, 41.78, 28.30. HRMS (ESI-QTOF) m/z [M + Na]+ calcd for $C_{32}H_{41}N_5O_7SNa$ 662.2624; found 662.2599.

*Boc-*L*-Dap(Cbz)-Piperazine-Sulfonyl-Naphthalene* (**58**). Compound **58** was synthesized using an amide bond coupling outlined in *General Procedure Q* (yield 85%). ^{1}H NMR (400 MHz, $CDCl_3$) δ 8.72 (d, *J* = 8.6 Hz, 1H), 8.21 (d, *J* = 7.4 Hz, 1H), 8.10 (d, *J* = 8.2 Hz, 1H), 7.94 (d, *J* = 8.0 Hz, 1H), 7.70 – 7.54 (m, 3H), 7.37 – 7.27 (m, 5H), 5.38 (d, *J* = 8.2 Hz, 1H), 5.20 (t, *J* = 6.2 Hz, 1H), 5.02 (s, 2H), 4.65 (s, 1H), 3.79 – 3.02 (m, 10H), 1.37 (s, 9H). ^{13}C NMR (101 MHz, $CDCl_3$) δ 168.42, 156.31, 155.35, 134.90, 134.42, 132.13, 130.79, 129.08, 128.87, 128.51, 128.40, 128.18, 128.03, 127.05, 124.88, 124.19, 80.29, 66.87, 50.15, 45.60, 45.18, 43.82, 41.72, 28.23. HRMS (ESI-QTOF) m/z [M + Na]+ calcd for $C_{30}H_{36}N_4O_7SNa$ 619.2202; found 619.2208.

*Boc-*L*-Dap(Cbz)-Piperazine-Naphthoyl* (**59**). Compound **59** was synthesized using an amide bond coupling outlined in *General Procedure Q* (yield 84%). ^{1}H NMR (400 MHz, $CDCl_3$) δ 7.91 (d, *J* = 8.3 Hz, 2H), 7.81 (s, 1H), 7.57 – 7.39 (m, 4H), 7.32 (d, *J* = 14.2 Hz, 5H), 5.51 (d, *J* = 8.1 Hz, 1H), 5.27 (s, 1H), 5.15 – 4.99 (m, 2H), 4.84 – 4.60 (m, 1H), 4.05 – 3.15 (m, 10H), 1.47 – 1.36 (m, 9H). ^{13}C NMR (101 MHz, $CDCl_3$) δ 169.75, 156.77, 155.52, 133.66, 129.75, 128.65, 128.30, 127.41, 126.79, 125.32, 80.41, 67.05, 50.64, 46.69, 42.81, 41.64, 28.44. HRMS (ESI-QTOF) m/z [M + Na]+ calcd for $C_{31}H_{36}N_4O_6Na$ 583.2533; found 583.2544.

*Boc-*L*-Dap-Piperazine-Dansyl* (**60**). Compound **60** was synthesized according to the hydrogenolysis reaction detailed in *General Procedure R*. The product was carried forward without further purification or characterization.

*Boc-*L*-Dap-Piperazine-Sulfonyl-Naphthalene* (**61**). Compound **61** was synthesized according to the hydrogenolysis reaction detailed in *General Procedure R*. The product was carried forward without further purification or characterization.

Boc-L-*Dap-Piperazine-Naphthoyl* (**62**). Compound **62** was synthesized according to the hydrogenolysis reaction detailed in *General Procedure R*. The product was carried forward without further purification or characterization.

Boc-L-*Dap(Acryl)-Piperazine-Dansyl* (**63**). Compound **63** was synthesized according to the acrylation reaction provided in *General Procedure S* (yield 60%). ^{1}H NMR (400 MHz, $CDCl_3$) δ 8.59 (dt, *J* = 8.6, 1.1 Hz, 1H), 8.37 (dt, *J* = 8.7, 0.9 Hz, 1H), 8.21 (dd, *J* = 7.3, 1.3 Hz, 1H), 7.55 (ddd, *J* = 8.5, 7.5, 1.0 Hz, 2H), 7.21 (dd, *J* = 7.6, 0.9 Hz, 1H), 6.36 (s, 1H), 6.24 – 6.15 (m, 1H), 6.02 (dd, *J* = 17.0, 10.3 Hz, 1H), 5.61 (dd, *J* = 10.3, 1.4 Hz, 1H), 5.53 (d, *J* = 7.9 Hz, 1H), 4.66 (td, *J* = 7.8, 3.7 Hz, 1H), 3.83 – 3.50 (m, 4H), 3.37 – 3.11 (m, 1H), 2.91 (s, 6H), 1.37 (s, 9H). ^{13}C NMR (101 MHz, $CDCl_3$) δ 168.51, 166.28, 151.51, 132.50, 131.09, 131.00, 130.51, 130.41, 130.11, 128.43, 127.05, 123.48, 119.77, 115.67, 80.55, 50.29, 45.73, 45.60, 45.35, 45.26, 42.92, 41.97, 28.35. HRMS (ESI-QTOF) m/z [M + Na]+ calcd for $C_{27}H_{37}N_5O_6SNa$ 582.2362; found 582.2332.

Boc-L-*Dap(Acryl)-Piperazine-Sulfonyl-Naphthalene* (**64**). Compound **64** was synthesized according to the acrylation reaction provided in *General Procedure S* (yield 56%). ^{1}H NMR (600 MHz, $CDCl_3$) δ 8.69 (dd, *J* = 8.7, 1.1 Hz, 1H), 8.20 (dd, *J* = 7.4, 1.2 Hz, 1H), 8.08 (dt, *J* = 8.4, 1.1 Hz, 1H), 7.93 (dd, *J* = 8.2, 1.5 Hz, 1H), 7.70 – 7.52 (m, 3H), 6.48 – 6.36 (m, 1H), 6.17 (d, *J* = 17.0 Hz, 1H), 6.00 (dd, *J* = 17.0, 10.3 Hz, 1H), 5.62 – 5.55 (m, 2H), 4.65 (td, *J* = 7.9, 4.0 Hz, 1H), 3.73 – 3.48 (m, 5H), 3.35 – 3.09 (m, 5H), 1.34 (s, 9H). ^{13}C NMR (151 MHz, $CDCl_3$) δ 168.62, 166.22, 155.73, 135.02, 134.49, 132.13, 130.91, 130.47, 129.17, 128.90, 128.50, 127.14, 126.98, 124.91, 124.29, 80.43, 50.17, 45.70, 45.28, 42.63, 41.87, 28.30. HRMS (ESI-QTOF) m/z [M + Na]+ calcd for $C_{25}H_{32}N_4O_6SNa$ 539.1940; found 539.1948.

Boc-L-*Dap(Acryl)-Piperazne-Naphthoyl* (**65**). Compound **65** was synthesized according to the acrylation reaction provided in *General Procedure S* (yield 54%). ^{1}H NMR (600 MHz, $CDCl_3$) δ 7.92 – 7.76 (m, 3H), 7.59 – 7.37 (m, 4H), 6.49 (s, 1H), 6.27 – 6.14 (m, 1H), 6.10 – 5.96 (m, 1H), 5.73 – 5.55 (m, 2H), 4.81 – 4.60 (m, 1H), 4.06 – 3.13 (m, 10H), 1.48 – 1.31 (m, 9H). ^{13}C NMR (151 MHz, $CDCl_3$) δ 169.73, 168.68, 166.27, 155.81, 133.59, 133.41, 130.62, 130.44, 129.73, 129.53, 128.73, 127.34, 126.95, 126.76, 125.30, 124.45, 124.10, 80.51, 50.51, 47.19, 46.68, 46.06, 45.61, 42.81, 42.35, 41.80, 41.48, 28.40. HRMS (ESI-QTOF) m/z [M + Na]+ calcd for $C_{26}H_{32}N_4O_5Na$ 503.2270; found 503.2266.

H-L-*Dap(Acryl)-Piperazine-Dansyl* (**66**). Compound **66** was synthesized according to the acid catalyzed Boc deprotection detailed in *General Procedure P*. The product was carried forward without further purification or characterization.

H-L-*Dap(Acryl)-Piperazine-Sulfonyl-Naphthalene* (**67**). Compound **67** was synthesized according to the acid catalyzed Boc deprotection detailed in *General Procedure P*. The product was carried forward without further purification or characterization.

H-L-*Dap(Acryl)-Piperazine-Naphthoyl* (**68**). Compound **68** was synthesized according to the acid catalyzed Boc deprotection detailed in *General Procedure P*. The product was carried forward without further purification or characterization.

t-Butyl-Malonyl-L-*Dap(Acryl)-Piperazine-Dansyl* (**69**). Compound **69** was synthesized according to amide bond coupling reaction outlined in *General Procedure Q* (yield 24%). ^{1}H NMR (400 MHz, $CDCl_3$) δ 8.58 (dt, *J* = 8.6, 1.1 Hz, 1H), 8.33 (dt, *J* = 8.7, 0.9 Hz, 1H), 8.21 (dd, *J* = 7.4, 1.3 Hz, 1H), 7.62 (d, *J* = 7.6 Hz, 1H), 7.54 (ddd, *J* = 8.5, 7.4, 3.5 Hz, 2H), 7.19 (dd, *J* = 7.6, 0.9 Hz, 1H), 6.60 (t, *J* = 5.9 Hz, 1H), 6.19 (dd, *J* = 17.1, 1.4 Hz, 1H), 6.02 (dd, *J* = 17.1, 10.3 Hz, 1H), 5.60 (dd, *J* = 10.3, 1.4 Hz, 1H), 4.96 (td, *J* = 7.4, 3.4 Hz, 1H), 3.79 – 3.57 (m, 5H), 3.39 – 3.04 (m, 7H), 2.88 (s, 6H), 1.44 (s, 9H). ^{13}C NMR (101 MHz, $CDCl_3$) δ 168.21, 167.72, 166.29, 166.11, 152.02, 131.25, 130.95, 130.61, 130.24, 128.49, 126.87, 123.31, 119.40, 115.54, 83.06, 49.67, 45.65, 45.56, 45.33, 42.97, 42.55, 42.03, 28.11. HRMS (ESI-QTOF) m/z [M + Na]+ calcd for $C_{29}H_{39}N_5O_7SNa$ 624.2468; found 624.2469.

t-Butyl-Malonyl-L-*Dap(Acryl)-Piperazine-Naphthoyl* (**70**). Compound **70** was synthesized according to amide bond coupling reaction outlined in *General Procedure Q* (yield 42%). ^{1}H NMR (400 MHz, $CDCl_3$) δ 7.94 – 7.70 (m, 4H), 7.67 – 7.39 (m, 4H), 6.66 (s, 1H), 6.30 – 6.14 (m, 1H), 6.13 – 5.97 (m, 1H), 5.62 (dd, *J* = 15.4, 10.3 Hz, 1H), 5.12 – 4.91 (m, 1H), 4.28 – 3.17 (m, 12H), 1.47 (s, 9H). ^{13}C NMR (101 MHz, $CDCl_3$) δ 169.76, 168.30, 167.85, 166.41, 166.11, 133.64, 133.40, 130.71, 130.53, 129.78, 129.59, 128.77, 127.50, 126.90, 126.77, 125.30, 124.48, 124.16, 83.11, 50.03, 47.15, 46.64, 46.13, 45.63, 43.12, 42.45, 41.71, 41.44, 28.12. HRMS (ESI-QTOF) m/z [M + Na]+ calcd for $C_{28}H_{34}N_4O_6Na$ 545.2376; found 545.2351.

Malonyl-L-*Dap(Acryl)-Piperazine-Dansyl* (**71**). To a round bottom flask was added 0.028 g of the starting **69** (0.047 mmol, 1 eq) suspended in 2 mL toluene. To the flask was then added 0.284 g silica gel (10 × mass) and the reaction mixture was heated to 117 ˚C and stirred for 4 h. Upon completion the reaction mixture was diluted with 10% methanol in DCM and filtered through a pad a celite. The solution was then concentrated under reduced pressure and the resulting crude oil was purified via flash chromatography to yield 0.006 g (21%) of the product as a white yellow solid ^{1}H NMR (600 MHz, MeOD) δ 8.65 – 8.53 (m, 1H), 8.39 (dt, *J* = 8.7, 0.9 Hz, 1H), 8.21 (dd, *J* = 7.3, 1.3 Hz, 1H), 7.61 (ddd, *J* = 16.2, 8.6, 7.4 Hz, 2H), 7.29 (dd, *J* = 7.6, 0.9 Hz, 1H), 6.25 – 6.04 (m, 2H), 5.57 (dd, *J* = 9.5, 2.5 Hz, 1H), 4.99 (dd, *J* = 7.9, 4.5 Hz, 1H), 3.72 – 3.51 (m, 5H), 3.30 – 3.10 (m, 5H), 2.88 (s, 6H), 2.66 (s, 2H). ^{13}C NMR (151 MHz, MeOD) δ 174.89, 171.62, 169.87, 168.63, 153.26, 134.03, 132.02, 131.88, 131.82, 131.56, 131.43, 129.33, 126.86, 124.40, 120.75, 116.65, 107.10, 50.20, 49.57, 46.85, 46.41, 45.78, 42.88, 42.14, 40.41. HRMS (ESI-QTOF) m/z [M + Na]+ calcd for $C_{25}H_{31}N_5O_7SNa$ 568.1842; found 568.1822.

Malonyl-L-*Dap(Acryl)-Piperazine-Naphthoyl* (**72**). Compound **72** was synthesized according to *General Procedure P* and purified via flash chromatography (yield 30%). ^{1}H NMR (400 MHz, MeOD) δ 8.01 – 7.89 (m, 2H), 7.83 (t, *J* = 8.8 Hz, 1H), 7.64 – 7.45 (m, 4H), 6.26 – 6.12 (m, 2H), 5.71 – 5.58 (m, 1H), 5.22 – 4.99 (m, 1H), 4.21 – 3.04 (m, 12H). ^{13}C NMR (101 MHz, MeOD) δ 171.78, 171.66, 169.91, 168.97, 168.72, 134.96, 134.56, 131.78, 131.67, 130.77, 130.69, 129.70, 128.47, 128.44, 127.80, 127.18, 126.36, 125.47, 125.23, 50.44, 47.82, 46.93, 46.39, 43.59, 43.06, 43.01, 42.56, 41.98. HRMS (ESI-QTOF) m/z [M + Na]+ calcd for $C_{24}H_{26}N_4O_6Na$ 489.1750; found 489.1767.

Methyl-Ester-Cyclopropyl-L-*Dap(Acryl)-Piperazine-Dansyl* (**73**). Compound **73** was synthesized according to the amide bond coupling reaction detailed in *General Procedure Q* (yield 85%). ^{1}H NMR (400 MHz, $CDCl_3$) δ 9.52 (d, *J* = 7.4 Hz, 1H), 8.58 (d, *J* = 8.5 Hz, 1H), 8.34 (d, *J* = 8.7 Hz,

1H), 8.24 – 8.13 (m, 1H), 7.62 – 7.45 (m, 2H), 7.19 (d, *J* = 7.6 Hz, 1H), 6.53 (t, *J* = 5.8 Hz, 1H), 6.18 (dd, *J* = 17.0, 1.5 Hz, 1H), 6.02 (dd, *J* = 17.0, 10.2 Hz, 1H), 5.58 (dd, *J* = 10.2, 1.5 Hz, 1H), 4.95 (td, *J* = 7.2, 3.7 Hz, 1H), 3.85 – 3.51 (m, 8H), 3.40 – 3.08 (m, 6H), 2.89 (s, 6H), 1.55 (tdd, *J* = 18.8, 9.3, 5.5 Hz, 4H). ^{13}C NMR (101 MHz, $CDCl_3$) δ 173.34, 169.41, 168.24, 166.10, 132.51, 131.05, 130.69, 130.39, 128.38, 126.84, 123.56, 115.77, 52.58, 49.79, 45.70, 45.36, 45.28, 42.78, 41.97, 26.34, 20.51, 20.37. HRMS (ESI-QTOF) m/z [M + Na]+ calcd for $C_{28}H_{35}N_5O_7SNa$ 608.2155; found 608.2166.

*Acid-Cyclopropyl-*L*-Dap(Acryl)-Piperazine-Dansyl* (**74**). Compound **74** was synthesized according to the hydrolysis reaction in *General Procedure T* (yield 50%). ^{1}H NMR (600 MHz, MeOD) δ 8.62 (dt, *J* = 8.6, 1.1 Hz, 1H), 8.39 (dt, *J* = 8.7, 0.9 Hz, 1H), 8.20 (dd, *J* = 7.3, 1.3 Hz, 1H), 7.60 (ddd, *J* = 17.4, 8.6, 7.5 Hz, 2H), 7.28 (dd, *J* = 7.6, 0.9 Hz, 1H), 6.17 – 6.04 (m, 2H), 5.56 (dd, *J* = 7.0, 5.0 Hz, 1H), 5.01 (dd, *J* = 7.5, 5.1 Hz, 1H), 3.74 – 3.60 (m, 3H), 3.58 – 3.50 (m, 2H), 3.35 (dd, *J* = 13.7, 7.5 Hz, 1H), 3.30 – 3.12 (m, 4H), 2.88 (s, 6H), 1.33 – 1.26 (m, 2H), 1.23 – 1.19 (m, 1H), 1.16 – 1.11 (m, 1H). ^{13}C NMR (151 MHz, MeOD) δ 178.43, 175.05, 170.39, 168.58, 153.26, 134.02, 132.02, 131.77, 131.70, 131.56, 131.42, 129.34, 127.00, 124.41, 120.71, 116.64, 50.16, 46.89, 46.53, 46.48, 45.78, 42.92, 42.28, 28.42, 19.18, 18.99. HRMS (ESI-QTOF) m/z [M + Na]+ calcd for $C_{27}H_{32}N_5O_7SNa_2$ 616.1818; found 616.1846.

*Succinyl-*L*-Dap(Acryl)-Piperazine-Dansyl* (**75**). Compound **75** was synthesized according to *General Procedure U* with succinic anhydride (yield 83%). ^{1}H NMR (400 MHz, MeOD) δ 8.65 – 8.58 (m, 1H), 8.39 (d, *J* = 8.7 Hz, 1H), 8.20 (dd, *J* = 7.4, 1.3 Hz, 1H), 7.60 (ddd, *J* = 10.9, 8.6, 7.4 Hz, 2H), 7.27 (d, *J* = 7.5 Hz, 1H), 6.12 (d, *J* = 6.0 Hz, 2H), 5.56 (t, *J* = 6.0 Hz, 1H), 4.96 (dd, *J* = 7.5, 5.1 Hz, 1H), 3.71 – 3.49 (m, 5H), 3.35 (d, *J* = 2.7 Hz, 1H), 3.30 – 3.07 (m, 4H), 2.87 (s, 6H), 2.55 – 2.32 (m, 4H). ^{13}C NMR (101 MHz, MeOD) δ 174.41, 170.08, 168.57, 153.24, 133.93, 132.02, 131.85, 131.70, 131.56, 131.39, 129.32, 127.05, 124.40, 120.73, 116.62, 49.97, 46.95, 46.47, 46.43, 45.77, 42.91, 41.92, 31.65, 30.71. HRMS (ESI-QTOF) m/z [M + Na]+ calcd for $C_{26}H_{33}N_5O_7SNa$ 582.1998; found 582.1996.

*Succinyl-*L*-Dap(Acryl)-Piperazine-Sulfonyl-Naphthalene* (**76**). Compound **76** was synthesized according to *General Procedure U* with succinic anhydride (yield 24%). ^{1}H NMR (600 MHz, MeOD) δ 8.74 (dq, *J* = 8.7, 0.9 Hz, 1H), 8.20 (td, *J* = 7.4, 1.1 Hz, 2H), 8.02 (ddt, *J* = 8.1, 1.3, 0.6 Hz, 1H), 7.69 (ddd, *J* = 8.6, 6.9, 1.4 Hz, 1H), 7.66 (s, 2H), 6.15 – 6.05 (m, 2H), 5.60 – 5.53 (m, 1H), 4.96 (dd, *J* = 7.5, 5.2 Hz, 1H), 3.70 – 3.57 (m, 3H), 3.57 – 3.47 (m, 2H), 3.33 (d, *J* = 7.5 Hz, 1H), 3.29 – 3.07 (m, 4H), 2.55 – 2.50 (m, 2H), 2.46 – 2.32 (m, 2H). ^{13}C NMR (151 MHz, MeOD) δ 176.37, 174.21, 170.08, 168.55, 136.03, 135.94, 133.56, 131.92, 131.64, 130.22, 130.09, 129.30, 128.13, 127.11, 126.10, 125.44, 49.92, 46.98, 46.50, 46.41, 42.88, 41.89, 31.29, 30.11. HRMS (ESI-QTOF) m/z [M + Na]+ calcd for $C_{24}H_{28}N_4O_7SNa$ 539.1576; found 539.1563.

*Succinyl-*L*-Dap(Acryl)-Piperazine-Naphthoyl* (**77**). Compound **77** was synthesized according to *General Procedure U* with succinic anhydride (yield 11%). ^{1}H NMR (600 MHz, MeOD) δ 8.01 – 7.90 (m, 2H), 7.83 (t, *J* = 8.0 Hz, 1H), 7.63 – 7.47 (m, 4H), 6.30 – 6.10 (m, 2H), 5.72 – 5.58 (m, 1H), 5.17 – 4.95 (m, 1H), 4.16 – 3.36 (m, 9H), 3.28 – 3.15 (m, 1H), 2.63 – 2.38 (m, 4H). ^{13}C NMR (151 MHz, MeOD) δ 177.07, 174.59, 171.81, 170.23, 168.67, 134.98, 134.58, 131.82, 131.70, 130.77, 130.71, 129.70, 129.55, 128.43, 127.80, 127.13, 126.36, 125.49, 125.21, 125.10, 50.32,

48.39, 47.87, 46.93, 46.37, 43.59, 43.09, 43.00, 42.59, 41.99, 31.77, 30.84. HRMS (ESI-QTOF) m/z [M + Na]+ calcd for $C_{25}H_{28}N_4O_6Na$ 503.1907; found 503.1898.

*Methyl-Ester-Sulfonyl-*L*-Dap(Acryl)-Piperazine-Dansyl* (**78**). Compound **78** was synthesized via a sulfonylation reaction. To a round bottom flask was added 0.091 g of the free amine starting material **66** (0.198 mmol, 1 eq) along with 0.030 mL triethylamine (0.218 mmol, 1.1 eq) solubilized in 2 mL DCM. To the flask was subsequently added 0.037 g methyl (chlorosulfonyl)acetate (0.218 mmol, 1.1 eq) and the reaction mixture was stirred at room temperature for 1.5 h. Upon completion the reaction mixture was washed three times with a saturated solution of $NaHCO_3$ and brine. The organic layer was dried over $MgSO_4$, filtered, and concentrated under reduced pressure. The resulting crude oil was purified via flash chromatography to obtain 0.059 g (50%) of product **78** and carried forward to the hydrolysis reaction without further characterization.

*Acid-Sulfonyl-*L*-Dap(Acryl)-Piperazine-Dansyl* (**79**). Compound **79** was synthesized according to *General Procedure T* (yield 83%). ^{1}H NMR (400 MHz, MeOD) δ 8.61 (dt, *J* = 8.6, 1.1 Hz, 1H), 8.37 (d, *J* = 8.7 Hz, 1H), 8.20 (dd, *J* = 7.4, 1.3 Hz, 1H), 7.60 (ddd, *J* = 9.9, 8.7, 7.5 Hz, 2H), 7.27 (dd, *J* = 7.6, 0.9 Hz, 1H), 6.23 – 6.07 (m, 2H), 5.57 (dd, *J* = 8.9, 3.0 Hz, 1H), 4.69 (dd, *J* = 7.8, 4.8 Hz, 1H), 3.83 (s, 2H), 3.72 (t, *J* = 5.2 Hz, 2H), 3.60 (t, *J* = 5.2 Hz, 2H), 3.50 (dd, *J* = 13.7, 4.7 Hz, 1H), 3.35 (s, 1H), 3.31 – 3.15 (m, 4H), 2.87 (s, 6H). ^{13}C NMR (101 MHz, MeOD) δ 170.46, 169.38, 168.57, 153.19, 134.05, 131.99, 131.71, 131.62, 131.45, 131.34, 129.34, 127.23, 124.39, 120.63, 116.61, 53.90, 46.74, 46.58, 46.30, 45.77, 43.13. HRMS (ESI-QTOF) m/z [M + Na]+ calcd for $C_{24}H_{30}N_5O_8S_2Na_2$ 626.1331; found 626.1343.

*Cbz-*D*-Dap(Boc)-Piperazine-Naphthoyl* (**80**). Compound **80** was synthesized according to the amide bond coupling reaction outlined in *General Procedure Q* (yield 74%). ^{1}H NMR (400 MHz, $CDCl_3$) δ 7.96 – 7.76 (m, 3H), 7.57 – 7.38 (m, 4H), 7.37 – 7.26 (m, 5H), 6.06 – 5.85 (m, 1H), 5.14 – 4.97 (m, 3H), 4.89 – 4.63 (m, 1H), 4.20 – 3.12 (m, 10H), 1.47 – 1.30 (m, 9H). ^{13}C NMR (101 MHz, $CDCl_3$) δ 169.72, 168.56, 156.14, 156.07, 136.25, 133.62, 129.70, 128.73, 128.65, 128.36, 128.25, 128.19, 127.37, 126.76, 125.30, 124.56, 124.08, 79.95, 67.15, 51.30, 47.14, 46.59, 46.05, 45.59, 43.09, 42.79, 42.34, 41.76, 41.41, 28.40. HRMS (ESI-QTOF) m/z [M + Na]+ calcd for $C_{31}H_{36}N_4O_6Na$ 583.2533; found 583.2540.

*H-*D*-Dap(Boc)-Piperazine-Naphthoyl* **(81)**. Compound **81** was synthesized according to the hydrogenolysis reaction detailed in *General Procedure R*. The product was carried forward without further purification or characterization.

*Acryl-*D*-Dap(Boc)-Piperazine-Naphthoyl* (**82**). To a round bottom flask was added 2.49 g of the starting amine **81** (5.842 mmol, 1 eq), 2.035 mL DIPEA (11.684 mmol, 2 eq), and 0.071 g DMAP (0.584 mmol, 0.1 eq) solubilized in 60 mL DCM at 0 ˚C. To the flask was then added 0.563 mL acryloyl chloride (7.010 mmol, 1.2 eq) dropwise. The reaction was allowed to warm to room temperature and stirred for 2 h. Upon completion, the reaction mixture was washed with water, and three times with a saturated solution of $NaHCO_3$. The organic phase was dried with $MgSO_4$, filtered, and concentrated under reduced pressure. The resulting crude oil was purified via flash chromatography to yield 1.730 g (62%) of the product **82** as a white solid. ^{1}H NMR (400 MHz, $CDCl_3$) δ 7.96 – 7.76 (m, 3H), 7.59 – 7.38 (m, 4H), 6.92 (s, 1H), 6.38 – 6.05 (m, 2H), 5.71 – 5.61

(m, 1H), 5.16 – 4.92 (m, 2H), 4.28 – 3.65 (m, 4H), 3.62 – 3.13 (m, 6H), 1.48 – 1.34 (m, 9H). ^{13}C NMR (101 MHz, $CDCl_3$) δ 169.76, 168.47, 165.36, 156.21, 133.63, 133.48, 130.37, 129.75, 129.64, 128.74, 127.53, 127.41, 126.77, 125.31, 124.59, 124.10, 80.03, 50.21, 47.08, 46.57, 46.10, 45.63, 42.87, 42.42, 41.71, 41.36, 28.42. HRMS (ESI-QTOF) m/z [M + Na]+ calcd for $C_{26}H_{32}N_4O_5Na$ 503.2270; found 503.2256.

*TFA.Acryl-*D*-Dap-Piperazine-Naphthoyl* (**83**). Compound **83** was synthesized according to the Boc deprotection reaction outlined in *General Procedure P*, excluding the $NaHCO_3$ washes and carried forward as the TFA salt in quantitate yield after concentrating the reaction mixture.
*Acryl-*D*-Dap(Succinyl)-Piperazine-Naphthoyl* (**84**). Compound **84** was synthesized according to *General Procedure U* with succinic anhydride (yield 83%). ^{1}H NMR (400 MHz, MeOD) δ 8.03 – 7.77 (m, 3H), 7.62 – 7.45 (m, 4H), 6.39 – 6.13 (m, 2H), 5.75 – 5.62 (m, 1H), 5.24 – 4.98 (m, 1H), 4.14 – 3.34 (m, 9H), 3.29 – 3.12 (m, 1H), 2.58 – 2.37 (m, 4H). ^{13}C NMR (101 MHz, MeOD) δ 177.25, 175.64, 171.80, 170.32, 167.72, 134.95, 134.59, 131.57, 130.77, 130.70, 129.70, 128.46, 127.80, 127.62, 126.37, 125.50, 125.23, 52.13, 50.45, 47.85, 46.99, 46.45, 43.64, 43.04, 42.59, 41.88, 31.99, 31.92, 31.07, 30.65, 30.23. HRMS (ESI-QTOF) m/z [M + Na]+ calcd for $C_{25}H_{28}N_4O_6Na$ 503.1907; found 503.1897.

Boc-Glu(MA)-Piperazine-Naphthoyl (**85**). Compound **85** was synthesized via an amide bond coupling reaction. To a round bottom flask was added 0.737 g of compound **28** (2.567 mmol, 1 eq) with 1.168 g HBTU (3.080 mmol, 1.2 eq) and 1.733 mL DIPEA (10.267 mmol, 4 eq) solubilized in 7 DMF at 0 ˚C. The activation of the acid was allowed to occur for 0.5 h upon which 1.091 g of the corresponding TFA salt **56*** (3.080 mmol, 1.2 eq) was added as a solution in 2 mL DMF with 0.433 mL DIPEA (2.567 mmol, 1 eq). The reaction was allowed to stir overnight at room temperature. Upon completion, the reaction mixture was diluted with ethyl acetate and washed with 1 M HCl, water, a saturated solution of $NaHCO_3$, and brine. The organic layer was dried over $MgSO_4$, filtered, and concentrated under reduced pressure. The resulting crude oil was purified via flash chromatography to yield 1.000 g (77%) of the product as a white solid. ^{1}H NMR (400 MHz, $CDCl_3$) δ 8.00 – 7.72 (m, 3H), 7.64 – 7.35 (m, 4H), 7.01 – 6.79 (m, 1H), 5.91 – 5.75 (m, 1H), 5.35 (d, J = 8.6 Hz, 1H), 4.79 – 4.38 (m, 1H), 4.20 – 3.11 (m, 11H), 2.39 – 2.14 (m, 2H), 1.93 – 1.56 (m, 2H), 1.53 – 1.32 (m, 9H). ^{13}C NMR (101 MHz, $CDCl_3$) δ 170.59, 169.74, 166.81, 155.46, 147.49, 133.62, 133.40, 129.74, 128.74, 127.40, 126.76, 125.29, 124.52, 124.08, 122.06, 80.18, 51.61, 49.56, 47.32, 46.89, 45.81, 45.62, 42.72, 42.26, 42.02, 41.64, 31.89, 28.42, 28.00. HRMS (ESI-QTOF) m/z [M + Na]+ calcd for $C_{28}H_{35}N_3O_6Na$ 532.2424; found 532.2449.

TFA.H-Glu(MA)-Piperazine-Naphthoyl (**86**). Compound **86** was synthesized according to *General Procedure P*, excluding the $NaHCO_3$ washes and carried forward as the TFA salt in quantitate yield after concentrating the reaction mixture.

Succinyl-Glu(MA)-Piperazine-Naphthoyl (**87**). Compound **87** was synthesized according to *General Procedure U* with succinic anhydride, DIPEA as the base, and DMF as the solvent (yield 67%). ^{1}H NMR (400 MHz, MeOD) δ 8.01 – 7.77 (m, 3H), 7.63 – 7.46 (m, 4H), 7.03 – 6.84 (m, 1H), 5.95 – 5.80 (m, 1H), 4.72 (dd, J = 8.6, 5.3 Hz, 1H), 4.17 – 3.43 (m, 9H), 3.30 – 3.20 (m, 2H), 2.65 – 2.60 (m, 1H), 2.57 – 2.43 (m, 3H), 2.34 – 2.18 (m, 2H), 1.97 – 1.72 (m, 2H). ^{13}C NMR (101 MHz, MeOD) δ 176.07, 174.15, 172.06, 171.74, 168.52, 149.58, 134.94, 134.58, 130.75, 129.69, 128.44, 127.78, 126.35, 125.49, 125.22, 122.62, 52.18, 51.95, 49.85, 47.91, 46.97, 46.39, 43.58,

43.14, 42.99, 42.66, 31.41, 31.23, 30.04, 29.79, 29.25. HRMS (ESI-QTOF) m/z [M + Na]+ calcd for $C_{27}H_{31}N_3O_7Na$ 532.2060; found 532.2076.

Phthalyl-Glu(MA)-Piperazine-Naphthoyl (**88**). Compound **88** was synthesized according to *General Procedure U* with phthalic anhydride, DIPEA as the base, and DMF as the solvent (yield 59%). ^{1}H NMR (400 MHz, MeOD) δ 8.04 – 7.92 (m, 2H), 7.91 – 7.76 (m, 2H), 7.65 – 7.33 (m, 6H), 7.24 – 7.09 (m, 1H), 7.05 – 6.88 (m, 1H), 5.99 – 5.83 (m, 1H), 5.08 (s, 1H), 4.27 – 3.33 (m, 8H), 3.36 – 3.08 (m, 3H), 2.32 (s, 2H), 2.04 – 1.78 (m, 2H). ^{13}C NMR (101 MHz, MeOD) δ 175.26, 171.74, 168.56, 168.53, 164.82, 149.66, 138.89, 138.51, 134.96, 134.60, 132.24, 130.93, 130.76, 129.91, 129.70, 129.20, 128.93, 128.45, 127.79, 126.36, 126.29, 125.50, 125.23, 122.71, 122.66, 51.97, 50.36, 47.92, 46.63, 43.77, 43.14, 42.68, 36.93, 31.64, 31.18, 30.90, 29.43. HRMS (ESI-QTOF) m/z [M + Na]+ calcd for $C_{31}H_{31}N_3O_7Na$ 580.2060; found 580.2090.

Fmoc-6AH-OH ***89***. Commercially available 6-aminohexanoic acid (1.000 g, 7.62 mmol, 1 eq) and $Na_2CO_{3(s)}$ (3 eq) were added to a round-bottom flask and dissolved in a 1:1 mixture of H_2O/dioxane (0.1 M). The flask was cooled to 0 °C, stirring was commenced, and fluorenylmethyloxycarbonyl chloride (1 eq) was added in 1 portion. The reaction mixture was stirred at 0 °C for 10 min, and was then allowed to warm to room temperature where it was stirred for another 6 h. Upon completion, dioxane was removed through rotary evaporation and the resulting aqueous solution was washed with Et_2O. The recovered aqueous layer was subsequently acidified to around pH 2 through the addition of 1 M $HCl_{(aq)}$ and extracted thrice into EtOAc. The combined organics were dried over $MgSO_4$, filtered, and concentrated under reduced pressure to yield the desired product as a white solid (2.500 g, 7.09 mmol, 93% yield). Characterization matches previous reports [77].
*Fmoc-*D*-Asp(OtBu)-CTC* ***90***. Compound **90** was prepared from fresh 2-chlorotrityl chloride resin (100-200 mesh, 1.00 g, 0.75 mmol, 1 eq) and Fmoc-D-Asp(OtBu)-OH **9** (1.2 eq) through *General Procedure D*. The resin loading was estimated as 0.500 mmol/g, and 1.00 g of loaded resin was recovered (0.50 mmol, 67% yield).

*RhB-Pro-6AH-*D*-Asp(OtBu)-OH* ***91***. Manually-loaded Fmoc-D-Asp(OtBu)-CTC resin **90** (0.5 mmol, 1 eq) was placed into the automated peptide synthesizer and swelled in 20 mL DMF for 5 min. Fmoc-6AH-OH **89** (4 eq) and Fmoc-Pro-OH (4 eq) were then coupled through cycles of standard deprotections (10 mL of 20% v/v piperidine in DMF at 90 °C for 1 min) and couplings (4 eq HATU, 8 eq DIPEA in 16 mL 7:1 DMF/NMP solution at 50 °C over 10 min). The resin was washed 3 times with 7-10 mL of DMF between each step. After deprotection of the Pro residue's Fmoc group, the resin was removed from the instrument. Rhodamine B was double-coupled manually (5 eq rhodamine B, 5 eq HATU, 10 eq DIPEA, 10 mL DMF, two cycles of 40 min each at room temperature). The resin was then washed with DMF and DCM. The peptide was cleaved from the resin through two 30 min treatments with 20% HFIP in DCM. The crude mixture was concentrated through rotary evaporation, precipitated in cold ether, and pelleted through centrifugation. Discarding of the supernatant and extensive drying under vacuum produced the desired peptide as a red solid (282 mg, 0.34 mmol, 68% yield), which was carried forward without further purification. LC-MS *m/z* 825 for $[M]^+$.

*RhB-Pro-6AH-*D*-Asp(OtBu)-Glu(MA)-Nle-Nle-Leu-Pro-Trp-Pro-OH (**51**).* Compound **92** was prepared through *General Procedure M* using TFA.H-Glu(MA)-Nle-Nle-Leu-Pro-Trp-Pro-OH **40** (347 mg, 0.34 mmol, 1 eq) and RhB-Pro-6AH-D-Asp(OtBu)-OH **91** (282 mg, 0.34 mmol, 1 eq),

both prepared herein. The reaction flask was covered with aluminum foil to prevent degradation of the fluorophore from exposure to light. The desired peptide was obtained as a deep purple solid (85.7 mg, 0.050 mmol, 15% yield). LC-MS *m/z* 1714 for $[M]^+$.

*RhB-Pro-6AH-*D*-Asp-Glu(MA)-Nle-Nle-Leu-Pro-Trp-Pro-OH* ***(93, KM93)***. Compound **93** (**KM93**) was prepared through *General Procedure N* from RhB-Pro-6AH-D-Asp(OtBu)-Glu(MA)-Nle-Nle-Leu-Pro-Trp-Pro-OH **92** (85.7 mg, 0.050 mmol, 1 eq). The reaction flask was covered with aluminum foil to prevent degradation of the fluorophore from exposure to light. The desired peptide was obtained as a bright magenta solid (23.6 mg, 0.014 mmol, 28% yield). HRMS (ESI-QTOF) *m/z* $[M]^+$ calcd for $C_{90}H_{122}N_{13}O_{17}$ 1656.9082; found 1656.9102.

Supplementary Kinetic Traces and Inhibition Experiments

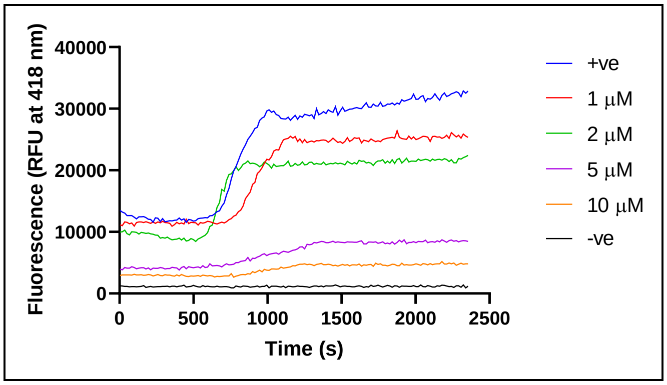

Figure S1. *Fluorescence-time curve of the spike experiment performed with inhibitor* ***47*** *to confirm the reversibility of FXIIIa inhibition. Additional A101 substrate was added to the reactions corresponding to the positive control with no inhibitor (+ve), the negative control with no enzyme (-ve), and all 4 inhibitor concentrations (1, 2, 5, and 10 µM) after the initial plateaus in fluorescence had been reached. Fluorescence emission (RFU, relative fluorescence unit emission at 418 nm after excitation at 313 nm) was then monitored over time.*

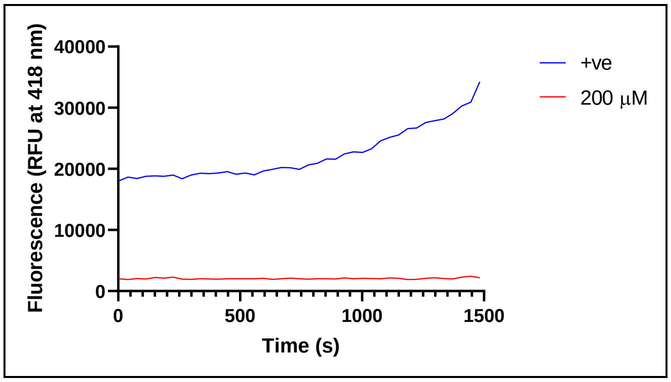

Figure S2. *Fluorescence-time curve of the spike experiment performed with inhibitor* ***23*** *to confirm the irreversibility of FXIIIa inhibition. Additional A101 substrate was added to the reactions corresponding to the positive control with no inhibitor (+ve) and the highest inhibitor concentration (200 µM) after the initial plateaus in fluorescence had been reached. Fluorescence emission (RFU, relative fluorescence unit emission at 418 nm after excitation at 313 nm) was then monitored over time.*

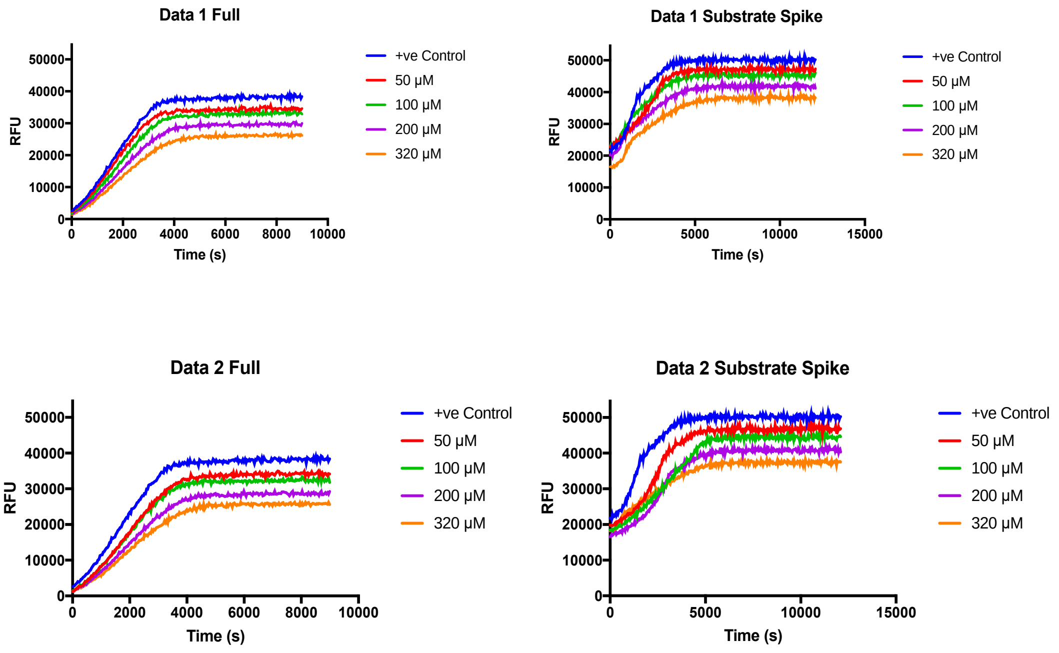

Figure S3. *Substrate spike experiment with small molecule inhibitor **79** and FXIIIa. Upon completion of the activity assay, another 100 μM of A101 was added and the RFU increased, implying FXIIIa was still active (top and bottom left panels).*

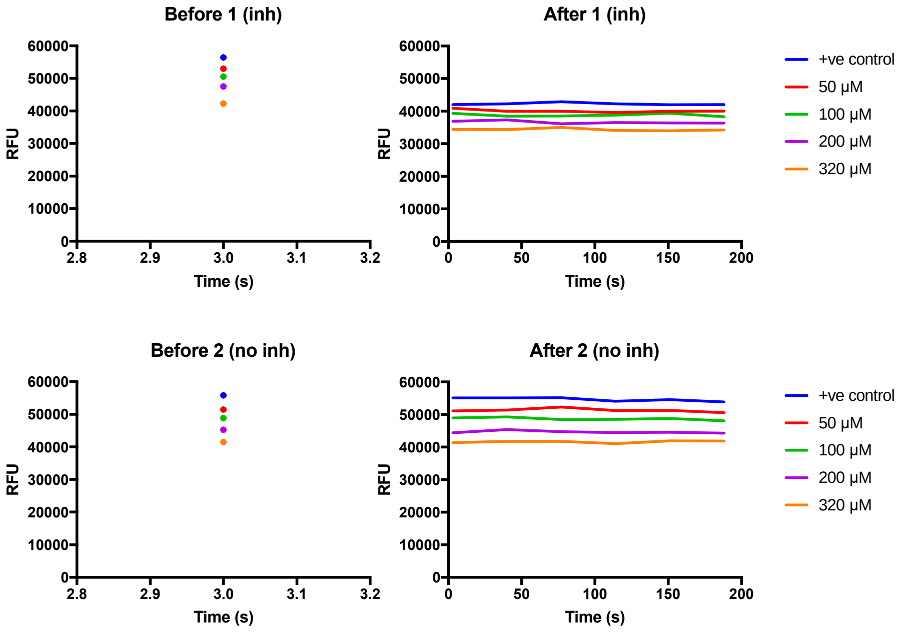

Figure S4. *Inhibitor spike experiment with small molecule inhibitor **79**. Upon completion of the activity assay another dose of 320 μM **79** was added and the RFU plateau at a lower value (top right panel).*

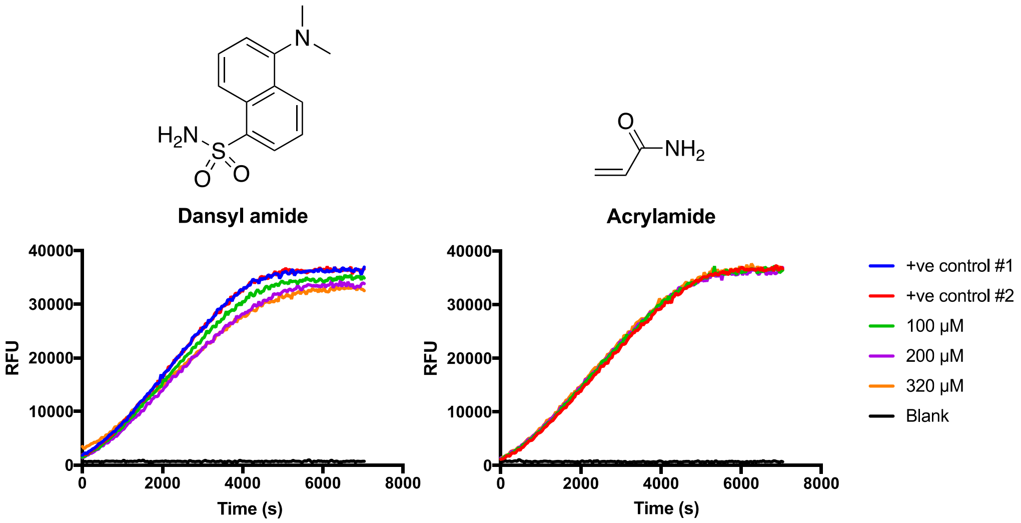

Figure S5. *Inhibition experiment of FXIIIa with small molecule inhibitor scaffold components dansyl amide and acrylamide assayed at four different concentrations (0, 100, 200, 320 µM).*

SDS-PAGE of Fluorescent Labelling Experiment

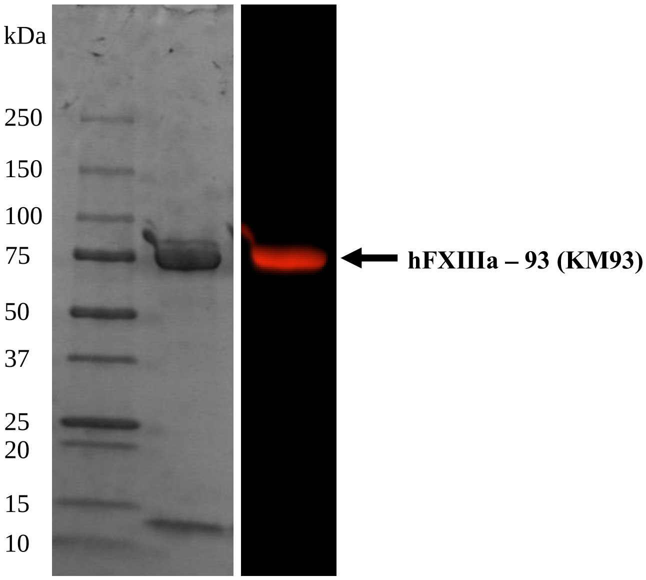

Figure S6. *SDS-PAGE of commercially available FXIIIa incubated with 30 µM fluorescent probe **93** (**KM93**) visualized first for fluorescence and then using Coomassie Blue staining.*

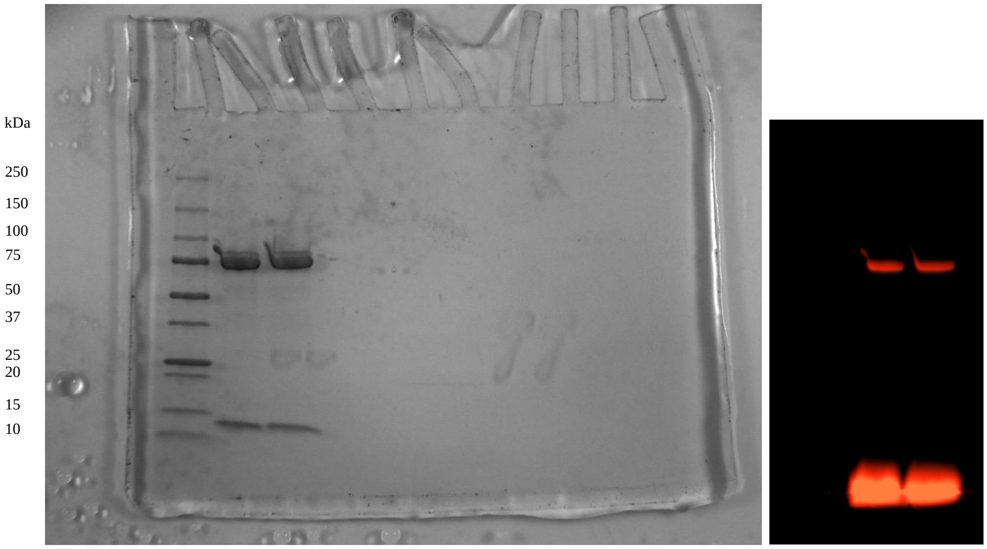

Figure S7. *Full SDS-PAGE gel of fluorescent labelling of commercially available FXIIIa with 30 µM **93 (KM93)**. Note the excess labelling agent at the very bottom edge of the gel does not correspond to protein.*

Supplementary Cellular Labelling Figures

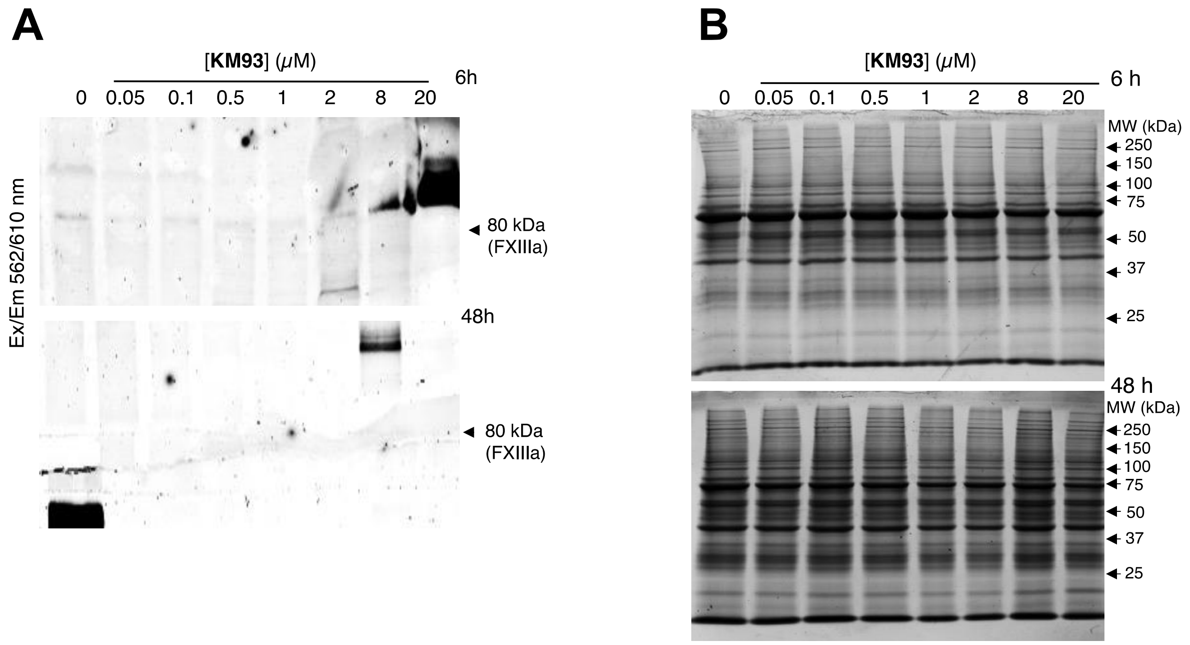

Figure S8. A) Fluorescent labelling of FXIIIa in murine bone marrow macrophages (BMM). BMMs were incubated with 0-20 µM **KM93** for 6 h (top gel) or for 48 h (bottom gel), lysed, and protein extracts were prepared and resolved with denaturing, 10% SDS-PAGE. The gels were visualized using a fluoroimager (Ex/Em 562/610). The gels show a dose dependent labelling of a ~80 kDa band, which corresponds to a molecular weight of FXIIIa, consistent with the red fluorescence of the rhodamine moiety of **KM93**. The identity of the band at ~100 kDa is unknown but it is also present in the negative control and thus represents background. **B)** Coomassie Blue stained gels confirm equal loading in each lane.

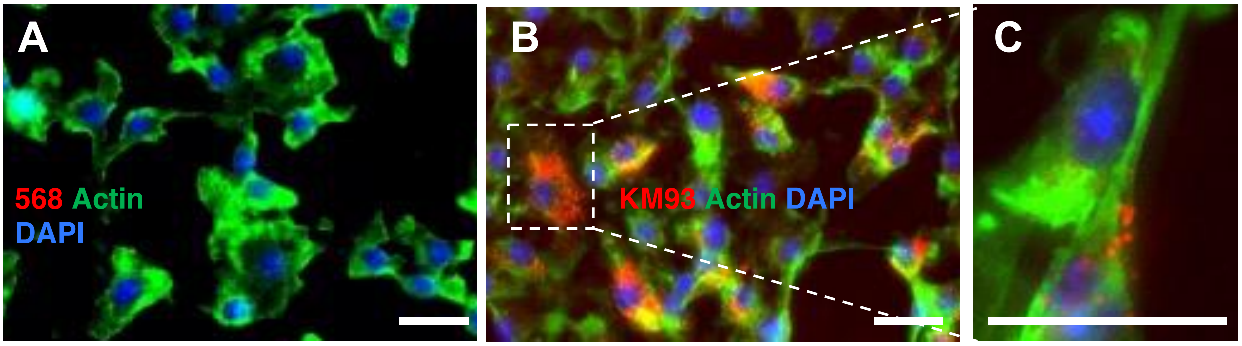

Figure S9. Fluorescence visualization of **KM93** in murine bone marrow macrophages (BMM). Cells were incubated with 20 µM **KM93** for 48 h in microscopy chamber slides. At end point, cells were fixed, cytoskeletal actin was stained with AlexaFluor®-488-phalloidin (green) and nuclei were stained with DAPI (blue). **KM93** was visualized in the 568-nm channel (red). **A)**: Cells that were incubated in the absence of the probe showed no signal in the 568-nm channel (negative control). **B)**: Strong red fluorescence was seen observed after 48 h incubation with 20 µM **KM93**. The probe was confirmed to be intracellular by actin staining (see inset **C**). White magnification bar represents 20 µm.

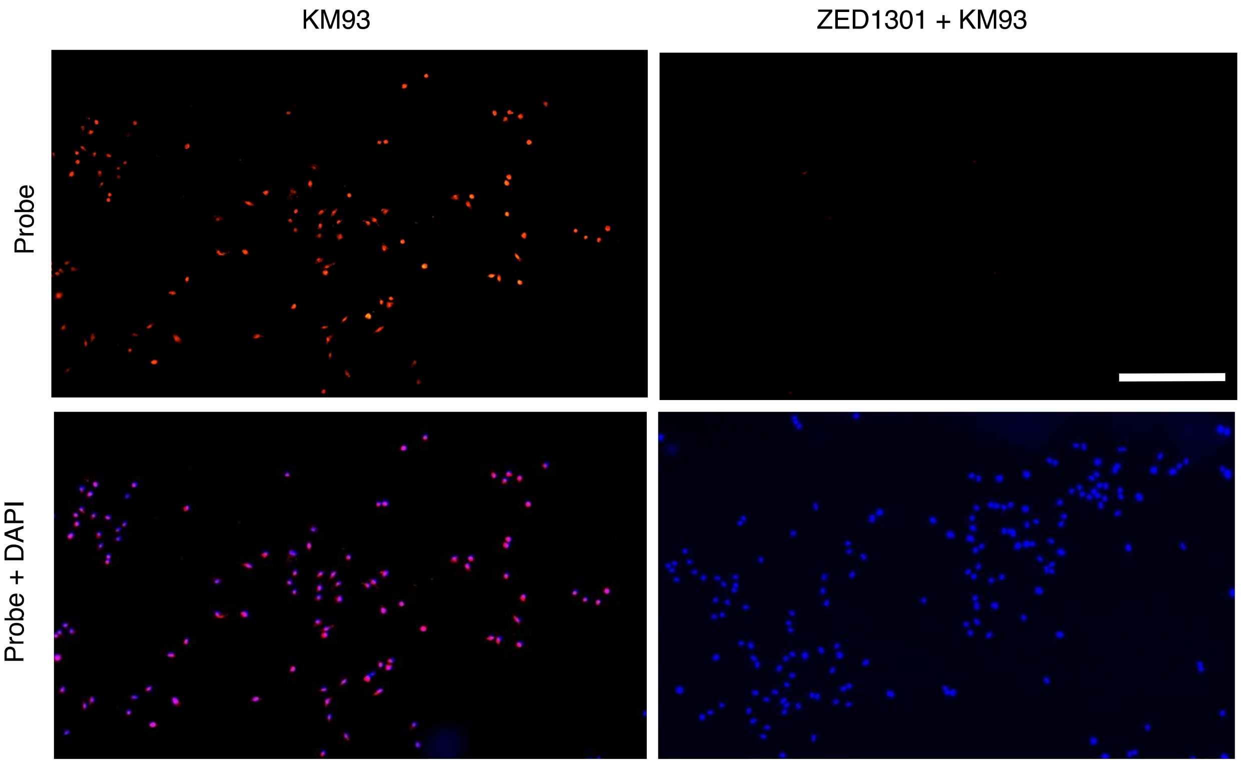

Figure S10. FXIIIa inhibitor ZED1301 is able to block labelling by KM93 probe in BMM cells. BMM cells were preincubated in the absence or presence of 20 µM ZED1301 for 2 hours to inhibit FXIIIa, prior to addition of 20 µM **KM93** and further incubation for 4 hours. Cells were fixed and washed, nuclei were stained with DAPI (blue) and incorporation of the red probe **KM93** was observed by fluorescence microscopy. Negligible red fluorescence was observed in cells blocked by pre-incubation with ZED1301. Magnification bar equals 40 µm.

Kinetic Fitting for Inhibitors

Inhibitor	FXIIIa	TG2
**11**	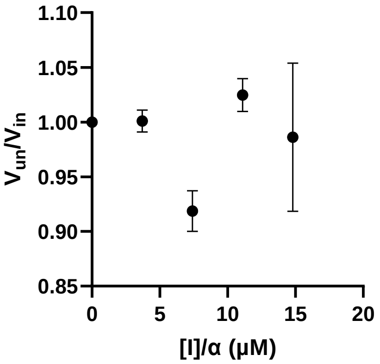<br>No inhibition	No inhibition
**12**	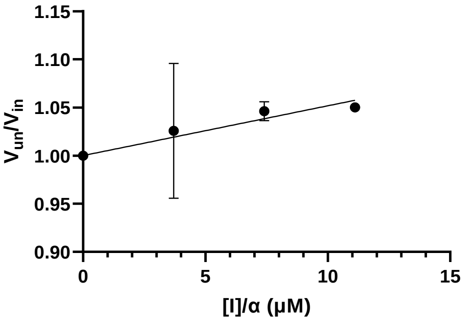<br>K_i = 193 ± 20 μM	No inhibition
**13**	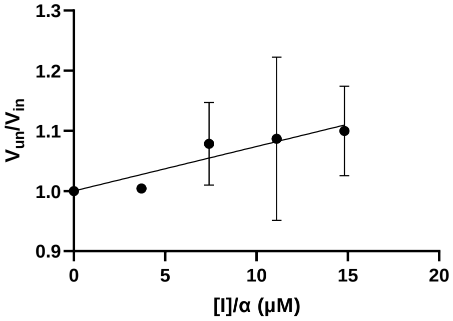<br>K_i = 135 ± 16 μM	No inhibition

<table>
<tr>
<td>14</td>
<td>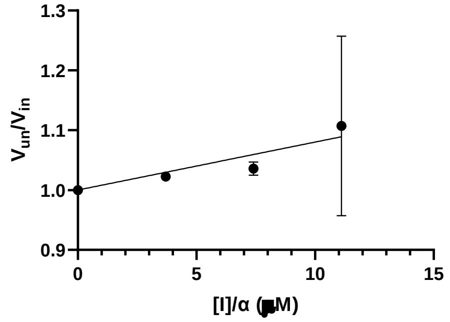

K_i = 125 ± 20 μM</td>
<td>No inhibition</td>
</tr>
<tr>
<td>20</td>
<td>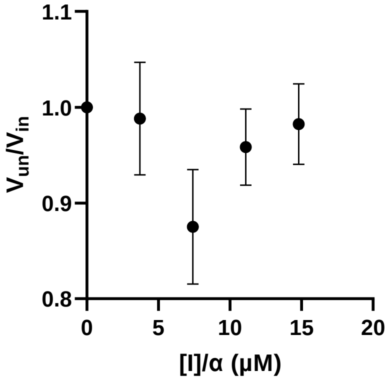

No inhibition</td>
<td>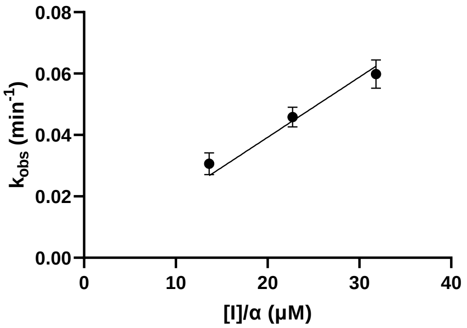

k_{inact}/K_I = 7422 ± 525 M^{-1} min^{-1}</td>
</tr>
<tr>
<td>21</td>
<td>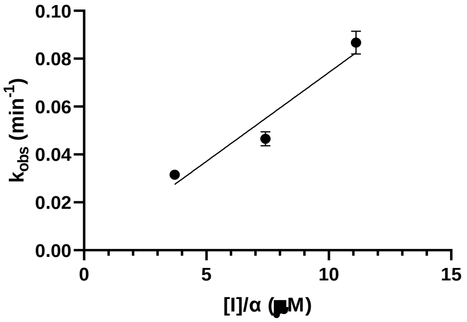

k_{inact}/K_I = 7422 ± 525 M^{-1} min^{-1}</td>
<td>No inhibition</td>
</tr>
<tr>
<td>22</td>
<td>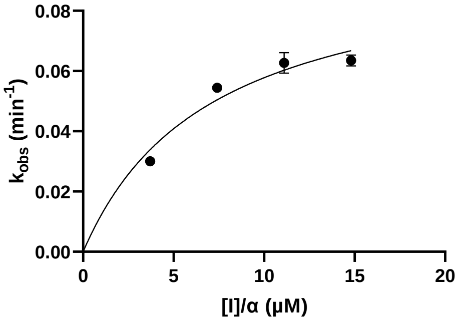

k_{inact}/K_I = 13922 ± 6664 M^{-1} min^{-1}
k_{inact} = 0.0988 ± 0.0185 min^{-1}
K_I = 7.1 ± 3.1 μM</td>
<td>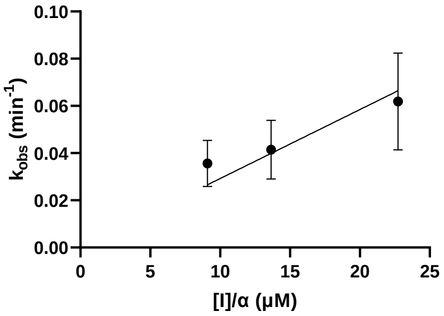

k_{inact}/K_I = 2922 ± 260 M^{-1} min^{-1}</td>
</tr>
</table>

<table>
<tr><td>23</td><td>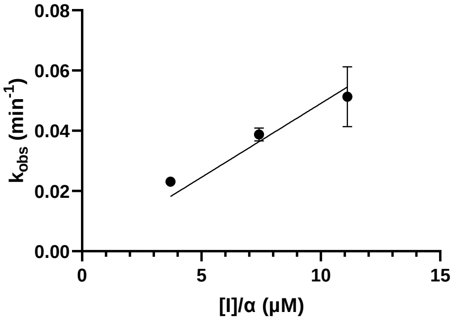

k_{inact}/K_I = 4907 ± 325 M^{-1} min^{-1}</td><td>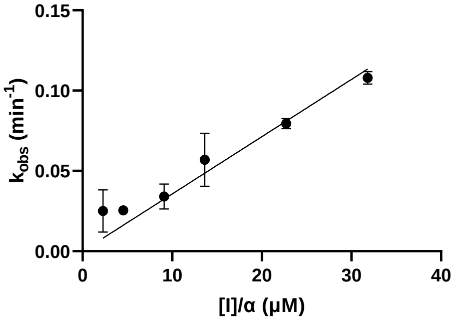

k_{inact}/K_I = 3565 ± 229 M^{-1} min^{-1}</td></tr>
<tr><td>45</td><td>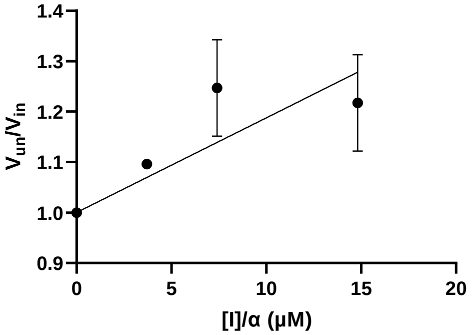

K_i = 53 ± 12 μM</td><td>No inhibition</td></tr>
<tr><td>46</td><td>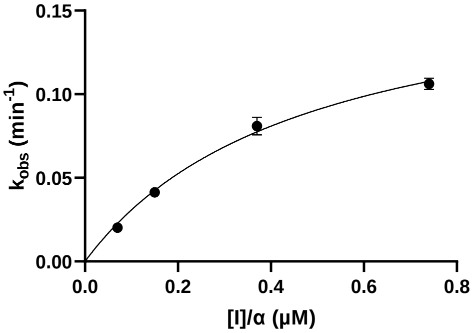

k_{inact}/K_I = 372992 ± 74839 M^{-1} min^{-1}
k_{inact} = 0.1765 ± 0.0160 min^{-1}
K_I = 0.4732 ± 0.0847 μM</td><td>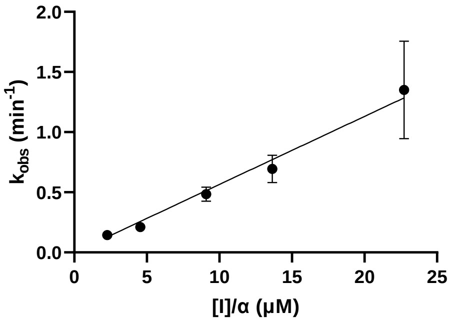

k_{inact}/K_I = 56520 ± 2044 M^{-1} min^{-1}</td></tr>
<tr><td>47</td><td>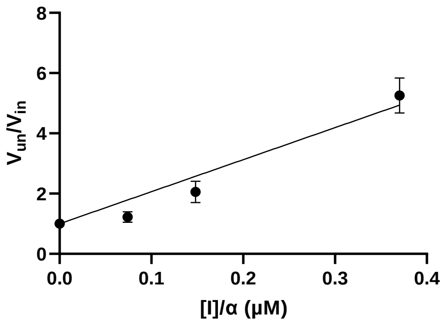

K_i = 0.0941 ± 0.0105 μM</td><td>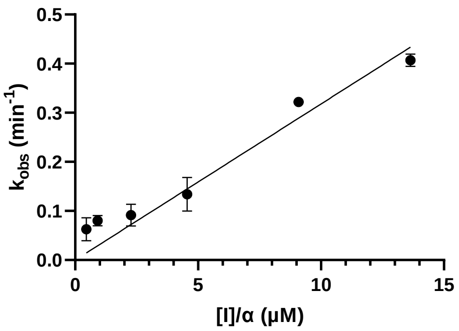

k_{inact}/K_I = 31780 ± 2218 M^{-1} min^{-1}</td></tr>
</table>

**71**	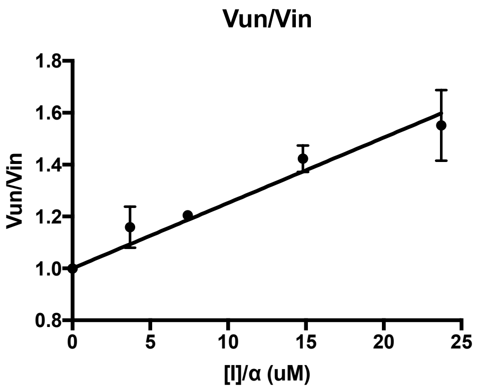<br>Slope = 0.02521 ± 0.001641<br>K_i = 39.7 ± 2.6 µM (*interference*)	No inhibition up to 360 µM
**79**	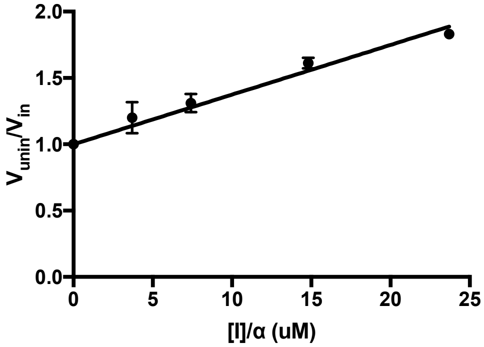<br>Slope = 0.03745 ± 0.001839<br>K_i = 26.7 ± 1.3 µM (*interference*)	No inhibition up to 360 µM
**75**	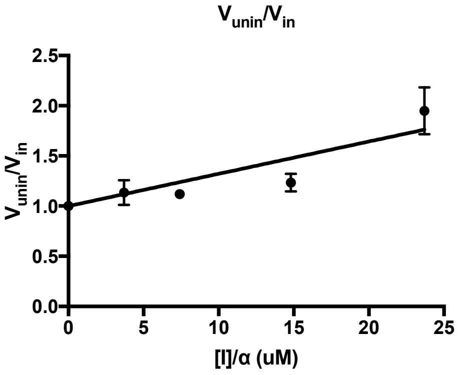<br>Slope = 0.03216 ± 0.00562<br>K_i = 31.1 ± 5.4 µM (*interference*)	No inhibition up to 360 µM

**74**	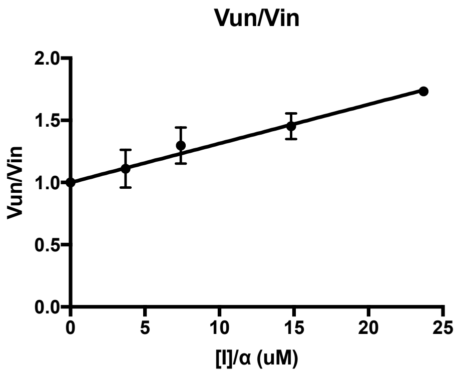<br>Slope = 0.03137 ± 0.001151<br>K_i = 31.9 ± 1.2 µM (*interference*)	No inhibition up to 360 µM
**73**	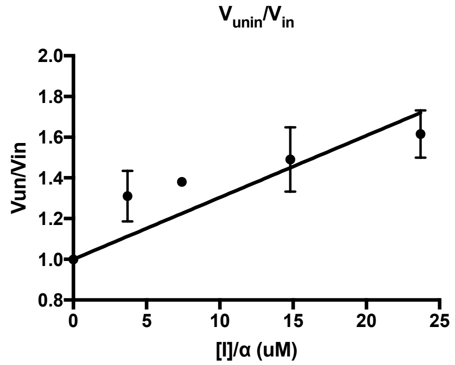<br>Slope = 0.03038 ± 0.004724<br>K_i = 32.9 ± 5.1 µM (*interference*)	No inhibition up to 360 µM
**76**	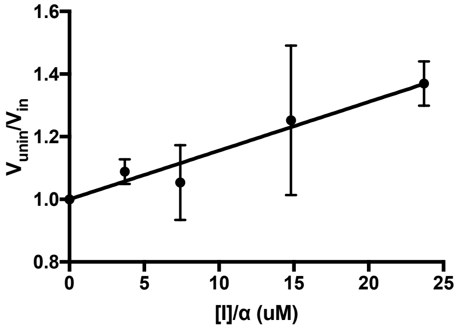<br>Slope = 0.01556 ± 0.001242<br>K_i = 64.3 ± 5.1 µM (*interference*)	No inhibition up to 360 µM

**72**	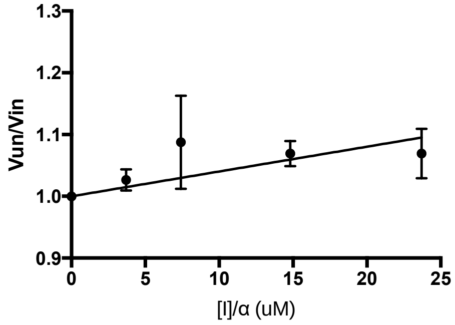<br>Slope = 0.004014 ± 0.001116<br>K_i = 249.1 ± 69.3 µM	No inhibition up to 360 µM
**77**	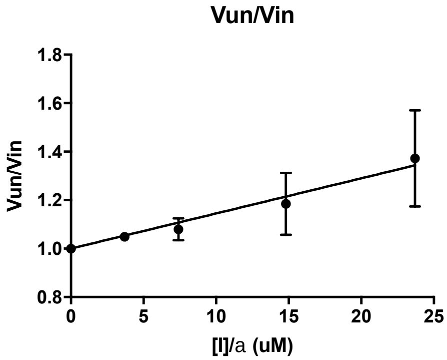<br>Slope = 0.01449 ± 0.0008536<br>K_i = 69.0 ± 4.1 µM	No inhibition up to 360 µM
**84**	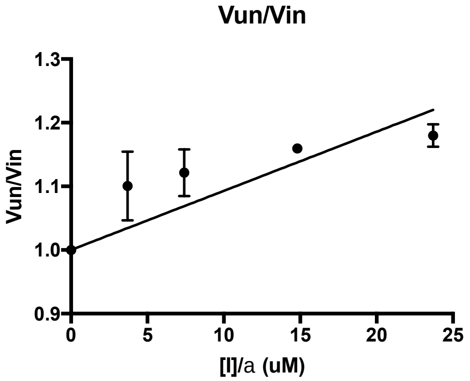<br>Slope = 0.009292 ± 0.00165<br>K_i = 107.6 ± 19.1 µM	No inhibition up to 360 µM

**87**	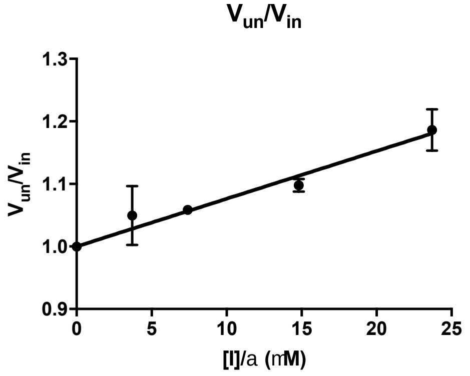<br>Slope = 0.007625 ± 0.0004603<br>K_i = 131.1 ± 7.9 µM	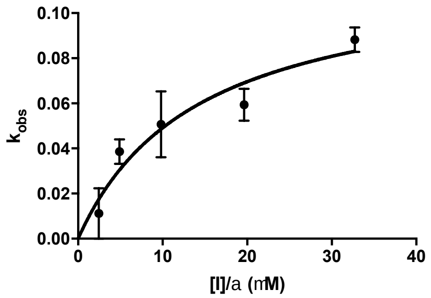<br>k_{inact}/K_I = 8350 ± 4488 M^{-1} min^{-1}<br>k_{inact} = 0.1194 ± 0.0262 min^{-1}<br>K_I = 14.3 ± 7.0 µM
**88**	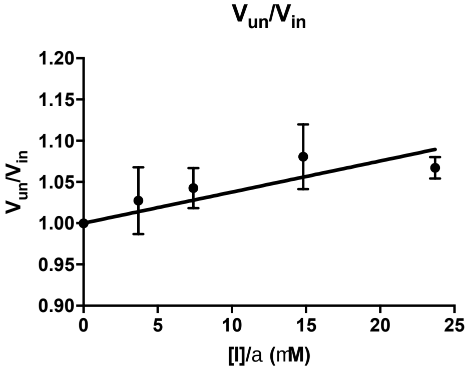<br>Slope = 0.003773 ± 0.0006661<br>K_i = 265.0 ± 46.8 µM	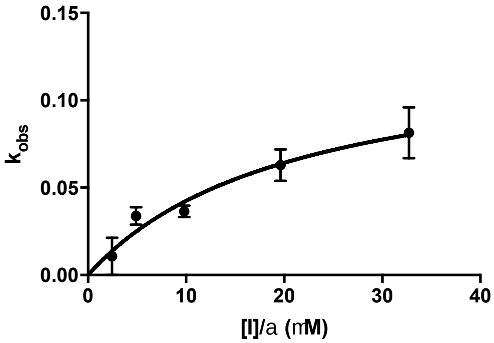<br>k_{inact}/K_I = 6245 ± 2973 M^{-1} min^{-1}<br>k_{inact} = 0.1322 ± 0.0288 min^{-1}<br>K_I = 21.17 ± 8.96 µM
**93 (KM93)**	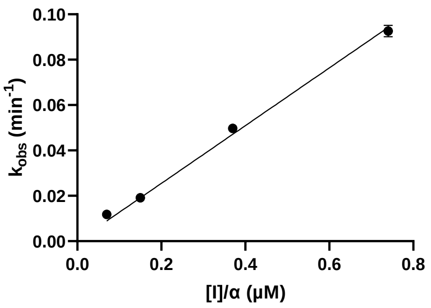<br>k_{inact}/K_I = 127300 ± 2890 M^{-1} min^{-1}	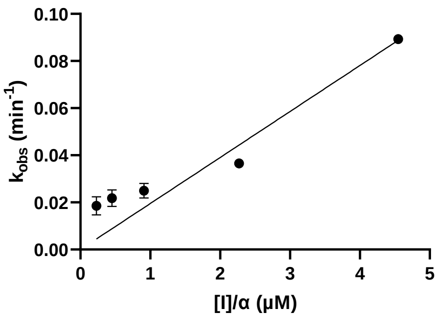<br>k_{inact}/K_I = 19520 ± 2108 M^{-1} min^{-1}

HPLC Traces of Peptidic Inhibitors

Compound 11. Ac-D-Asp-Dap(Acrylamide)-Nle-Nle-Leu-Pro-Trp-Pro-OH.

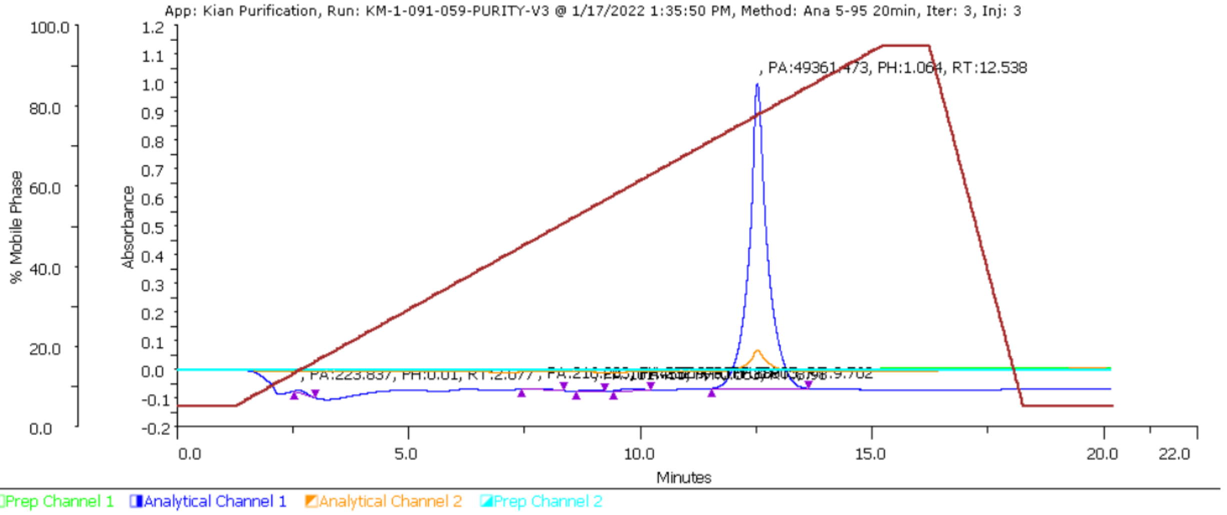

Sample Table

Injection Number	Peak Name	Retention Time (min)	Area (mAUmin x100)	Height (AU)	Sample Name	Sample Location	Fraction Site(s)	Area %
3	1	2.677	223.8372	0.01	KM-1-091redo	Sample Zone->163		0.446
3	2	12.538	49361.4728	1.064	KM-1-091redo	Sample Zone->163		98.416
3	3	9.762	252.9785	0.005	KM-1-091redo	Sample Zone->163		0.504
3	4	8.98	101.4551	0.003	KM-1-091redo	Sample Zone->163		0.202
3	5	7.888	216.2027	0.004	KM-1-091redo	Sample Zone->163		0.431

Compound 12. Ac-D-Asp-Dab(Acrylamide)-Nle-Nle-Leu-Pro-Trp-Pro-OH.

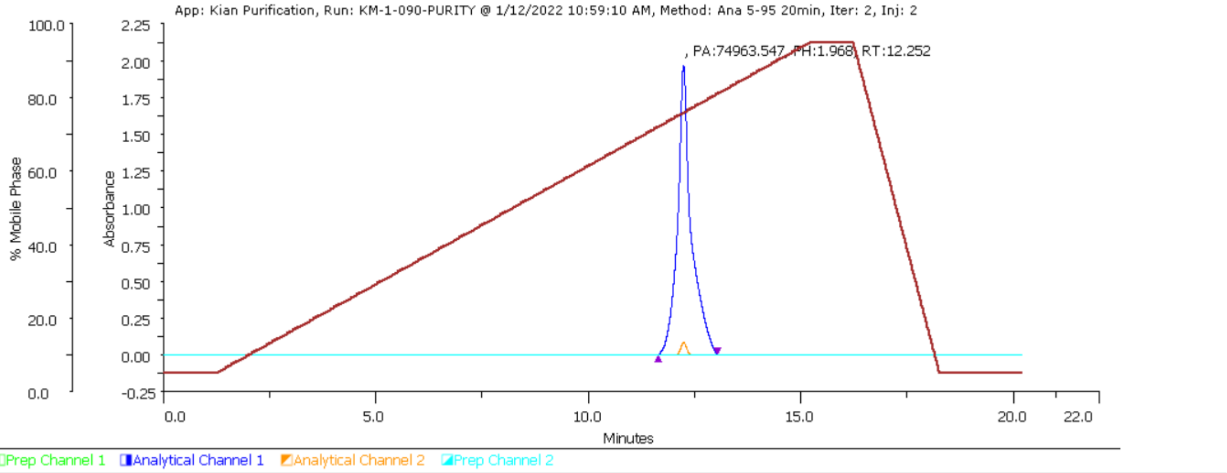

Sample Table

Injection Number	Peak Name	Retention Time (min)	Area (mAUmin x100)	Height (AU)	Sample Name	Sample Location	Fraction Site(s)	Area %
2	1	12.252	74963.5466	1.968	KM-1-090-PURITY	Sample Zone->162		100

Compound 13. Ac-D-Asp-Orn(Acrylamide)-Nle-Nle-Leu-Pro-Trp-Pro-OH.

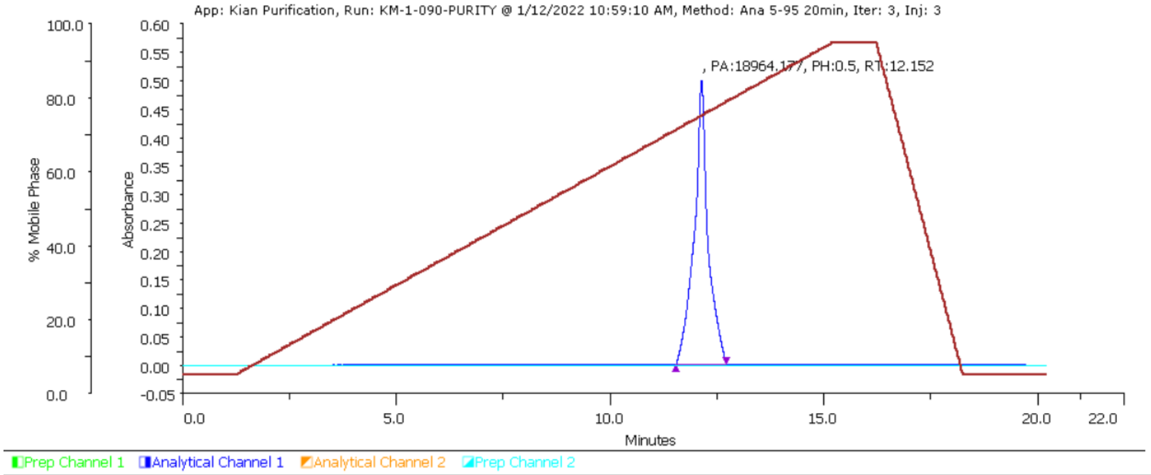

Sample Table

Injection Number	Peak Name	Retention Time (min)	Area (mAUmin x100)	Height (AU)	Sample Name	Sample Location	Fraction Site(s)	Area %
3	1	12.152	18964.1767	0.5	KM-1-043-PURITY	Sample Zone->162		100

Compound 14. Ac-D-Asp-Lys(Acrylamide)-Nle-Nle-Leu-Pro-Trp-Pro-OH.

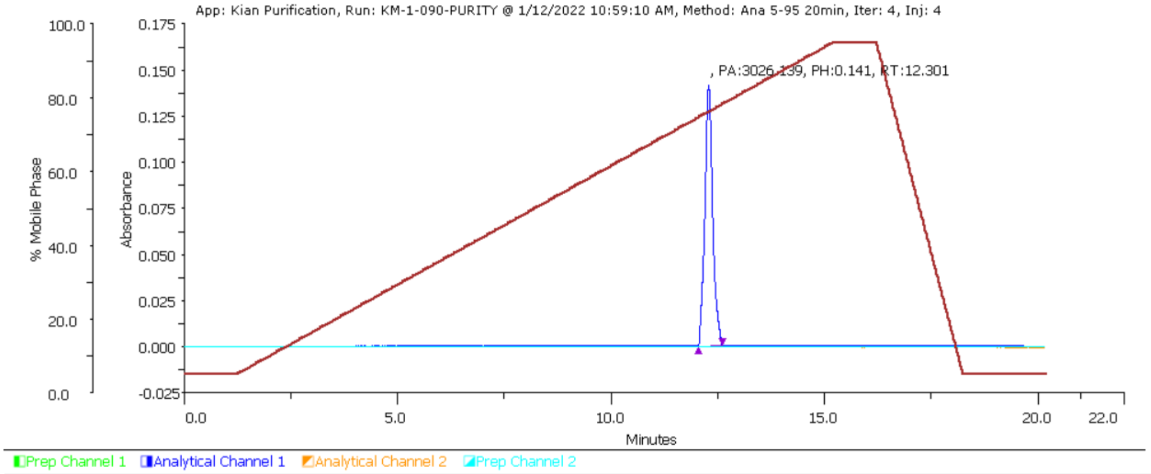

Sample Table

Injection Number	Peak Name	Retention Time (min)	Area (mAUmin x100)	Height (AU)	Sample Name	Sample Location	Fraction Site(s)	Area %
4	1	12.301	3026.1386	0.141	KM-1-042-PURITY	Sample Zone->162		100

Compound 20. Ac-D-Asp-Dap(α-chloroacetamide)-Nle-Nle-Leu-Pro-Trp-Pro-OH.

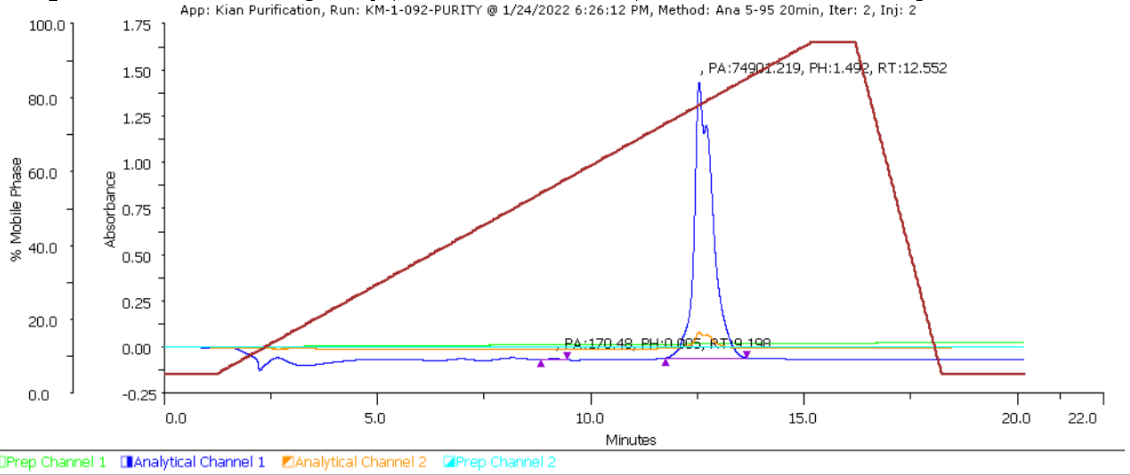

Sample Table

Injection Number	Peak Name	Retention Time (min)	Area (mAUmin x100)	Height (AU)	Sample Name	Sample Location	Fraction Site(s)	Area %
2	1	12.552	74901.2188	1.492	KM-1-092-PURITY	Sample Zone->163		99.773
2	2	9.198	170.4799	0.005	KM-1-092-PURITY	Sample Zone->163		0.227

Compound 21. Ac-D-Asp-Dab(α-chloroacetamide)-Nle-Nle-Leu-Pro-Trp-Pro-OH.

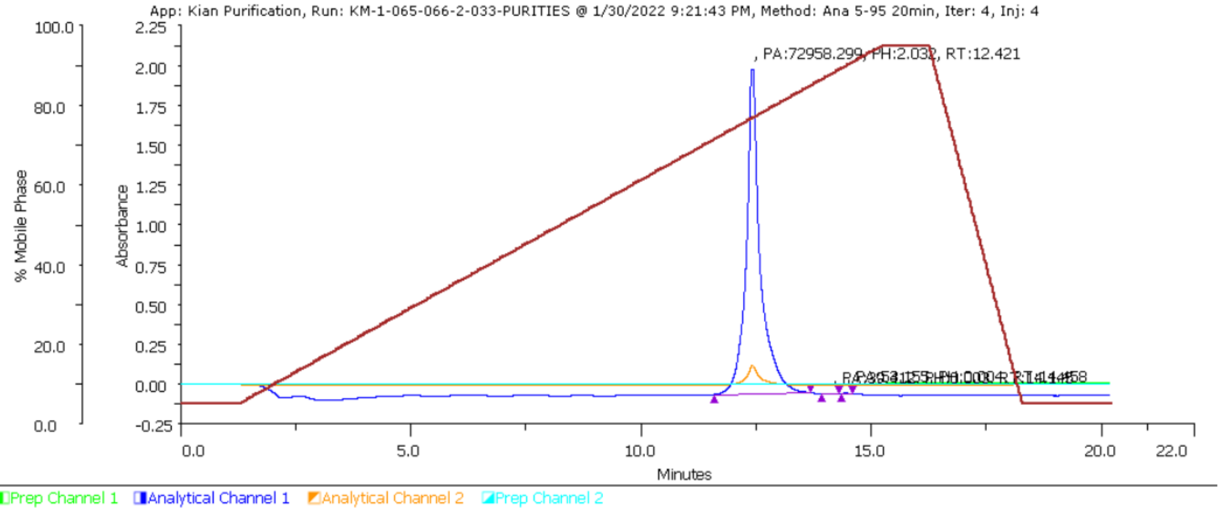

Sample Table

Injection Number	Peak Name	Retention Time (min)	Area (mAUmin x100)	Height (AU)	Sample Name	Sample Location	Fraction Site(s)	Area %
4	1	12.421	72958.2993	2.032	KM-1-065	Sample Zone->150		99.873
4	2	14.458	53.1548	0.004	KM-1-065	Sample Zone->150		0.073
4	3	14.145	39.4118	0.003	KM-1-065	Sample Zone->150		0.054

Compound 22. Ac-D-Asp-Orn(α-chloroacetamide)-Nle-Nle-Leu-Pro-Trp-Pro-OH.

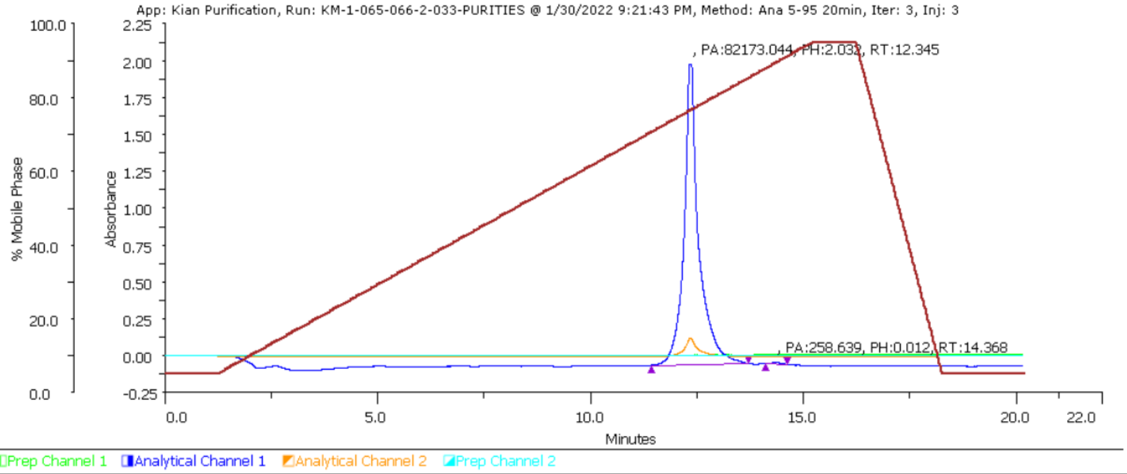

Sample Table

Injection Number	Peak Name	Retention Time (min)	Area (mAUmin x100)	Height (AU)	Sample Name	Sample Location	Fraction Site(s)	Area %
3	1	12.345	82173.0436	2.032	KM-1-066	Sample Zone->149		99.686
3	2	14.368	258.6394	0.012	KM-1-066	Sample Zone->149		0.314

Compound 23. Ac-D-Asp-Lys(α-chloroacetamide)-Nle-Nle-Leu-Pro-Trp-Pro-OH.

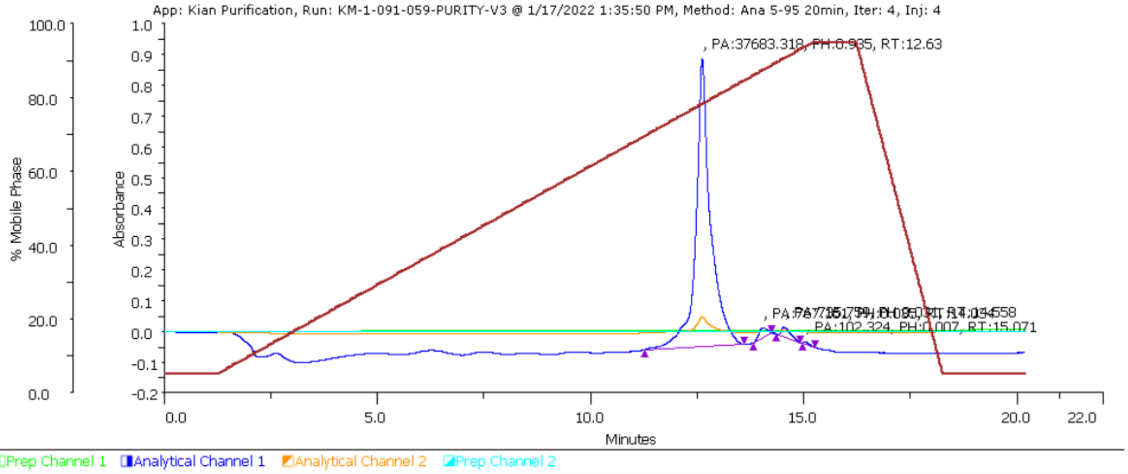

Sample Table

Injection Number	Peak Name	Retention Time (min)	Area (mAUmin x100)	Height (AU)	Sample Name	Sample Location	Fraction Site(s)	Area %
4	1	12.63	37683.3183	0.935	KM-1-059	Sample Zone->163		95.914
4	2	14.034	767.1615	0.035	KM-1-059	Sample Zone->163		1.953
4	3	14.558	735.7587	0.031	KM-1-059	Sample Zone->163		1.873
4	4	15.071	102.3235	0.007	KM-1-059	Sample Zone->163		0.26

Compound 45. Ac-D-Asp-Asp(MA)-Nle-Nle-Leu-Pro-Trp-Pro-OH.

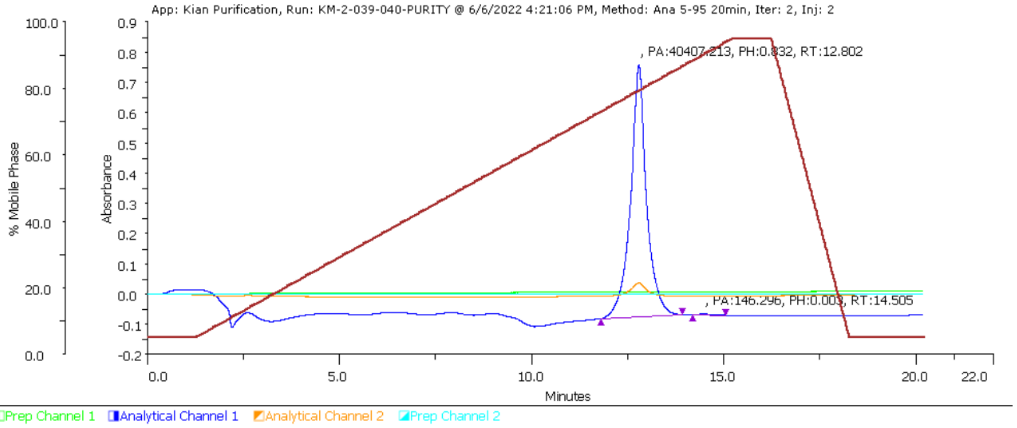

Sample Table

Injection Number	Peak Name	Retention Time (min)	Area (mAUmin x100)	Height (AU)	Sample Name	Sample Location	Fraction Site(s)	Area %	
2	1	12.802	40407.2126	0.832	KM-2-039-PURITY	Sample Zone->160		99.639	
2	2	14.505	146.2959	0.003	KM-2-039-PURITY	Sample Zone->160		0.361	

Compound 46 (ZED1301). Ac-D-Asp-Glu(MA)-Nle-Nle-Leu-Pro-Trp-Pro-OH.

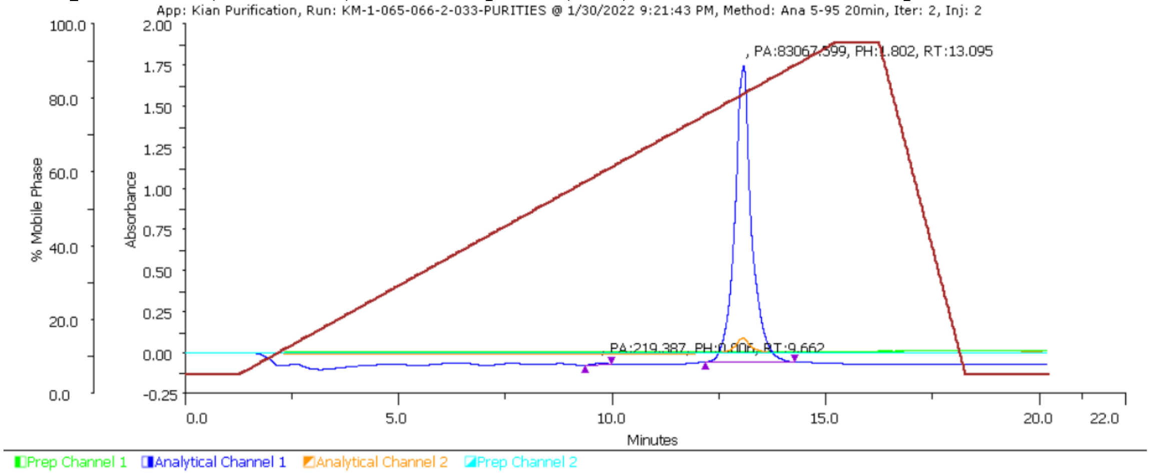

Sample Table

Injection Number	Peak Name	Retention Time (min)	Area (mAUmin x100)	Height (AU)	Sample Name	Sample Location	Fraction Site(s)	Area %
2	1	13.095	83067.5993	1.802	KM-2-033	Sample Zone->148		99.737
2	2	9.662	219.3874	0.006	KM-2-033	Sample Zone->148		0.263

Compound 47. Ac-D-Asp-Hmg(MA)-Nle-Nle-Leu-Pro-Trp-Pro-OH.

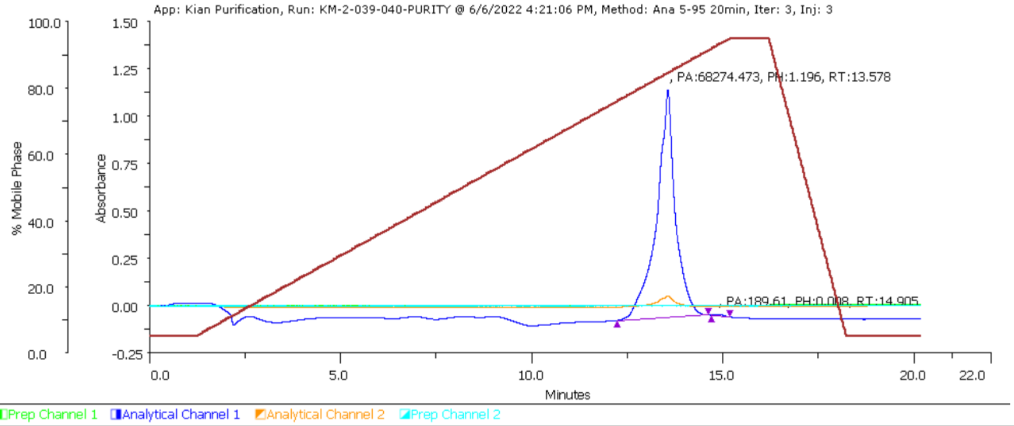

Sample Table

Injection Number	Peak Name	Retention Time (min)	Area (mAUmin x100)	Height (AU)	Sample Name	Sample Location	Fraction Site(s)	Area %	
3	1	13.578	68274.4729	1.196	KM-2-040-PURITY	Sample Zone->161		99.723	
3	2	14.905	189.6096	0.008	KM-2-040-PURITY	Sample Zone->161		0.277	

Compound 93 (KM93). RhB-Pro-6AH-D-Asp-Glu(MA)-Nle-Nle-Leu-Pro-Trp-Pro-OH.

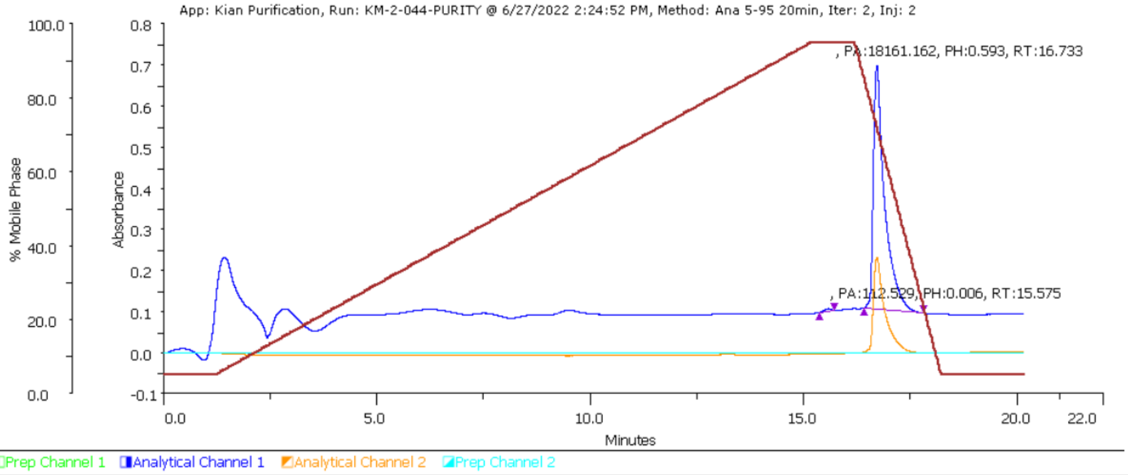

Sample Table

Injection Number	Peak Name	Retention Time (min)	Area (mAUmin x100)	Height (AU)	Sample Name	Sample Location	Fraction Site(s)	Area %
2	1	15.575	112.5295	0.006	KM-2-044-PURITY	Sample Zone->163		0.616
2	2	16.733	18161.1618	0.593	KM-2-044-PURITY	Sample Zone->163		99.384

References

42. McNeil, N.M.R.; Gates, E.W.J.; Firoozi, N.; Cundy, N.J.; Leccese, J.; Eisinga, S.; Tyndall, J.D.A.; Adhikary, G.; Eckert, R.L.; Keillor, J.W. Structure-Activity Relationships of N-Terminal Variants of Peptidomimetic Tissue Transglutaminase Inhibitors. *Eur J Med Chem* **2022**, *232*, 114172, doi:10.1016/j.ejmech.2022.114172.
51. Hils, M.; Pasternack, R.; Büchold, C.; Weber, J.; Heine, A.; Klebe, G.; Stieler, M. Crystal Structure of Blood Coagulation Factor XIIIa. WO2014090835A1 **2014**.
53. Büchold, C.; Gerlach, U.; Hils, M.; Pasternack, R.; Weber, J. Pyridinone Derivatives as Tissue Transglutaminase Inhibitors. WO2014012858A1 **2014**.
65. Adamczyk, M.; Johnson, D.D.; Reddy, R.E. Collagen Cross-Links: A Convenient Synthesis of Tert-Butyl-(2S)-2-[(Tert-Butoxycarbonyl)Amino]-4-(2-Oxiranyl)Butanoate. *Tetrahedron Asymmetry* **1999**, *10*, 775–781, doi:10.1016/S0957-4166(99)00055-5.
66. Kokotos, G.; Padrón, J.M.; Martín, T.; Gibbons, W.A.; Martín, V.S. A General Approach to the Asymmetric Synthesis of Unsaturated Lipidic α-Amino Acids. the First Synthesis of α-Aminoarachidonic Acid. *J Org Chem* **1998**, *63*, 3741–3744, doi:10.1021/jo9715128.
67. Olsen, R.K.; Ramasamy, K.; Emery, T. Synthesis of N.alpha.,N.delta.-protected N.delta.-hydroxy-L-ornithine from L-glutamic acid. *J Org Chem* **1984**, *49*, 3527–3534, doi:10.1021/jo00193a016.
68. Goode, D.R.; Sharma, A.K.; Hergenrother, P.J. Using Peptidic Inhibitors to Systematically Probe the S1′ Site of Caspase-3 and Caspase-7. *Org Lett* **2005**, *7*, 3529–3532, doi:10.1021/ol051287d.
69. Fulmer, G.R.; Miller, A.J.M.; Sherden, N.H.; Gottlieb, H.E.; Nudelman, A.; Stoltz, B.M.; Bercaw, J.E.; Goldberg, K.I. NMR Chemical Shifts of Trace Impurities: Common Laboratory Solvents, Organics, and Gases in Deuterated Solvents Relevant to the Organometallic Chemist. *Organometallics* **2010**, *29*, 2176–2179, doi:10.1021/om100106e.
70. Cai, H.; Guengerich, F.P. Mechanism of Aqueous Decomposition of Trichloroethylene Oxide. *J Am Chem Soc* **1999**, *121*, 11656–11663, doi:10.1021/ja9914240.
71. Narita, K.; Matsuhara, K.; Itoh, J.; Akiyama, Y.; Dan, S.; Yamori, T.; Ito, A.; Yoshida, M.; Katoh, T. Synthesis and Biological Evaluation of Novel FK228 Analogues as Potential Isoform Selective HDAC Inhibitors. *Eur J Med Chem* **2016**, *121*, 592–609, doi:10.1016/J.EJMECH.2016.05.031.
72. Eissler, S.; Kley, M.; Bächle, D.; Loidl, G.; Meier, T.; Samson, D. Substitution Determination of Fmoc-Substituted Resins at Different Wavelengths. *J Pept Sci* **2017**, *23*, 757–762, doi:10.1002/PSC.3021.
73. Neises, B.; Steglich, W. Simple Method for the Esterification of Carboxylic Acids. *Angew Chem Int Ed Engl* **1978**, *17*, 522–524, doi:10.1002/ANIE.197805221.
74. Li, X.; Atkinson, R.N.; Bruce King, S. Preparation and Evaluation of New L-Canavanine Derivatives as Nitric Oxide Synthase Inhibitors. *Tetrahedron* **2001**, *57*, 6557–6565, doi:10.1016/S0040-4020(01)00547-6.
75. Zhang, C.; Wu, S.; Xi, Z.; Yi, L. Design and Synthesis of NBD-S-Dye Dyads for Fluorescently Discriminative Detection of Biothiols and Cys/Hcy. *Tetrahedron* **2017**, *73*, 6651–6656, doi:10.1016/j.tet.2017.10.020.
76. Vachal, P.; Fletcher, J.M.; Fong, T.M.; Huang, C.C.R.R.; Lao, J.; Xiao, J.C.; Shen, C.P.; Strack, A.M.; Shearman, L.; Stribling, S.; et al. 1-Sulfonyl-4-Acylpiperazines as Selective

Cannabinoid-1 Receptor (CB1R) Inverse Agonists for the Treatment of Obesity. *J Med Chem* **2009**, *52*, 2550–2558, doi:10.1021/jm900063x.

77. Sousbie, M.; Vivancos, M.; Brouillette, R.L.; Besserer-Offroy, É.; Longpré, J.M.; Leduc, R.; Sarret, P.; Marsault, É. Structural Optimization and Characterization of Potent Analgesic Macrocyclic Analogues of Neurotensin (8-13). *J Med Chem* **2018**, *61*, 7103–7115, doi:10.1021/acs.jmedchem.8b00175.
